# Subthreshold Vibration Influences Standing Balance but Has Unclear Impact on Somatosensation in Persons With Transtibial Amputations

**DOI:** 10.3389/fphys.2022.810079

**Published:** 2022-02-02

**Authors:** Zachary S. Meade, Aaron D. Likens, Jenny A. Kent, Kota Z. Takahashi, Shane R. Wurdeman, Adam L. Jacobsen, Manuel E. Hernandez, Nick Stergiou

**Affiliations:** ^1^Carle Illinois College of Medicine, University of Illinois Urbana-Champaign, Urbana, IL, United States; ^2^Department of Biomechanics, University of Nebraska Omaha, Omaha, NE, United States; ^3^Department of Physical Therapy, University of Nevada, Las Vegas, Las Vegas, NV, United States; ^4^Clinical and Scientific Affairs, Hanger Clinic, Austin, TX, United States; ^5^Prosthetics and Sensory Aids, Veterans Affairs Medical Center, Omaha, NE, United States; ^6^Department of Kinesiology and Community Health, College of Applied Health Sciences, University of Illinois Urbana-Champaign, Urbana, IL, United States

**Keywords:** transtibial amputation, pink noise, white noise, stochastic resonance, vibration, somatosenation, balance, mobility

## Abstract

Stochastic resonance has been successfully used to improve human movement when using subthreshold vibration. Recent work has shown promise in improving mobility in individuals with unilateral lower limb amputations. Furthering this work, we present an investigation of two different signal structures in the use of stochastic resonance to improve mobility in individuals with unilateral lower limb amputations. Cutaneous somatosensation and standing balance measures using spatial and temporal analysis were assessed. There were no differences in the somatosensation measures, but differences in the temporal characteristics of the standing measures were seen with the various vibration structures when compared to no vibration, one of which suggesting mass may play an important role in determining who may or may not benefit from this intervention. Stochastic resonance employed with subthreshold vibration influences mobility in individuals with unilateral amputations, but the full direction and extent of influence is yet to be understood.

## Introduction

Across the globe, lower limb amputations occur daily and are life altering events that may have grave consequences on an individual’s mental and physical state (Grzebień et al., 2017). Amputations are associated with balance deficits, an increased fall risk, morbidity, mortality, and decreased quality of life (Ku et al., 2014; Grzebień et al., 2017). A notable morbidity secondary to the decreased mobility from amputations is obesity. Increased mass influences posture, and may have a synergistic effect with balance disturbances seen with unilateral lower limb amputations (Hlavackova et al., 2011; Costello et al., 2012). To address these issues, patients go through physical rehabilitation focused on modified gait strategies, proprioception exercises, and other physical therapy. Once stable, individuals are fitted for a prosthesis, which is a vital tool improving mobility and overall quality of life (Ku et al., 2014; Grzebień et al., 2017). Despite technological advances over the past years, prosthetic devices remain imperfect substitutes for biological limbs for the demands of daily life. Most commercially available prostheses have mechanical deficits, lack voluntary control, and lack sensory feedback from the foot and ankle, reducing the ability to ambulate (Price et al., 2019). For example, reduced sensory input is one factor related to impaired balance in persons with a transtibial amputation because the residual limb is required to take on a foreign sensory role with structures that are less sensitive (Quai et al., 2005; Ku et al., 2014). Additionally, the diminished somatosensory feedback imposes more reliance on visual feedback to maintain balance (Fernie and Holliday, 1978; Vanicek et al., 2009). Impaired balance may lead to individuals avoiding certain activities, compounding some morbidities (Patla and Shumway-Cook, 1999). However, improving the somatosensation of the residual limb may improve standing balance (Quai et al., 2005; Likens et al., 2020).

Some noteworthy interventions to improve somatosensation use stochastic resonance (SR). The idea of enhancing an underlying signal with noise (e.g., vibration) defines SR. The underlying principle suggests that applying subthreshold (i.e., imperceivable) vibration, typically white noise, to a non-linear system can enhance an underlying signal of interest by increasing the signal-to-noise ratio (SNR; Gammaitoni et al., 1998; McDonnell and Abbott, 2009; Sejdić and Lipsitz, 2013). This may seem paradoxical because noise is traditionally characterized as error. However, when an optimal amount of noise is applied to a non-linear system, the noise signal becomes additive to the signal of interest resulting in a newly perceivable signal (Gammaitoni et al., 1998; McDonnell and Abbott, 2009; Sejdić and Lipsitz, 2013).

Stochastic resonance is found artificially and natively within humans (Krauss et al., 2016). For example, it has been suggested that the vestibular system uses SR to amplify sound to ward off hearing loss, presenting as tinnitus, or conductive hearing loss (Krauss et al., 2016). In the context of human movement, applying subthreshold vibration at or proximal to the site of interest has been associated with improvements in balance, proprioception, gait, and sensation (Gravelle et al., 2002; Liu et al., 2002; Khaodhiar et al., 2003; Priplata et al., 2006; Stephen et al., 2012; Enders et al., 2013; Kundu and Sarkar, 2015; Lakshminarayanan et al., 2015; Lipsitz et al., 2015; van der Groen and Wenderoth, 2016; Hathibelagal, 2018; Temple et al., 2018; Seo et al., 2019; Zandiyeh et al., 2019; Likens et al., 2020; Zwaferink et al., 2020). Notable areas of improvement in individuals with amputation include SR reducing COP sway, gait variability, and single leg stances measures (Lee et al., 2007; Likens et al., 2020). Given previous promising results, SR may be beneficial to improve other measures of standing balance and somatosensation in individuals with amputations (Likens et al., 2020).

While there have been some supporting results with the use of SR, there has been minimal investigation into various signal structures used to implement SR. Aghababaiyan (2020) and Likens et al. (2020) are notable exceptions. Both investigators compared two different signal structures: white and pink vibration. White noise is defined as noise having equal intensities of frequencies with a constant power spectrum. Contrasting to white noise, pink noise has a power spectral density of $\left(\frac{1}{f^{\beta }}\right)$, where *f* = frequency, and β = (0, 1). Pink noise can be described as a signal that demonstrates positive autocorrelation (i.e., large values tend to be followed by a large values; small values tend to followed by small values), containing temporally related self-similarity; which is prevalent in healthy human processes but commonly absent or degraded in pathological processes (Hausdorff et al., 1997; Ivanov et al., 2001; Goldberger et al., 2002; Stergiou et al., 2006; Diniz et al., 2011; Kaipust et al., 2012, 2013; Cavanaugh et al., 2017). Pink noise has been investigated in numerous applications with many encouraging results.

Work by Nozaki et al. (1999) showed that pink noise may enhance weak cutaneous sensory neuron signals in rats more effectively than white noise, and more recently Aghababaiyan (2020) demonstrated via modeling that pink noise was 25 times more effective than white noise in amplifying neuronal signals. An *in vivo* study by Mutch et al. (2005) showed that adding pink noise to ventilator breathing patterns improved ventilation-perfusion matching. Recently pink noise was demonstrated to improve memory and sleep patterns in healthy older adults and those with cognitive impairment (Papalambros et al., 2017, 2019). In terms of human movement Likens et al. (2020) demonstrated subthreshold vibration reduced mediolateral COP range and COP RMS with pink vibration, while white vibration was associated with reduced step length variability, demonstrating an important difference between the two signal structures is worthy of investigation. The mechanism of pink noise has been hypothesized to be related to intrinsic characteristics that better mirror physiological signals, and specifically its local non-stationary properties (Nozaki et al., 1999; Mutch et al., 2005; Aghababaiyan, 2020; Likens et al., 2020). While Likens et al. (2020) compared the difference of white and pink noise structures as it relates to standing and walking, basic somatosensation has yet to be explored in persons with unilateral lower limb amputations. Understanding how pink and white noise structures influence somatosensation as it relates to SR is an important next step addressed by the current work. Lastly, given that noise structures appear to affect different groups in various ways, it is important to understand how individual differences in physiological covariates (e.g., mass, height) contribute to those differences (Kelty-Stephen et al., 2013). That is, meaningful physical characteristics that are known to influence postural control may contribute additional explanatory power toward understanding stochastic resonance effects in diverse groups.

### Purpose and Hypothesis

The purpose of this study was to investigate the effects of subthreshold pink and white vibration on measures of somatosensation, and standing balance to determine the potential impact of subthreshold vibration in improving mobility of individuals with a unilateral transtibial amputation.

It was hypothesized that applying subthreshold vibration proximally to the affected limb of individuals with transtibial amputation would lead to an improvement in somatosensation and standing balance. Furthermore, it was hypothesized that subthreshold pink vibration signals would induce a greater improvement in somatosensation, and standing balance when compared to subthreshold white vibration signals.

## Materials and Methods

### Subjects

Twenty-one adults with a unilateral transtibial amputation (Table 1) were recruited from prosthetics clinics in the eastern Nebraska area and the Omaha Veteran-Affairs Medical Center. Exclusion criteria included: amputation less than 6 months ago; self-reported disorder that affects gait or balance (except diabetes); current pregnancy; current open sores on residual limb.

**TABLE 1:** Demographic information for persons with a transtibial amputation and amputation information.

Cause of amputation	*n*	Age (years)	Mass (kg)	Height (m)	Time since amputation (years)	Gender (Female/Male)
All	20	58.9 (15.0)	101 (15.2)	1.80 (0.065)	9.44 (7.29)	4F/16M
Trauma	8	51.9 (15.1)	102 (18.0)	1.80 (0.068)	7.53 (4.21)	2F/6M
Diabetes related	4	63.0 (13.3)	97.6 (15.1)	1.80 (0.023)	6.88 (5.11)	0F/4M
Infection (non-diabetes related)	4	66.0 (6.98)	106 (19.9)	1.79 (0.072)	7.25 (6.70)	1F/3M
Other	4	62.0 (20.6)	98.3 (4.95)	1.80 (0.099)	18 (10.1)	1F/3M

*“Other” includes causes such as cancer, arthritic complications, and congenital cases (n = 1). One participant did not complete the study.*

Informed consent was obtained from each participant prior to participation. Institutional Review Board approval for the study protocol was obtained from the University of Nebraska Medical Center and the VA Nebraska-Western Iowa Health Care System.

### Intervention

**FIGURE 1:**
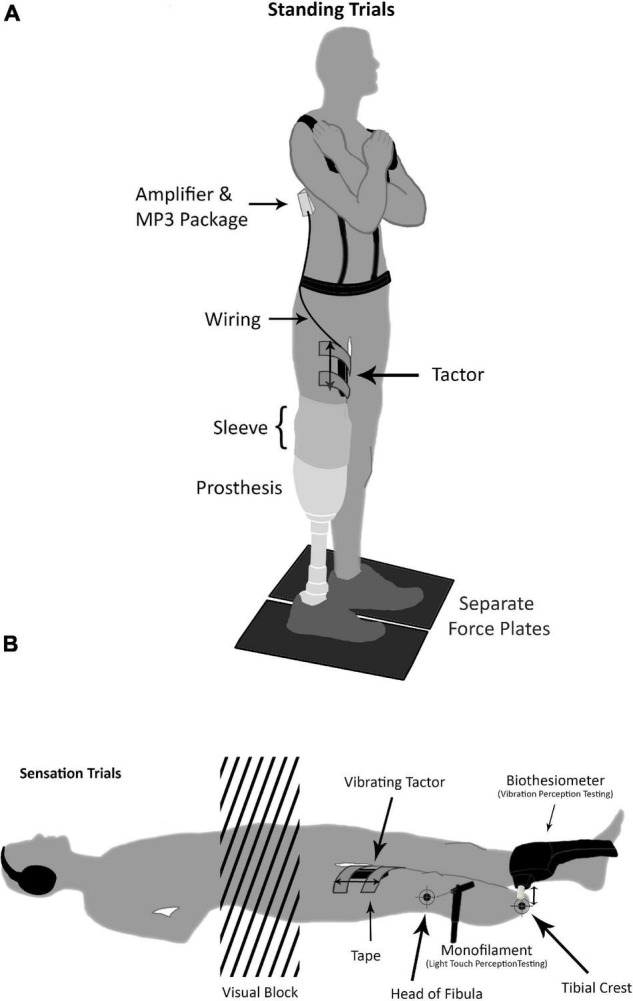
**(A)** All participants underwent standing trials of 90 and 30 s with eyes opened and closed, respectively. Arms were crossed and eyes were orientated on a fixation cross while standing on parallel force plates. The tactor unit was secured with tape and placed above the prosthetic socket and sleeve (if applicable). The tactor was wired to an amplifier and MP3 player that was mounted and secured in a black package attached to a harness. **(B)** Double sided arrows point in the direction of vibration. For the tactor, this is parallel to the thigh, and for the biothesiometer, this is perpendicular to the leg. Positioning of participants during light touch and vibration perception testing with the two test sites marked. The monofilaments were used for light touch perception testing, and the Biothesiometer was used for vibration perception testing. During the head of fibula vibration testing the participant would rotate 90° onto the side of the intact limb allowing the Biothesiometer to rest perpendicular on the head of the fibula. Of note, this diagram depicts multiple tests being conducted, which did not occur simultaneously, rather sequentially with a washout period in between.

Twenty-minute pink noise and white noise signals were generated and converted to MP3 tracks in Audacity^®^ software (Version 2.1.6, 2016). Vibrations were generated using a lightweight commercially available tactor (Tactuator BM3C, Tactile Labs, Montreal, QC, Canada), connected via an in-house built amplifier to a digital MP3 player (Olympus, Center Valley, PA, United States). The frequency content of each signal was confirmed using an oscilloscope and accelerometer, with the tactor suspended in isolation. To account for minor non-linearities, a filter envelope was created based on the white noise signal, designed to obtain a flat band pass between 40 and 500 Hz on output. The filter envelope was then applied to pink and white noise signals and the output of the tactor was reverified when suspended and when attached to the skin via adhesive tape. The tactor was secured parallel to the thigh of the affected limb above the prosthetic sleeve using hypoallergenic double-sided tape with surgical tape overtop, permitting vibration along the longitudinal axis of the device (Figure 1).

The threshold of vibration perception of each participant was determined using a digital MP3 player with discrete measurements for each signal (pink, white). This was done individually using a 4-2-1 method (Dyck et al., 1993). Specifically, the vibration amplitude was increased in four-level discrete increments until the participant indicated they could feel the vibration. It was then decreased in two-level decrements until the participant indicated they couldn’t perceive the vibration. Finally, it was increased in single-level increments until the participant indicated they could perceive the vibration. That was the final level recorded. This process was repeated three times, and the lowest of these values was recorded as the threshold of perception for that signal (pink, white). During this process, the participant stood with one hand on a walking frame for support, fixating on a wall-mounted cross and wearing sound-blocking headphones.

Vibration amplitude was then viewed using an oscilloscope and decreased by 1–2 increments, guided by the oscilloscope reading, such that the vibration levels were set between 60 and 90% of the threshold of perception; a range similar to values reported in literature, which suggest that SR can be achieved using the MP3 players discrete volume adjustments (Liu et al., 2002; Collins et al., 2003; Priplata et al., 2003, 2006; Enders et al., 2013; Lipsitz et al., 2015).

### Procedure

**FIGURE 2:**
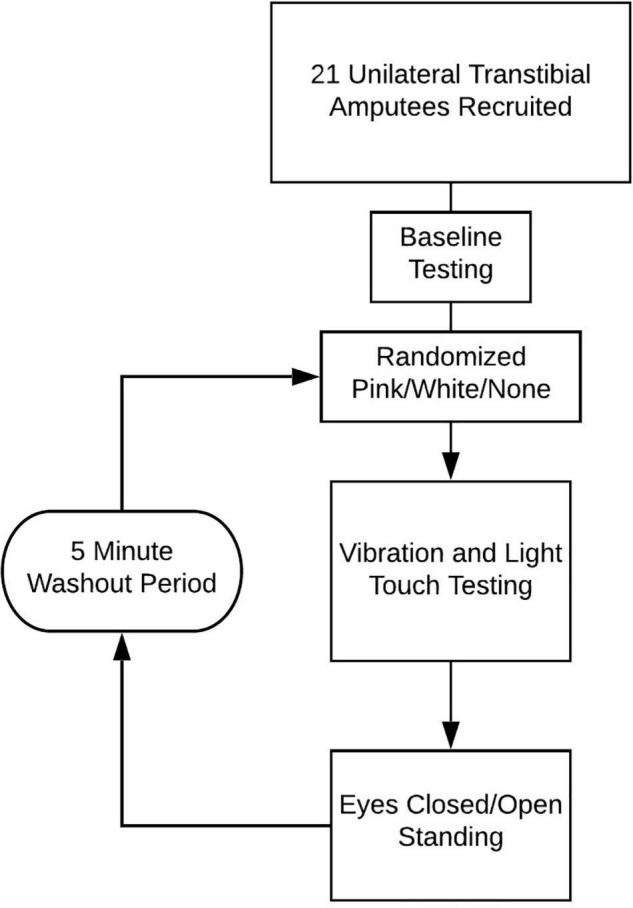
Diagram of session workflow.

As part of a larger study, participants were assessed for the effects of subthreshold vibration on somatosensation and standing balance (Figure 2). Sensation and standing testing consisted of a baseline condition with no vibration, followed by three double blinded-randomized conditions that included pink noise vibration, white noise vibration, and no vibration (none). Each condition was preceded with a 5 min washout period.

All participants underwent light touch and vibration perception tests at the head of the fibula and the distal end of the tibial crest (Figure 1). These sites were chosen due to their importance in fitting and load bearing within the prosthetic socket (Portnoy et al., 2008). Prosthetists intentionally offload certain areas to avoid irritation, and injury. Without modification, the head of the fibula and the apex of the amputated limb (i.e., tibial crest) tend to demonstrate the highest levels of shear and pressure (Portnoy et al., 2008). Both tests were conducted with sound-blocking headphones and a visual block (screen), preventing auditory or visual cues. The same assessors administered all tests. Anthropometric data such as age, height, and weight were collected.

### Light Touch Perception

Light touch perception threshold was tested using a set of 20 Semmes-Weinstein monofilaments (Touch Test^®^, North Coast Medical Inc., Gilroy, CA, United States) (Dyck et al., 1993; Bell-Krotoski et al., 1995). The monofilament fibers have a range of diameters each of which buckles under a certain amount of force (measured in grams) that increases as the diameter of the monofilament increases (Bell-Krotoski et al., 1995; Armstrong et al., 1998; Mayfield and Sugarman, 2000). The monofilaments were demonstrated on the back of the participant’s hand prior to testing. Participants laid supine and were instructed to indicate when they felt a light touch. The trials started at a thin monofilament size of 3.22 (0.16 g), expected to be below the perception threshold on the lower limb for most participants. Following the 4-2-1 stepping algorithm, as described earlier, the assessor increased and decreased the thickness of the monofilaments until the participant reported a light touch. Initially increasing in increments of 4 until a monofilament went from being not felt to felt, this was marked as a “turn around point.” The assessor then decreased the thickness of monofilaments in decrements of 2 until the participant could not perceive a light touch. Next, the assessor increased monofilament thickness in increments of 1 until the participant reported a light touch, marking the final “turn around point.” The threshold was determined by averaging three of these trials. Alternatively, if the participant did not perceive a light touch with three attempts of the thickest monofilament (i.e., requiring the most force) the threshold was recorded as the maximum value (300 g). This test was repeated for each vibration condition (none, pink, white), and the averaged monofilament force was recorded as the light touch perception threshold for each condition, respectively.

### Vibration Perception

Vibration perception was determined using a Biothesiometer [Figure 1; Biothesiometer, United States, Bio-Medical Instrument Co., Newbury, OH, United States; (Bloom et al., 1984; Dyck et al., 1993; Davis et al., 1997; Jayaprakash et al., 2011)]. The Biothesiometer produces adjustable vibration via periodic displacement of a solid plastic head. Vibration perception testing was conducted following the light touch perception test using the same 4-2-1 method (Dyck et al., 1993) in supine for the tibial crest test site and lying on the intact lateral side for the head of fibula. A change in position was required because the Biothesiometer requires a perpendicular test site. This test was repeated three times and the average displacement of the Biothesiometer head was recorded as the vibration perception threshold.

### Standing Balance

Participants conducted balance tests under two standing conditions: with eyes opened and with eyes closed (Figures 1, 2). Participants stood with arms across their chest while looking at a fixation point on the wall, positioned at their preferred eye level. Participants wore their own shoes and were asked to stand with their feet as close together as possible. Center of pressure (COP) data were captured during these conditions using two floor-embedded force plates (Optima, AMTI Inc., Watertown, MA, United States). Trials with eyes open and eyes closed lasted 90 and 30 s, respectively. To avoid outlying data from initial movements, the first 4 s were trimmed, and the next 26 s from both conditions were extracted for analysis. The root-mean-square (RMS), velocity of the COP, and COP excursion were calculated and further used for non-linear time series analysis methods.

### Data Processing and Analysis

Standing data were processed using MATLAB (The MathWorks Inc., Natick, MA, United States). COP data were extracted from bilateral force plates into MATLAB 2019b, and downsampled from 600 to 100 Hz with the first 4 s of each standing trial trimmed to avoid initial outliers from sudden movements at the beginning (i.e., crossing arms). The data were smoothed using a 2nd order lowpass Butterworth filter with a 6 Hz cut off and a zero-phase digital filter determined from preliminary data using power spectral analysis (Cappozzo et al., 1995). Combining those data from both force plates, COP RMS and excursion were calculated, and velocity of the COP was calculated using the central difference method.

**FIGURE 3:**
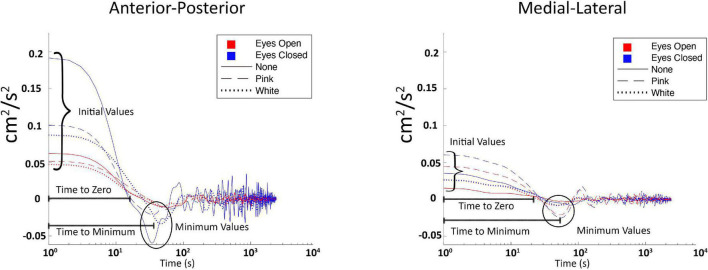
Labeled plot of center of pressure velocity autocorrelation function (COP-VAF) from one representative subject, across all three vibration conditions (white, none, and pink, respectively), and both visual conditions (eyes opened and closed).

Using COP data, two complimentary temporal based analyses were conducted to evaluate and compare the influence of vibration on stochastic characteristics over time; stabilogram diffusion analysis (SDA), and the center of pressure velocity autocorrelation function (COP-VAF; Figure 3; Collins and De Luca, 1993; Hernandez et al., 2016).

Stabilogram diffusion analysis quantifies the randomness in COP trajectory data through statistical calculations. Utilizing COP data, a measure of randomness (i.e., stochastic activity) is provided through the (i) diffusion coefficient (*D)* which represents an average measure of stochastic activity, (ii) scaling exponent (*H)*, which presents the autocorrelation of the COP (i.e., relationship between past and future positions), thus changes that move (H) in the short term between the value of 0.5 and 1 may indicate more natural or improved posture (Collins and De Luca, 1993), and (iii) the critical point which is a crossover phenomenon indicative of a transition from persistent to anti-persistent correlation regimes, or short to long term postural control strategies, based off modeling posture as a random walk (Collins and De Luca, 1993). In summary, SDA characterizes temporal measurements of stochastic activity within COP data. SDA was calculated using adapted MATLAB code from Collins and Stamp (1997).

The COP-VAF supplements SDA by providing additional insight into dynamic characteristics of postural control strategies with the allowance for a bounded COP position and validation from stochastic models that incorporated natural weight shifts (Hernandez et al., 2016). In theory, COP-VAF gives insight into postural demands and the individual response to those demands, while providing measures of stochastic activity. Hernandez et al. (2016) used the magnitude of COP velocity, combining anteroposterior with mediolateral data into a single vector, and taking the autocorrelation of these data, which in mathematical terms is:


$$C\,O\,P-V\,A\,F\,\left(\tau \right)={\left\langle v\,\left(t\right)•v\,\left(t+\tau \right)\right\rangle }_{\text{t}}$$

The average of the dot product was calculated over time scales that were applicable to human movement. We differed in their calculations only in that we did not combine anteroposterior with mediolateral data, and instead reported their respective scalar components. This allowed us to evaluate directional influences.

COP-VAF provides five measures of interest from the characteristic velocity correlation decays while standing (Hernandez et al., 2016):

**Table:** 

• Initial Value (cm^2^/s^2^):	The first point on the plot (Figure 3) is associated with the potential energy of the system. Thus, it is expected to *increase* as balance demands increase.
• Time to zero (s):	Amount of time it takes the body to arrest velocity, which is indicative of the latency in the postural response. *Decreased* time to zero has been associated with increased postural challenges.
• First minimum value (cm^2^/s^2^):	Magnitude of corrective postural response. *Increased* values have been associated with increased postural challenges.
• Time to first minimum value (s):	Amount of time needed to achieve initial postural response. *Decreased* time to first minimum implies increased postural challenges.
• Planar Diffusion Coefficient, D_0_ (cm^2^/s):	This provides a measure of the average stochastic activity that correlates to the diffusion coefficient (D) of SDA (Collins and De Luca, 1993; Hernandez et al., 2016). An *increased* value is associated with increased postural challenges.
	

To summarize the expected changes in the aforementioned variables: (a) decreases in initial value, first minimum value, and Do imply positive impacts on posture control, while (b) increases in time to zero, and time to first minimum imply positive impacts on posture control. COP-VAF shows a velocity correlation that decays over time which is represented by the aforementioned measures (initial value, time to zero, first minimum value, and time to first minimum) and depicted in Figure 3. The initial value correlates with the potential energy of the system, thus increasing as balance tasks become more challenging. Time to zero measures the time until the COP velocity reaches zero, or in other words, the time for posture response to occur, which is the instance before changing directions. First minimum value provides another measure of posture response similar to time to zero. Specifically, it measures the magnitude of response beyond the zero crossing (Hernandez et al., 2016). Regarding the diffusion coefficient, Hernandez et al. (2016) leveraged a specific case of the Green-Kubo relation because VAF decays to zero over a long time, providing an approximation of D to be calculated as:


$${\text{D}}_{0}=1/2\,\int_{0}^{\infty }{\left\langle v\,\left(t\right)•v\,\left(t+\tau \right)\right\rangle }_{\text{t}}\,\text{d}\,\tau$$

Hernandez et al. (2016) noted that integrating from 0 to 10 s gave a very close approximation of D, defining D_0_.

As we apply subthreshold vibration to the residual limb, we would expect these analyses to move toward typical standing patterns. For example, we would hypothesize the D coefficient for SDA and COP-VAF would get smaller as we apply subthreshold pink vibration implying increased stability. A notable difference between SDA and COP-VAF is SDA relies on longer data sets for accurate measures, while COP-VAF can provide similar and complementary measures with shorter datasets, which is a concern with postural trials in individuals with amputations (Doyle et al., 2008).

### Statistical Analysis

We used a series of linear mixed effect (LME) models to compare the effects of subthreshold vibration on light touch perception, vibration perception, and measures of magnitude of postural sway. Succinctly, linear mixed models consider both random and fixed effects, providing a generalization of ordinary least squares regression that side-steps issues related to repeated measures designs (e.g., non-independence) (Raudenbush and Bryk, 2002).

Model selection for each dependent variable followed the same set of analysis steps. The baseline model in each case was an intercept-only model with random participant effects. In subsequent modeling steps, we added fixed effects of vibration and vision (the focal predictors) as well as potential covariates, height, and mass, which are known to influence posture (Costello et al., 2012). Other researchers have observed inter-individual responses to noise based stimuli (Kelty-Stephen et al., 2013). For that reason, we sought to potentially contextualize such differences by inclusion of those covariates. Height and mass were added in later steps, being participant-level predictors, whereas vibration and vision were nested within each participant. In each successive model, improvement of model fit was assessed via a likelihood ratio test. Vibration and vision were retained as predictors in all higher-level models for potential implication in higher order interactions with covariates. We only report statistically significant models. Data, and further results can be seen in Supplementary Materials.

## Results

Our initial sample included 21 participants, but only 20 participants completed all trials (Figure 2); one participant was withdrawn due to dizziness.

### Light Touch and Vibration Perception

**FIGURE 4:**
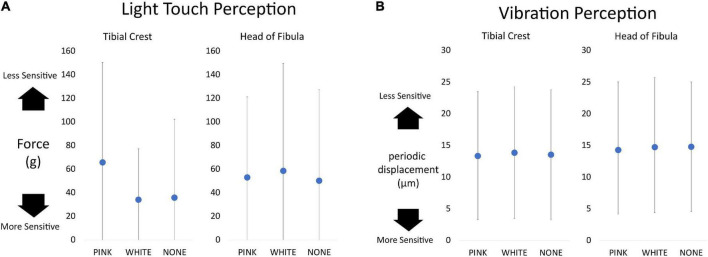
Dot plots of sensation data separated as a function of vibration condition and testing location. Overall, higher values equate to a lessened ability to detect or perceive sensation. **(A)** Light touch perception measured in grams of force required to buckle the monofilament. The maximum value a participant could receive is 300 g. **(B)** The vibration perception measured by the displacement of the Biothesiometer testing device. The highest value possible is 25 μm.

Linear mixed effect models did not reveal any effects of vibration on light touch (Figure 4A). Similar null results were obtained with respect to vibration perception (Figure 4B).

### Magnitude of Postural Sway

**FIGURE 5:**
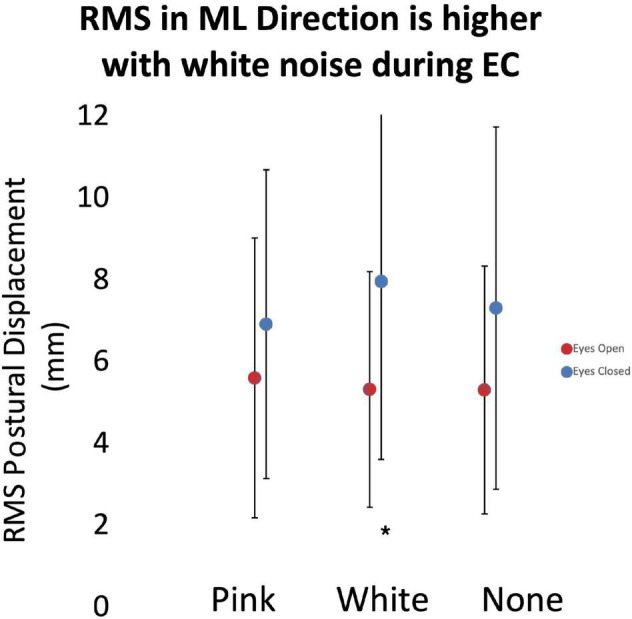
The root-mean-square (RMS) of postural displacement in the medial-lateral direction as a function of subthreshold vibration and vision. Error bars reflect 95% confidence intervals. During the eyes closed quiet standing condition, RMS displacement had a statistically significant (*) increase during white vibration conditions. Increased RMS displacement may be associated with decreased posture control mechanisms.

As noted above, we assessed the effects of subthreshold vibration on measures of the magnitude of postural sway using LME models. Analysis showed an increased RMS of the COP excursion in the mediolateral direction during the eyes closed condition (*p* = 0.027; Figure 5) with white vibration compared to that of pink vibration (13% difference) and no vibration (8.2% difference). As expected, our analysis demonstrated effects of vision (i.e., eyes opened versus eyes closed) in all relevant models.

**FIGURE 6:**
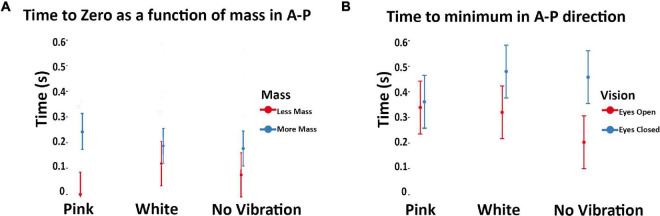
**(A)** Modeled implied mean total times to maximal compensatory response as a function of subthreshold vibration and vision in the anteroposterior direction. Error bars reflect 95% confidence intervals. Hernandez et al. (2016) demonstrated decreased values were associated with eyes closed during unipedal versus bipedal stances in healthy adults. This seems inverse for our population of unilateral amputees during eyes closed. During the eyes closed condition our values increased compared to the eyes open condition. Of note is the no vibration conditions showed a statistically significant difference during these conditions, which is altered with pink and white vibration. **(B)** Modeled implied means as a function of subthreshold vibration and body mass in the anteroposterior direction. Error bars reflect 95% confidence intervals. Note that mass was treated as a continuous variable in the underlying model. Model implied means were estimated at the extrema of the mass range in our sample to aid interpretation of interaction effects. As mean mass increases, time to zero increases with subthreshold pink vibration. The standard deviation was calculated from a non-normally distributed population using R studio. The error bar was manually cut at zero to reflect the reality that time cannot go below zero.

With respect to the impact of subthreshold vibration on the temporal structure of posture as measured by the COP-VAF, we found several reliable trends. In the no vibration condition, time to minimum in the anteroposterior direction was significantly lower with eyes open versus eyes closed (*p* = 0.045; Figure 6A). Subthreshold pink and white vibration had no statistical differences between eyes opened and eyes closed. Lastly, in our model we used mass as a covariate due to its potential impact on posture which demonstrated that as mass of the participant increases, the time to zero increases, and even more so during subthreshold pink vibration conditions (*p* = 0.017; Figure 6B).

## Discussion

The purpose of this study was to investigate the effect of subthreshold vibration on measures of somatosensation (i.e., light touch/vibration perception) and standing balance. We hypothesized that subthreshold vibration on the residual limb of individuals with a transtibial amputation would improve somatosensation and standing balance measures. Additionally, we hypothesized that subthreshold pink vibration would demonstrate the largest improvement in somatosensation and standing balance when compared to subthreshold white vibration or no vibration. Our findings partially supported our hypotheses.

### White Vibration May Amplify Root-Mean-Square of Center of Pressure in the Eyes Closed Condition

Our results indicated that white vibration increased the RMS of the COP excursion in the mediolateral direction during the eyes closed standing condition, while pink vibration had no effect (Figure 5). This effect was likely observed during eyes closed due to the increased demand on extravisual postural control mechanisms. If this finding were to support our hypotheses, we would have expected a decrease in RMS that might have been interpreted as the participants having more control over their posture. Given our results, it is possible that the white vibration may degrade perception of postural control mechanisms that rely on somatosensation, while pink vibration has little effect or a different effect. This contrasts with results by Likens et al. (2020) that demonstrated pink vibration reduced COP RMS while standing with eyes open. It may be that the effect seen with pink vibration during quiet standing is not strong enough to overcome the postural challenges of the eyes closed condition. The mechanism underpinning this finding is unclear.

### Pink and White Vibration Produce Similar Times to First Minima With Eyes Open and Eyes Closed

Both pink and white vibration produced a convergence in the eyes open and eyes closed condition for *time to minimum* when compared to no vibration (i.e., not statistically different). That no statistically significant differences were observed in eyes open versus eyes closed conditions when subthreshold vibration was applied suggest that both subthreshold pink and white vibration did influence postural control (Figure 6A). This would suggest subthreshold pink and white vibration do influence postural control, otherwise one would expect similar results to the no vibration condition given the randomized within subject study design. However, it appears that the subthreshold vibration may improve the eyes closed condition, but degrade the eyes open condition. To understand the possible influence, *time to first minimum* is when the human body has reached the maximal velocity of a compensatory postural response. In healthy populations it has been shown that decreasing *time to minimum* is associated with increased postural demands (Hernandez et al., 2016). This principle seems to be inverted for individuals with unilateral transtibial amputees given that the eyes closed condition is increased compared to the eyes open condition (Figure 6A). This change may arise from differences in basic postural mechanisms between healthy adults and individuals with unilateral amputations, such as known differences in trunk angle, COP range and excursion (Toumi et al., 2021). One possible explanation for these results is that exposure to subthreshold white and pink vibration provides additional proprioceptive information that may alter co-contraction or stiffening strategies, which are different in individuals with amputations compared to that of unimpaired adults. This in turn may interfere with an over reliance on visual feedback for posture control during eyes open, but enhance proprioceptive feedback during eyes closed. Further examination into muscle activation with white and pink vibration in unilateral transtibial amputees may provide insights into this hypothesis.

### Pink Vibration May Amplify the Interaction Between Mass and Time to Zero

Modeling of implied means (i.e., estimated means based on modeling) of mass for time to zero from COP-VAF implied an interaction between vibration and mass that was enhanced. That is, heavier participants had increased *time to zero*, an effect amplified by pink vibration (Figure 6B). Considering our previous discussion, increased mass, or inertia, may be reducing the need to produce rapid changes in posture which is further enhanced by subthreshold pink vibration. Pink vibration may enhance proprioceptive feedback allowing for greater posture control, thus amplifying the ability to reduce rapid movements in heavier individuals.

This finding highlights the important role that mass may play. To further understand this role and how *time to zero* is enhanced by pink vibration, we review prior modeling of postural control that is based upon an Ornstein–Uhlenbeck process with a harmonic restoring force that is influenced by mass (Hernandez et al., 2016). Hernandez et al. (2016) demonstrated that decreases in time to zero are associated with increased damping and stiffness. Thus, one can infer that increases in time to zero correspond with decreased damping and stiffness. It is well established that if mass is constant but stiffness decreases, our frequency of oscillation decreases. With this in mind, it is easier to understand the suggested role that mass plays with damping and stiffness, which appears to be enhanced by pink vibration. It is unclear how much changes in stiffness and mass of the amputated limb may impact these results, but mass may be an important variable for determining who may or may not benefit from subthreshold vibration with our results suggesting heavier individuals may receive a higher benefit.

### Measures of Somatosensation

Our present results showed that subthreshold vibration had no statistically significant impact on static perception of vibration or cutaneous light touch. This contrasts to other studies that demonstrate subthreshold stimulus improves tactile sensory perception (Liu et al., 2002; Collins et al., 2003; Priplata et al., 2003, 2006; Enders et al., 2013; Lipsitz et al., 2015). A notable difference between the aforementioned studies and the present results is the population studied, i.e., people with unilateral transtibial amputation. The destructive nature of amputations may decrease overall sensory perception in the residual limb. This reduction within the residual limb may require a larger difference in perception before statistical and clinical significance can be detected in a static manner (Quai et al., 2005). Additionally, interactions with the environment are experienced by more than static cutaneous touch (Carello et al., 2008; Carello and Turvey, 2017). Namingly, *dynamic touch* is an independent domain with a central role in postural control because it provides additional proprioceptive information (Carello et al., 2008; Carello and Turvey, 2017). Subthreshold vibration did not appear to influence static cutaneous touch, however, it may influence *dynamic touch*, which plays an important function in posture.

### Study Limitations

Several limitations exist within our study. The small sample size reduces the power of the study. Additionally, we had many confounders within our population. Some of these include features of amputations such as time since amputation, cause of amputation, and underlying pathologies and comorbid conditions (e.g., diabetic neuropathy). Each participant wore their preferred shoe and prosthetic device which introduced more heterogeneity into an already diverse population, potentially impacting the results. Lastly, our sample demographics were highly skewed toward older, white males, which hinders the generalizability of the results to other demographics.

The pathology of our study population and the specific structures of interest (tibial crest and head of fibula) were critical aspects of evaluating the influence of subthreshold vibration. The locations we evaluated may have been too remote from the vibrating device, which was placed proximally to the prosthesis midway on the thigh (Figure 1A). Enders et al. (2013) applied vibration proximally to the region of interest and found stroke survivors had an immediate increase in light touch perception at the distal region of interest. Our approach of applying vibration proximal to the tibial crest and head of fibula was similar to Enders et al. (2013) proximal approach. A closer placement, or intra-prosthetic placement may elicit stronger responses. It should be noted that other types of subthreshold stimulus (e.g., electrical) have been successful in improving balance measures in individuals with amputations (Lee et al., 2007).

Physiological reasons that resulted in a mixed response to subthreshold vibratory stimulus may include neurological differences in mechanoreceptors or their heterogeneous distribution throughout the body (Ackerley et al., 2014; S Costanzo, 2019). The heterogeneous distribution may have confounded with our small sample size, providing overall less sensate structure and less of a response to the intervention. The study population was limited to individuals who were comfortable with daily walking and balance challenges, but it is possible less able individuals may benefit differently from subthreshold vibration.

Our within subject design offers both advantages and limitations. One limitation includes the need for a washout period. We chose to include a 5 min washout period between vibration conditions due to previous literature demonstrating instantaneous and short lived results. It is plausible that a 5 min washout period is not sufficiently long to prevent carry over effects from the previous condition. Additional limitations include patient fatigue, and learning effect.

While previous studies showed a wide range (50–90%) of subthreshold stimulation could improve the outcomes of interest (Liu et al., 2002; Collins et al., 2003; Priplata et al., 2003, 2006; Enders et al., 2013; Lipsitz et al., 2015) this may not be true for the structures of the residual limb. The hardware used in our design was only capable of large, discrete increments that provided varying subthreshold values between 50 and 90%. The residual limb may have a narrow window of “optimal noise” necessary for the principle of SR to achieve peak performance.

To summarize the limitations and potential avenues for future research, one could investigate intra-prosthetic vibratory stimulus placed on the head of the fibula, or the tibial crest of the residual limb. Initially we tried providing a vibratory stimulus to the entire prosthetic socket, but the socket influenced the signal, as we were unable to achieve the desired signals (pink and white) at the residual limb. If one could achieve this it may better reflect the success seen in vibratory insoles to reduce peripheral neuropathy (Lipsitz et al., 2015).

## Conclusion

In the present study, subthreshold pink vibrotactile noise positively influenced the temporal structure of standing balance compared to no vibration and white vibration. Interestingly white vibration displayed mixed ability to influence standing balance. However, there was no influence on measures of somatosensation. The underlying reasons for these results are likely multifactorial and are not fully understood at this time.

While our hypothesis was only partially supported due to the lack of influence on somatosensation, it remains that subthreshold pink and white vibration influences *time to minimum* and amplifies the natural interaction between mass and *time to zero.* This means it is possible that subthreshold vibration could improve balance in individuals with unilateral transtibial amputations, and mass may be a natural indicator for response. These influences of stochastic resonance on the temporal aspects of standing balance establish novel findings that may promote future developments. Additionally, it has become evident that various types of noise may be more beneficial for certain populations and activities.

In summary, applying pink or white vibration via stochastic resonance may be a feasible intervention for individuals with lower limb amputations, but is in its infancy stage and needs more research. A larger sample size, and slight alterations to the intervention may reveal larger effects in somatosensation, and standing balance. This study is complementary to existing stochastic resonance literature, and furthers foundational work to utilize stochastic resonance as a rehabilitation tool.

## Data Availability Statement

The original contributions presented in the study are included in the article/Supplementary Material, further inquiries can be directed to the corresponding author.

## Ethics Statement

The studies involving human participants were reviewed and approved by the University of Nebraska Medical Center Institutional Review Board and the VA Nebraska-Western Iowa Health Care System Institutional Review Board. The patients/participants provided their written informed consent to participate in this study.

## Author Contributions

ZM contributed to methodology development, equipment development, data collection, subject recruitment, data analysis, preliminary statistical analysis, writing the original manuscript, revisions, and visualizations. AL contributed to writing the original draft, revising, and editing the manuscript, and statistical analysis. JK contributed to the methodology development, data collection, and revising and editing the manuscript. KT contributed to the methodology development and revising and editing the manuscript. SW, AJ, and NS contributed to conceptualization of the study, methodology development, and revising and editing the manuscript. MH contributed to data analysis and algorithm development, writing the original manuscript, revising, and editing. All authors contributed to the article and approved the submitted version.

## Conflict of Interest

The authors declare that the research was conducted in the absence of any commercial or financial relationships that could be construed as a potential conflict of interest.

## Publisher’s Note

All claims expressed in this article are solely those of the authors and do not necessarily represent those of their affiliated organizations, or those of the publisher, the editors and the reviewers. Any product that may be evaluated in this article, or claim that may be made by its manufacturer, is not guaranteed or endorsed by the publisher.
