# Supplementary material for: Subthreshold Vibration Influences Standing Balance but Has Unclear Impact on Somatosensation in Persons With Transtibial Amputations
Source: Front Physiol. 2022 Feb 2;13:810079. doi: 10.3389/fphys.2022.810079 (PMC8847287; doi:10.3389/fphys.2022.810079)
Supplement: Supplementary file 2 [file Data_Sheet_1.PDF]

basic_models_x_no_filter.R

alikens

2020-07-02

```r
library(lmerTest)
```

```
## Loading required package: lme4

## Loading required package: Matrix

##
## Attaching package: 'lmerTest'

## The following object is masked from 'package:lme4':
##
##     lmer

## The following object is masked from 'package:stats':
##
##     step
```

```r
library(ggplot2)
library(sjPlot)
```

```
## Install package "strengejacke" from GitHub (`devtools::install_github("strengejacke/strengejacke")`)
```

```r
# look at differences in threshold
# this may need to use different distributions
# predict clusters in perception variables from the type of amputaion, age, weight

# read in the data
sense = read.csv("./data/Sensation_Data_Reorganized.csv")
copv = read.delim("./data/Amputee_COP_VAC_100HZ_summary_noFilter.txt")
# copv = copv[,!grepl('H', names(copv))]
join_sense = c('subj', 'Vibration', 'CondNum')
join_copv = c('subj', 'Vibration', 'CondNum')
copv = merge(copv,sense, by.x = join_copv, by.y = join_sense)
copv$Vision = as.factor(copv$Vision)
copv$Vibration = as.factor(copv$Vibration)
copv = copv[copv$Vision != 0,]
copv$Cause = factor(copv$Cause)
copv$TCThresholdGroup = factor(copv$TCThresholdGroup)
copv$Mass2 = copv$Mass^2
zMass = as.numeric(scale(copv$Mass))
copv$zMass = zMass
copv$zMass2 = I(zMass^2)

# model for zeroTime
m0 = lmerTest::lmer(zeroTimeX ~ 1 + (1|subj), data = copv)
m1 = lmerTest::lmer(zeroTimeX ~ 1 + Vibration + (1|subj), data = copv)
```

```r
m2 = lmerTest::lmer(zeroTimeX ~ 1 + Vibration + Vision + (1|subj), data = copv)
# m3 = lmerTest::lmer(zeroTimeX ~ 1 + Vibration * Vision + (1|subj), data = copv)
# m4 = lmerTest::lmer(zeroTimeX ~ 1 + Cause + Vision * Vibration + (1|subj), data = copv)
# m4 = lmerTest::lmer(zeroTimeX ~ 1 + TCThresholdGroup + Vision * Vibration + (1|subj), data = copv)
# m4 = lmerTest::lmer(zeroTimeX ~ 1 + Vibration*Vision + Mass + (1|subj), data = copv)
# m5 = lmerTest::lmer(zeroTimeX ~ 1 + Vibration * Vision + Mass + Mass:Vibration + (1|subj), data = cop
#m6 = lmerTest::lmer(zeroTimeX ~ 1 + Mass * Vision * Vibration + (1|subj), data = copv)

anova(m0, m1, m2)
```

```
## refitting model(s) with ML (instead of REML)
```

```
## Data: copv
## Models:
## m0: zeroTimeX ~ 1 + (1 | subj)
## m1: zeroTimeX ~ 1 + Vibration + (1 | subj)
## m2: zeroTimeX ~ 1 + Vibration + Vision + (1 | subj)
##    npar     AIC     BIC logLik deviance  Chisq Df Pr(>Chisq)
## m0    3 -267.00 -258.80 136.50  -273.00
## m1    5 -265.57 -251.88 137.78  -275.57 2.5621  2    0.27775
## m2    6 -270.58 -254.16 141.29  -282.58 7.0134  1    0.00809 **
## ---
## Signif. codes:  0 '***' 0.001 '**' 0.01 '*' 0.05 '.' 0.1 ' ' 1
```

```r
print(summary(m2))
```

```
## Linear mixed model fit by REML. t-tests use Satterthwaite's method [
## lmerModLmerTest]
## Formula: zeroTimeX ~ 1 + Vibration + Vision + (1 | subj)
##    Data: copv
##
## REML criterion at convergence: -255
##
## Scaled residuals:
##     Min      1Q  Median      3Q     Max
## -2.6211 -0.6030 -0.1502  0.4188  3.4781
##
## Random effects:
##  Groups   Name        Variance Std.Dev.
##  subj     (Intercept) 0.002607 0.05106
##  Residual             0.003888 0.06235
## Number of obs: 114, groups:  subj, 19
##
## Fixed effects:
##               Estimate Std. Error         df t value Pr(>|t|)
## (Intercept)  0.2326298  0.0165411 42.9276555  14.064  < 2e-16 ***
## Vibration2  -0.0001518  0.0143047 92.0000003  -0.011  0.99156
## Vibration3   0.0203084  0.0143047 92.0000003   1.420  0.15907
## Vision2     -0.0310096  0.0116798 92.0000003  -2.655  0.00935 **
## ---
## Signif. codes:  0 '***' 0.001 '**' 0.01 '*' 0.05 '.' 0.1 ' ' 1
##
## Correlation of Fixed Effects:
##            (Intr) Vbrtn2 Vbrtn3
## Vibration2 -0.432
```

```
## Vibration3 -0.432  0.500
## Vision2    -0.353  0.000  0.000
```

```r
# visualize Vision by Vibration Interaction
g1 = ggplot(copv, aes(Vision, zeroTimeX, colour = Vision, fill = Vision)) +
  stat_summary(fun.data = 'mean_cl_boot', position = position_dodge(),geom = 'bar') +
  stat_summary(fun.data = 'mean_cl_boot', position = position_dodge(0.9), geom = 'errorbar', colour = 'l
print(g1)
```

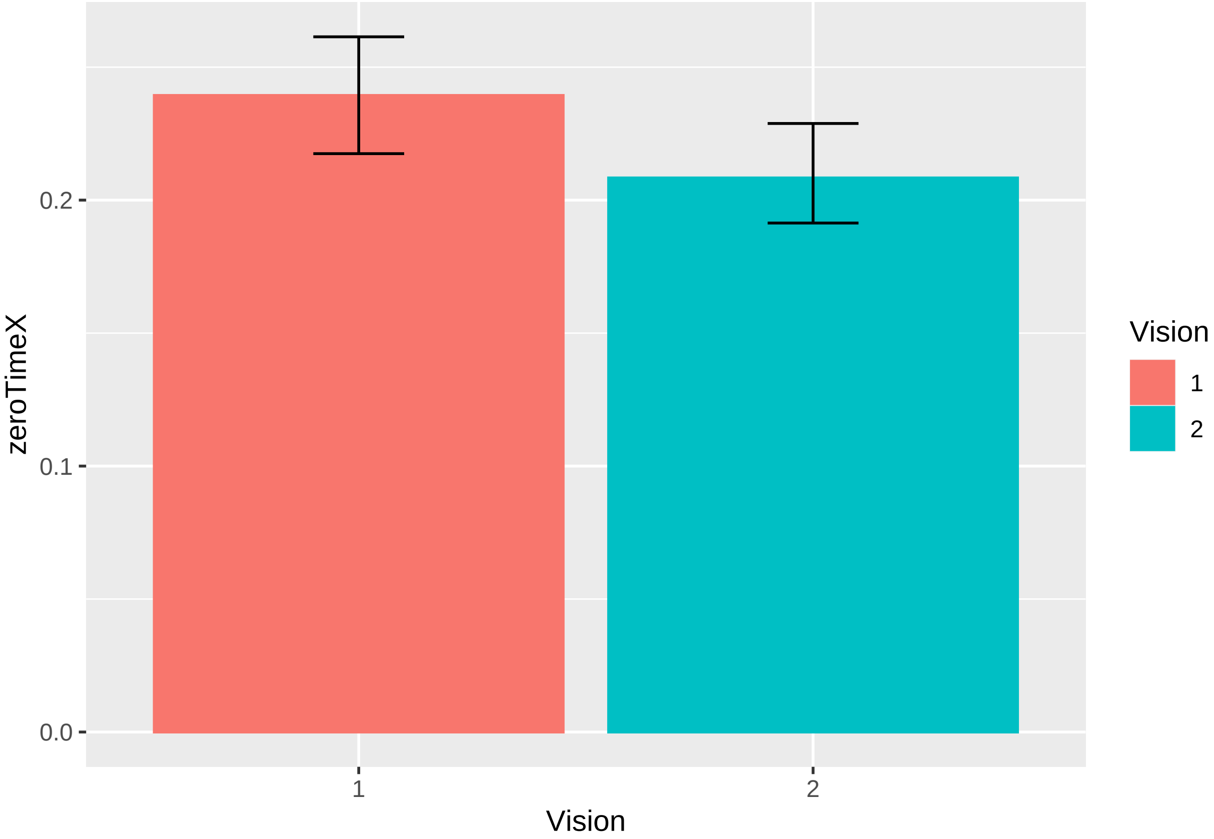


```r
# model for minTimeX
m0 = lmerTest::lmer(minTimeX ~ 1 + (1|subj), data = copv)
m1 = lmerTest::lmer(minTimeX ~ 1 + Vibration + (1|subj), data = copv)
m2 = lmerTest::lmer(minTimeX ~ 1 + Vibration + Vision + (1|subj), data = copv)
# m3 = lmerTest::lmer(minTimeX ~ 1 + Vibration * Vision + (1|subj), data = copv)
# m4 = lmerTest::lmer(minTimeX ~ 1 + Cause + Vision * Vibration + (1|subj), data = copv)
# m4 = lmerTest::lmer(minTimeX ~ 1 + TCThresholdGroup +  + Vision * Vibration + (1|subj), data = copv)
# m4 = lmerTest::lmer(minTimeX ~ 1 + Vibration*Vision + Mass + (1|subj), data = copv)
# m5 = lmerTest::lmer(minTimeX ~ 1 + Vibration * Vision + Mass + Mass:Vibration + (1|subj), data = copv
# m6 = lmerTest::lmer(minTimeX ~ 1 + Mass * Vision * Vibration + (1|subj), data = copv)

anova(m0, m1, m2)
```

```
## refitting model(s) with ML (instead of REML)

## Data: copv
## Models:
## m0: minTimeX ~ 1 + (1 | subj)
## m1: minTimeX ~ 1 + Vibration + (1 | subj)
## m2: minTimeX ~ 1 + Vibration + Vision + (1 | subj)
```

```
##    npar      AIC     BIC  logLik deviance  Chisq Df Pr(>Chisq)
## m0    3  -9.5625 -1.3539  7.7812  -15.562
## m1    5  -7.1304  6.5506  8.5652  -17.130 1.5679  2    0.45660
## m2    6 -10.5178  5.8994 11.2589  -22.518 5.3874  1    0.02028 *
## ---
## Signif. codes:  0 '***' 0.001 '**' 0.01 '*' 0.05 '.' 0.1 ' ' 1
```

```
print(summary(m2))
```

```
## Linear mixed model fit by REML. t-tests use Satterthwaite's method [
## lmerModLmerTest]
## Formula: minTimeX ~ 1 + Vibration + Vision + (1 | subj)
##    Data: copv
##
## REML criterion at convergence: -3.8
##
## Scaled residuals:
##     Min      1Q  Median      3Q     Max
## -1.6246 -0.6094 -0.1958  0.4958  4.5197
##
## Random effects:
##  Groups   Name        Variance Std.Dev.
##  subj     (Intercept) 0.01087  0.1043
##  Residual             0.04267  0.2066
## Number of obs: 114, groups:  subj, 19
##
## Fixed effects:
##               Estimate Std. Error         df t value Pr(>|t|)
## (Intercept)  0.5079825  0.0454904 67.4649576  11.167   <2e-16 ***
## Vibration2   0.0007895  0.0473895 92.0000001   0.017   0.9867
## Vibration3   0.0526316  0.0473895 92.0000001   1.111   0.2696
## Vision2     -0.0896491  0.0386933 92.0000001  -2.317   0.0227 *
## ---
## Signif. codes:  0 '***' 0.001 '**' 0.01 '*' 0.05 '.' 0.1 ' ' 1
##
## Correlation of Fixed Effects:
##            (Intr) Vbrtn2 Vbrtn3
## Vibration2 -0.521
## Vibration3 -0.521  0.500
## Vision2    -0.425  0.000  0.000
```

```
g2 = ggplot(copv, aes(Vision, minTimeX, colour = Vision, fill = Vision)) +
  stat_summary(fun.data = 'mean_cl_boot', position = position_dodge(),geom = 'bar') +
  stat_summary(fun.data = 'mean_cl_boot', position = position_dodge(0.9), geom = 'errorbar', colour = 'l
print(g2)
```

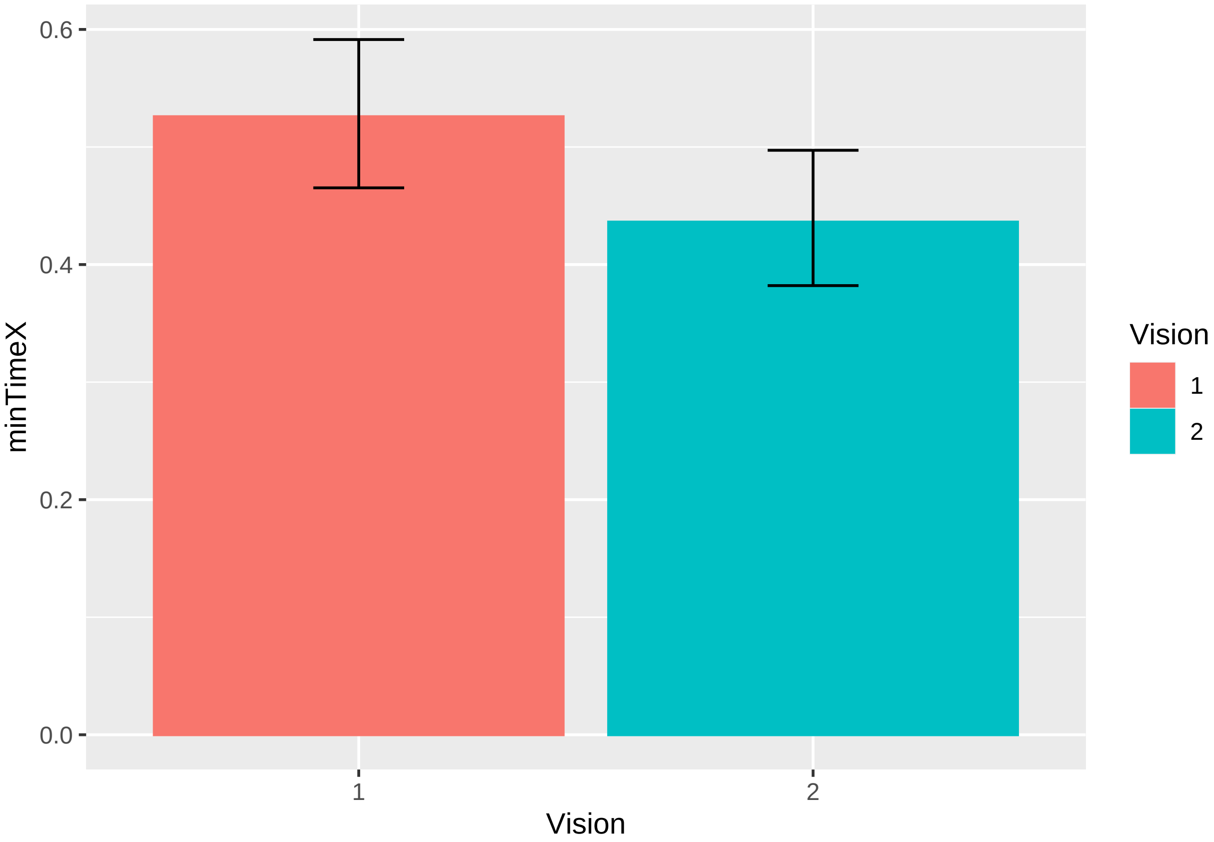


```
# model for minValX
m0 = lmerTest::lmer(minValX ~ 1 + (1|subj), data = copv)
m1 = lmerTest::lmer(minValX ~ 1 + Vibration + (1|subj), data = copv)
m2 = lmerTest::lmer(minValX ~ 1 + Vibration + Vision + (1|subj), data = copv)
m3 = lmerTest::lmer(minValX ~ 1 + Vibration * Vision + (1|subj), data = copv)
m4 = lmerTest::lmer(minValX ~ 1 + Cause + Vision * Vibration + (1|subj), data = copv)
# m4 = lmerTest::lmer(minValX ~ 1 + TCThresholdGroup +  + Vision * Vibration + (1|subj), data = copv)
# m4 = lmerTest::lmer(minValX ~ 1 + Vibration*Vision + Mass + (1|subj), data = copv)
# m5 = lmerTest::lmer(minValX ~ 1 + Vibration * Vision + Cause + Cause:Vibration + (1|subj), data = cop
m5 = lmerTest::lmer(minValX ~ 1 + Vibration * Vision + Cause + Cause:Vision + (1|subj), data = copv)


anova(m0, m1, m2, m3, m4, m5)
```

```
## refitting model(s) with ML (instead of REML)
```

```
## Data: copv
## Models:
## m0: minValX ~ 1 + (1 | subj)
## m1: minValX ~ 1 + Vibration + (1 | subj)
## m2: minValX ~ 1 + Vibration + Vision + (1 | subj)
## m3: minValX ~ 1 + Vibration * Vision + (1 | subj)
## m4: minValX ~ 1 + Cause + Vision * Vibration + (1 | subj)
## m5: minValX ~ 1 + Vibration * Vision + Cause + Cause:Vision + (1 |
## m5:     subj)
##    npar     AIC     BIC logLik deviance   Chisq Df Pr(>Chisq)
## m0    3 -1462.0 -1453.7 733.97  -1468.0
## m1    5 -1458.5 -1444.8 734.26  -1468.5  0.5679  2   0.752821
```

```
## m2    6 -1477.3 -1460.9 744.65  -1489.3 20.7789  1  5.155e-06 ***
## m3    8 -1473.8 -1451.9 744.92  -1489.8  0.5396  2   0.763526
## m4   10 -1474.5 -1447.1 747.23  -1494.5  4.6311  2   0.098713 .
## m5   12 -1481.0 -1448.1 752.48  -1505.0 10.5040  2   0.005237 **
## ---
## Signif. codes:  0 '***' 0.001 '**' 0.01 '*' 0.05 '.' 0.1 ' ' 1
```

```r
print(summary(m5))
```

```
## Linear mixed model fit by REML. t-tests use Satterthwaite's method [
## lmerModLmerTest]
## Formula: minValX ~ 1 + Vibration * Vision + Cause + Cause:Vision + (1 |
##     subj)
##    Data: copv
##
## REML criterion at convergence: -1338.8
##
## Scaled residuals:
##     Min      1Q  Median      3Q     Max
## -4.9015 -0.2784  0.0257  0.2755  2.0124
##
## Random effects:
##  Groups   Name        Variance  Std.Dev.
##  subj     (Intercept) 8.921e-08 0.0002987
##  Residual             8.524e-08 0.0002920
## Number of obs: 114, groups:  subj, 19
##
## Fixed effects:
##                        Estimate Std. Error         df t value Pr(>|t|)
## (Intercept)          -3.767e-05  1.407e-04  2.813e+01  -0.268  0.79086
## Vibration2           -6.031e-06  9.473e-05  8.800e+01  -0.064  0.94938
## Vibration3           -3.502e-05  9.473e-05  8.800e+01  -0.370  0.71249
## Vision2              -9.551e-05  1.187e-04  8.800e+01  -0.804  0.42336
## Cause2               -8.871e-05  2.008e-04  2.063e+01  -0.442  0.66329
## Cause3               -1.681e-04  1.833e-04  2.063e+01  -0.917  0.36968
## Vibration2:Vision2 -9.503e-05  1.340e-04  8.800e+01  -0.709  0.47996
## Vibration3:Vision2 -1.990e-05  1.340e-04  8.800e+01  -0.149  0.88223
## Vision2:Cause2     -2.532e-06  1.396e-04  8.800e+01  -0.018  0.98557
## Vision2:Cause3     -3.647e-04  1.274e-04  8.800e+01  -2.862  0.00526 **
## ---
## Signif. codes:  0 '***' 0.001 '**' 0.01 '*' 0.05 '.' 0.1 ' ' 1
##
## Correlation of Fixed Effects:
##             (Intr) Vbrtn2 Vbrtn3 Visin2 Cause2 Cause3 Vb2:V2 Vb3:V2 Vs2:C2
## Vibration2  -0.337
## Vibration3  -0.337  0.500
## Vision2     -0.422  0.399  0.399
## Cause2      -0.595  0.000  0.000  0.170
## Cause3      -0.651  0.000  0.000  0.186  0.456
## Vbrtn2:Vsn2  0.238 -0.707 -0.354 -0.564  0.000  0.000
## Vbrtn3:Vsn2  0.238 -0.354 -0.707 -0.564  0.000  0.000  0.500
## Vision2:Cs2  0.207  0.000  0.000 -0.490 -0.348 -0.159  0.000  0.000
## Vision2:Cs3  0.226  0.000  0.000 -0.537 -0.159 -0.348  0.000  0.000  0.456
```

```r
g3 = ggplot(copv, aes(Cause, minValX, colour = Vision, fill = Vision)) +
  stat_summary(fun.data = 'mean_cl_boot', position = position_dodge(),geom = 'bar') +
  stat_summary(fun.data = 'mean_cl_boot', position = position_dodge(0.9), geom = 'errorbar', colour = 'l

print(g3)
```

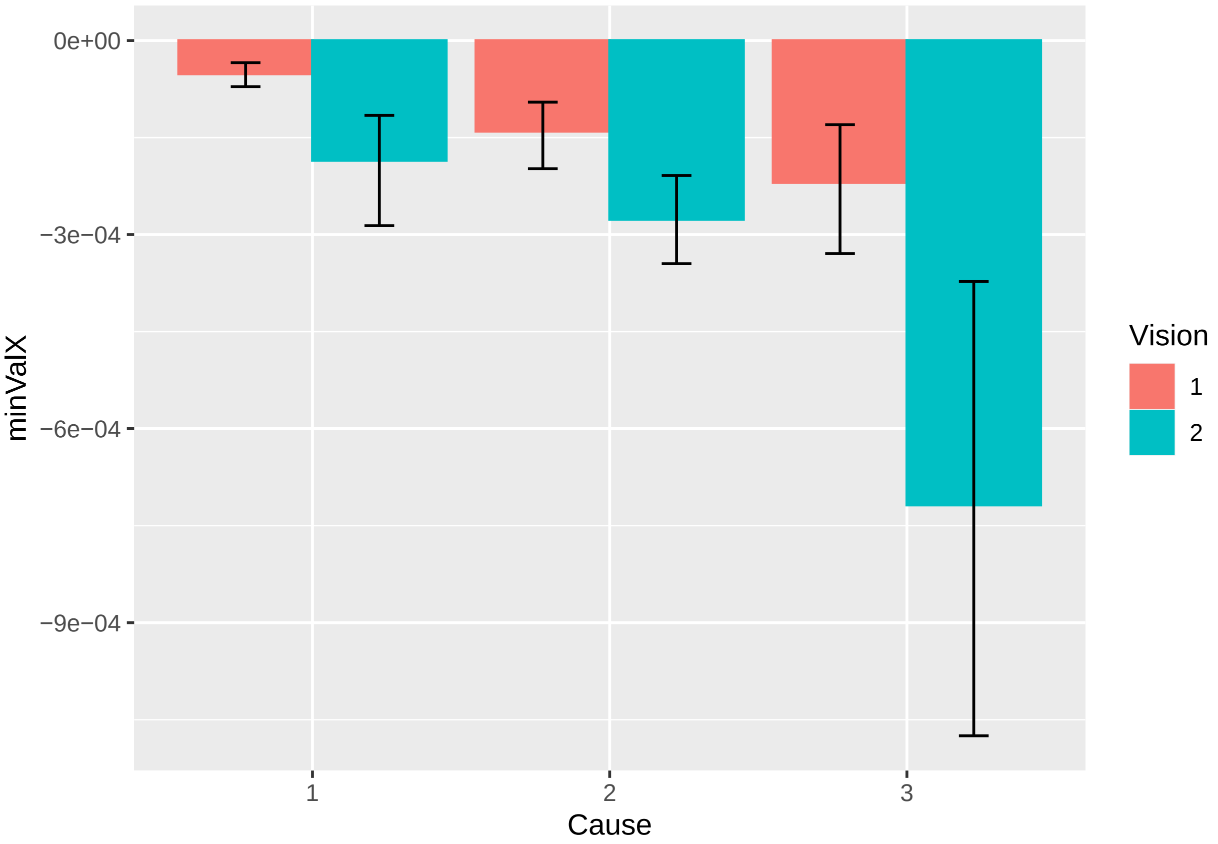


```r
# model for CvOX
m0 = lmerTest::lmer(CvOX ~ 1 + (1|subj), data = copv)
m1 = lmerTest::lmer(CvOX ~ 1 + Vibration + (1|subj), data = copv)
m2 = lmerTest::lmer(CvOX ~ 1 + Vibration + Vision + (1|subj), data = copv)
# m3 = lmerTest::lmer(CvOX ~ 1 + Vibration  + Vision + (1|subj), data = copv)
m3 = lmerTest::lmer(CvOX ~ 1 + Cause + Vision  + Vibration + (1|subj), data = copv)
# m4 = lmerTest::lmer(CvOX ~ 1 + TCThresholdGroup +  + Vision * Vibration + (1|subj), data = copv)
# m4 = lmerTest::lmer(CvOX ~ 1 + Vibration*Vision + Mass + (1|subj), data = copv)
m4 = lmerTest::lmer(CvOX ~ 1 + Vibration * Vision + Cause + Cause:Vision + (1|subj), data = copv)
#m6 = lmerTest::lmer(CvOX ~ 1 + Mass * Vision * Vibration + (1|subj), data = copv)

anova(m0, m1, m2, m3, m4)
```

```
## refitting model(s) with ML (instead of REML)

## Data: copv
## Models:
## m0: CvOX ~ 1 + (1 | subj)
## m1: CvOX ~ 1 + Vibration + (1 | subj)
## m2: CvOX ~ 1 + Vibration + Vision + (1 | subj)
## m3: CvOX ~ 1 + Cause + Vision + Vibration + (1 | subj)
## m4: CvOX ~ 1 + Vibration * Vision + Cause + Cause:Vision + (1 | subj)
```

```
##    npar     AIC     BIC logLik deviance   Chisq Df Pr(>Chisq)
## m0    3 -1118.7 -1110.5 562.34  -1124.7
## m1    5 -1116.1 -1102.5 563.06  -1126.1  1.4477  2    0.48487
## m2    6 -1135.2 -1118.8 573.58  -1147.2 21.0414  1  4.495e-06 ***
## m3    8 -1135.9 -1114.0 575.97  -1151.9  4.7664  2    0.09225 .
## m4   12 -1140.8 -1108.0 582.43  -1164.8 12.9172  4    0.01169 *
## ---
## Signif. codes:  0 '***' 0.001 '**' 0.01 '*' 0.05 '.' 0.1 ' ' 1
```

```r
print(summary(m4))
```

```
## Linear mixed model fit by REML. t-tests use Satterthwaite's method [
## lmerModLmerTest]
## Formula: CvOX ~ 1 + Vibration * Vision + Cause + Cause:Vision + (1 | subj)
##    Data: copv
##
## REML criterion at convergence: -1028.8
##
## Scaled residuals:
##     Min      1Q  Median      3Q     Max
## -2.1140 -0.3328 -0.0030  0.2341  5.8642
##
## Random effects:
##  Groups   Name        Variance  Std.Dev.
##  subj     (Intercept) 2.128e-06 0.001459
##  Residual             1.631e-06 0.001277
## Number of obs: 114, groups:  subj, 19
##
## Fixed effects:
##                      Estimate Std. Error        df t value Pr(>|t|)
## (Intercept)         1.885e-04  6.625e-04 2.585e+01   0.285  0.77826
## Vibration2          1.467e-04  4.143e-04 8.800e+01   0.354  0.72414
## Vibration3          1.594e-04  4.143e-04 8.800e+01   0.385  0.70136
## Vision2             2.794e-04  5.194e-04 8.800e+01   0.538  0.59194
## Cause2              3.483e-04  9.571e-04 1.978e+01   0.364  0.71978
## Cause3              8.250e-04  8.737e-04 1.978e+01   0.944  0.35642
## Vibration2:Vision2 5.013e-04  5.859e-04 8.800e+01   0.856  0.39453
## Vibration3:Vision2 2.327e-04  5.859e-04 8.800e+01   0.397  0.69215
## Vision2:Cause2     8.952e-05  6.105e-04 8.800e+01   0.147  0.88376
## Vision2:Cause3     1.760e-03  5.573e-04 8.800e+01   3.157  0.00218 **
## ---
## Signif. codes:  0 '***' 0.001 '**' 0.01 '*' 0.05 '.' 0.1 ' ' 1
##
## Correlation of Fixed Effects:
##             (Intr) Vbrtn2 Vbrtn3 Visin2 Cause2 Cause3 Vb2:V2 Vb3:V2 Vs2:C2
## Vibration2  -0.313
## Vibration3  -0.313  0.500
## Vision2     -0.392  0.399  0.399
## Cause2      -0.602  0.000  0.000  0.156
## Cause3      -0.659  0.000  0.000  0.171  0.456
## Vbrtn2:Vsn2  0.221 -0.707 -0.354 -0.564  0.000  0.000
## Vbrtn3:Vsn2  0.221 -0.354 -0.707 -0.564  0.000  0.000  0.500
## Vision2:Cs2  0.192  0.000  0.000 -0.490 -0.319 -0.146  0.000  0.000
## Vision2:Cs3  0.210  0.000  0.000 -0.537 -0.146 -0.319  0.000  0.000  0.456
```

```
g4 = ggplot(copv, aes(Cause, Cv0X, colour = Vision, fill = Vision)) +
  stat_summary(fun.data = 'mean_cl_boot', position = position_dodge(),geom = 'bar') +
  stat_summary(fun.data = 'mean_cl_boot', position = position_dodge(0.9), geom = 'errorbar', colour = 'l
print(g4)
```

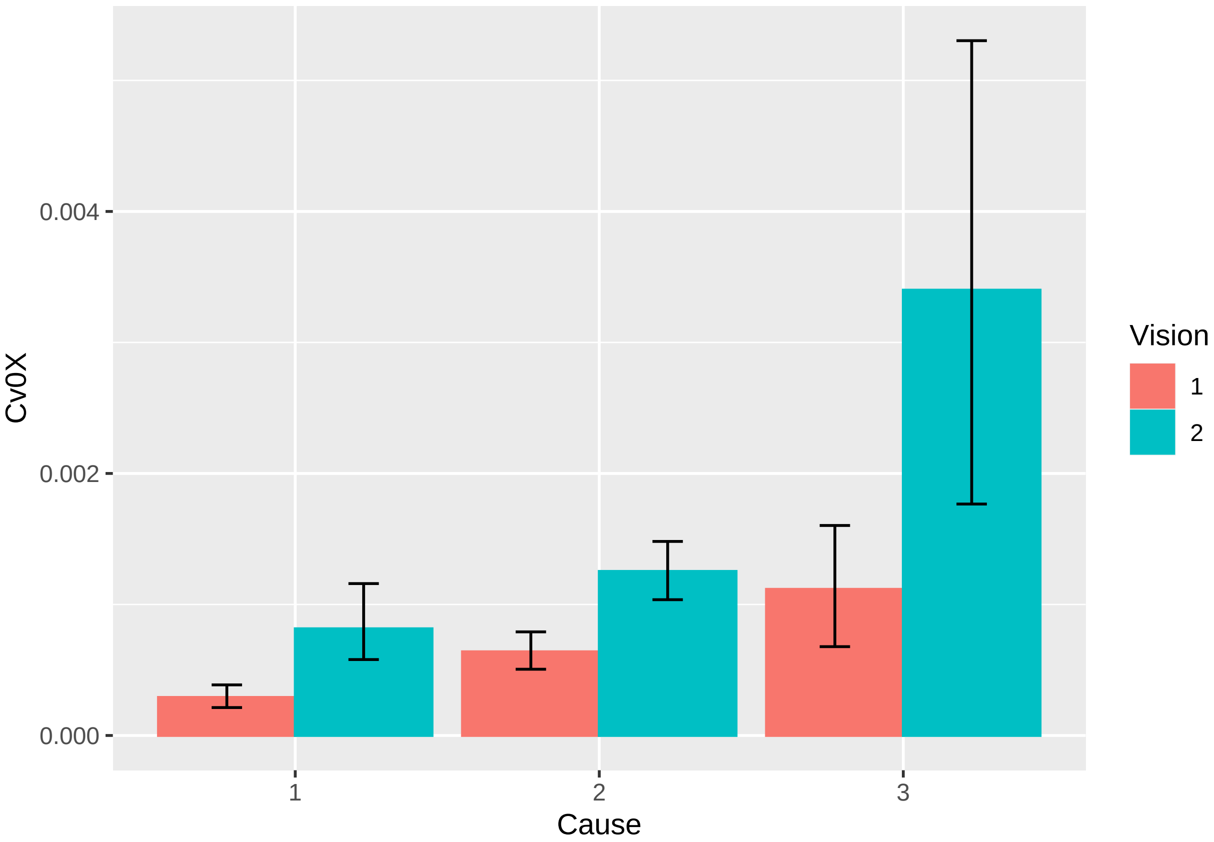


```
# model for diffusion coefficient - singular fit
# m0 = lmerTest::lmer(DiffusionCoefX ~ 1 + ( 1|subj ), data = copv)
# m1 = lmerTest::lmer(DiffusionCoefX ~ 1 + Vision + (1|subj), data = copv)
# m2 = lmerTest::lmer(DiffusionCoefX ~ 1 + Vision + Vibration + (1|subj), data = copv)
# m3 = lmerTest::lmer(DiffusionCoefX ~ 1 + Vision * Vibration + (1|subj), data = copv)
# anova(m0, m1, m2, m3)


g5 = ggplot(copv, aes(Vibration, DiffusionCoefX, colour = Vision, fill = Vision)) +
  stat_summary(fun.data = 'mean_cl_boot', position = position_dodge(),geom = 'bar') +
  stat_summary(fun.data = 'mean_cl_boot', position = position_dodge(0.9), geom = 'errorbar', colour = 'l
print(g5)
```

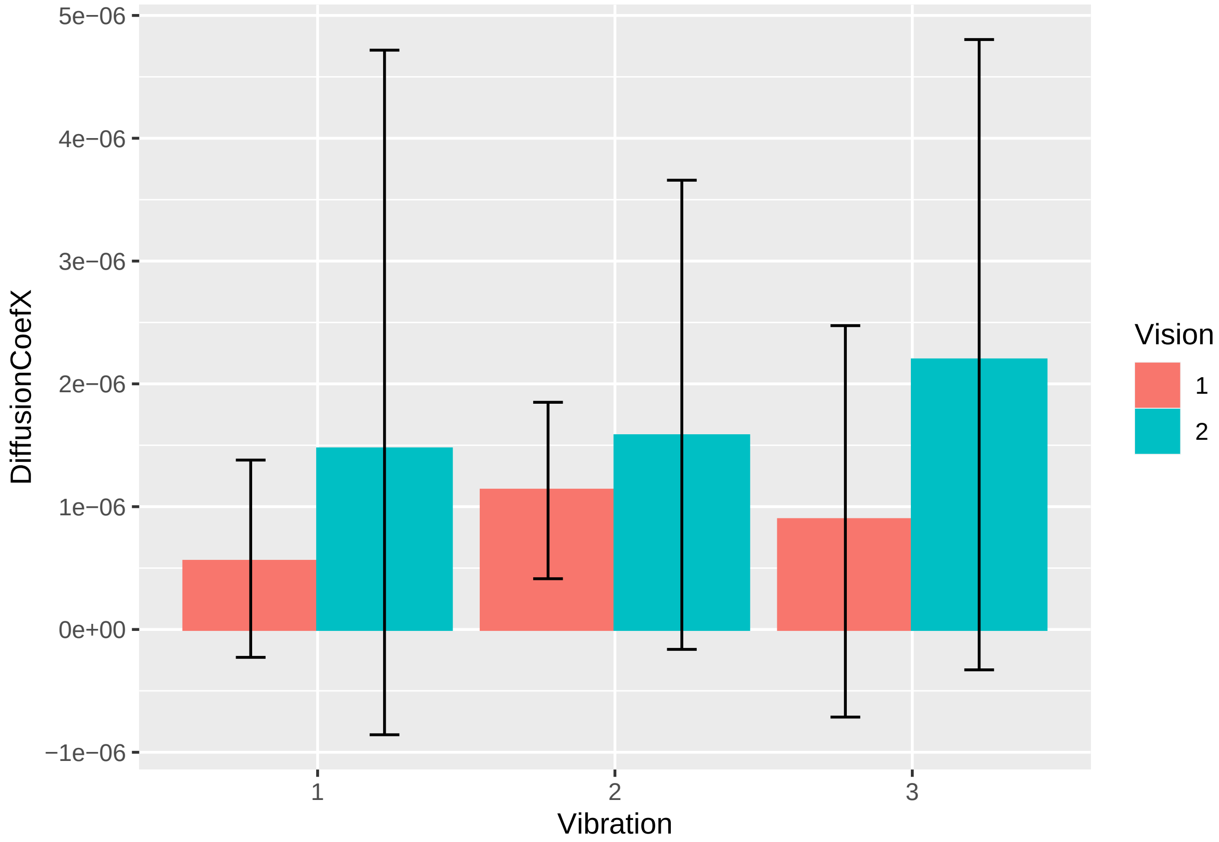


```r
# model for AMMVx
m0 = lmerTest::lmer(AMMVx ~ 1 + (1|subj), data = copv)
m1 = lmerTest::lmer(AMMVx ~ 1 + Vibration + (1|subj), data = copv)
m2 = lmerTest::lmer(AMMVx ~ 1 + Vibration + Vision + (1|subj), data = copv)
# m3 = lmerTest::lmer(AMMVx ~ 1 + Vibration * Vision + (1|subj), data = copv)
# m3 = lmerTest::lmer(AMMVx ~ 1 + Cause + Vision + Vibration + (1|subj), data = copv)
# m3 = lmerTest::lmer(AMMVx ~ 1 + TCThresholdGroup + Vision + Vibration + (1|subj), data = copv)
# m3 = lmerTest::lmer(AMMVx ~ 1 + Vibration +Vision + Mass + (1|subj), data = copv)
# m5 = lmerTest::lmer(AMMVx ~ 1 + Vibration * Vision + Mass + Mass:Vibration + (1|subj), data = copv)
#m6 = lmerTest::lmer(AMMVx ~ 1 + Mass * Vision * Vibration + (1|subj), data = copv)

anova(m0, m1, m2)
```

```
## refitting model(s) with ML (instead of REML)
```

```
## Data: copv
## Models:
## m0: AMMVx ~ 1 + (1 | subj)
## m1: AMMVx ~ 1 + Vibration + (1 | subj)
## m2: AMMVx ~ 1 + Vibration + Vision + (1 | subj)
##    npar     AIC     BIC logLik deviance   Chisq Df Pr(>Chisq)
## m0    3 -429.88 -421.67 217.94  -435.88
## m1    5 -427.46 -413.78 218.73  -437.46  1.5791  2      0.454
## m2    6 -480.00 -463.59 246.00  -492.00 54.5449  1  1.519e-13 ***
## ---
## Signif. codes:  0 '***' 0.001 '**' 0.01 '*' 0.05 '.' 0.1 ' ' 1
```

```r
print(summary(m2))
```

```
## Linear mixed model fit by REML. t-tests use Satterthwaite's method [
## lmerModLmerTest]
## Formula: AMMVx ~ 1 + Vibration + Vision + (1 | subj)
##    Data: copv
##
## REML criterion at convergence: -457.6
##
## Scaled residuals:
##     Min      1Q  Median      3Q     Max
## -2.4913 -0.4912  0.0247  0.3403  4.1052
##
## Random effects:
##  Groups   Name        Variance  Std.Dev.
##  subj     (Intercept) 0.0016090 0.04011
##  Residual             0.0004889 0.02211
## Number of obs: 114, groups:  subj, 19
##
## Fixed effects:
##              Estimate Std. Error        df t value Pr(>|t|)
## (Intercept)  0.059827   0.010092 23.486034   5.928 4.43e-06 ***
## Vibration2   0.008277   0.005073 92.000000   1.632    0.106
## Vibration3   0.005345   0.005073 92.000000   1.054    0.295
## Vision2      0.034987   0.004142 92.000000   8.447 4.19e-13 ***
## ---
## Signif. codes:  0 '***' 0.001 '**' 0.01 '*' 0.05 '.' 0.1 ' ' 1
##
## Correlation of Fixed Effects:
##            (Intr) Vbrtn2 Vbrtn3
## Vibration2 -0.251
## Vibration3 -0.251  0.500
## Vision2    -0.205  0.000  0.000
```

```r
g6 = ggplot(copv, aes(Vision, AMMVx, colour = Vision, fill = Vision)) +
  stat_summary(fun.data = 'mean_cl_boot', position = position_dodge(),geom = 'bar') +
  stat_summary(fun.data = 'mean_cl_boot', position = position_dodge(0.9), geom = 'errorbar', colour = 'l
print(g6)
```

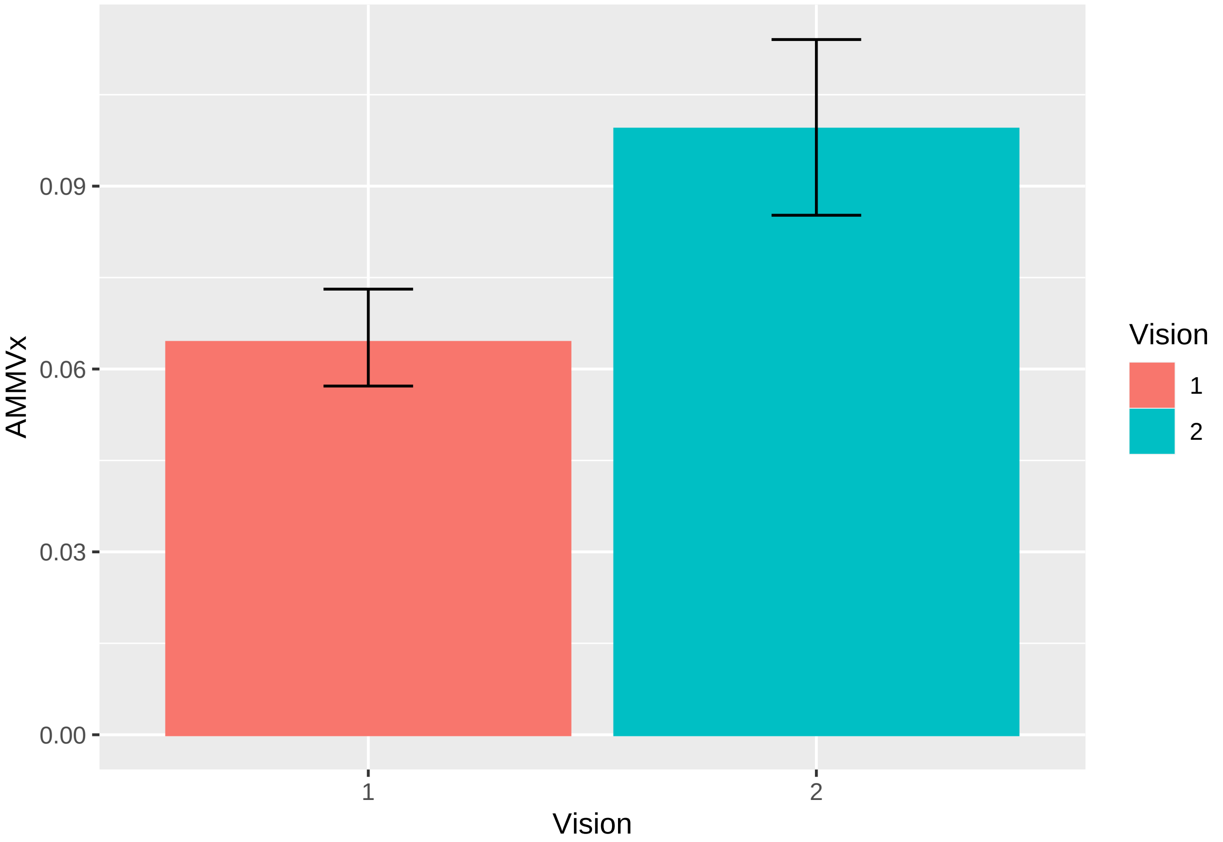


```
# mdoels for RMSx - already in other paper
m0 = lmerTest::lmer(RMSx_ ~ 1 + (1|subj), data = copv)
m1 = lmerTest::lmer(RMSx_ ~ 1 + Vibration + (1|subj), data = copv)
m2 = lmerTest::lmer(RMSx_ ~ 1 + Vibration + Vision + (1|subj), data = copv)
m3 = lmerTest::lmer(RMSx_ ~ 1 + TCThresholdGroup + Vibration + Vision + (1|subj), data = copv)
# m4 = lmerTest::lmer(RMSx_ ~ 1 + Cause + Vibration + Vision + (1|subj), data = copv)
# m5 = lmerTest::lmer(RMSx_ ~ 1 + Mass + Vibration + Vision + (1|subj), data = copv)
# m5 = lmerTest::lmer(RMSx_ ~ 1 + TCThresholdGroup*Vibration + TCThresholdGroup*Vision + (1|subj), data

anova(m0, m1, m2)
```

```
## refitting model(s) with ML (instead of REML)

## Data: copv
## Models:
## m0: RMSx_ ~ 1 + (1 | subj)
## m1: RMSx_ ~ 1 + Vibration + (1 | subj)
## m2: RMSx_ ~ 1 + Vibration + Vision + (1 | subj)
##    npar     AIC     BIC logLik deviance   Chisq Df Pr(>Chisq)
## m0    3 -1073.9 -1065.7 539.95  -1079.9
## m1    5 -1073.5 -1059.9 541.77  -1083.5  3.6432  2     0.1618
## m2    6 -1121.9 -1105.5 566.96  -1133.9 50.3723  1  1.272e-12 ***
## ---
## Signif. codes:  0 '***' 0.001 '**' 0.01 '*' 0.05 '.' 0.1 ' ' 1
```

```
print(summary(m2))
```

```
## Linear mixed model fit by REML. t-tests use Satterthwaite's method [
## lmerModLmerTest]
```

```
## Formula: RMSx_ ~ 1 + Vibration + Vision + (1 | subj)
##    Data: copv
##
## REML criterion at convergence: -1076.9
##
## Scaled residuals:
##      Min       1Q   Median       3Q      Max
## -2.15823 -0.64266 -0.06405  0.64166  2.86744
##
## Random effects:
##  Groups   Name        Variance  Std.Dev.
##  subj     (Intercept) 5.362e-06 0.002316
##  Residual             1.777e-06 0.001333
## Number of obs: 114, groups:  subj, 19
##
## Fixed effects:
##              Estimate Std. Error        df t value Pr(>|t|)
## (Intercept) 5.645e-03  5.870e-04 2.398e+01   9.617 1.06e-09 ***
## Vibration2  6.137e-04  3.059e-04 9.200e+01   2.007   0.0477 *
## Vibration3  6.894e-04  3.059e-04 9.200e+01   2.254   0.0266 *
## Vision2     2.003e-03  2.497e-04 9.200e+01   8.021 3.25e-12 ***
## ---
## Signif. codes:  0 '***' 0.001 '**' 0.01 '*' 0.05 '.' 0.1 ' ' 1
##
## Correlation of Fixed Effects:
##            (Intr) Vbrtn2 Vbrtn3
## Vibration2 -0.261
## Vibration3 -0.261  0.500
## Vision2    -0.213  0.000  0.000
```

```
g7 = ggplot(copv, aes(Vision, RMSx_, colour = Vibration, fill = Vibration)) +
  stat_summary(fun.data = 'mean_cl_boot', position = position_dodge(),geom = 'bar') +
  stat_summary(fun.data = 'mean_cl_boot', position = position_dodge(0.9), geom = 'errorbar', colour = '
print(g7)
```

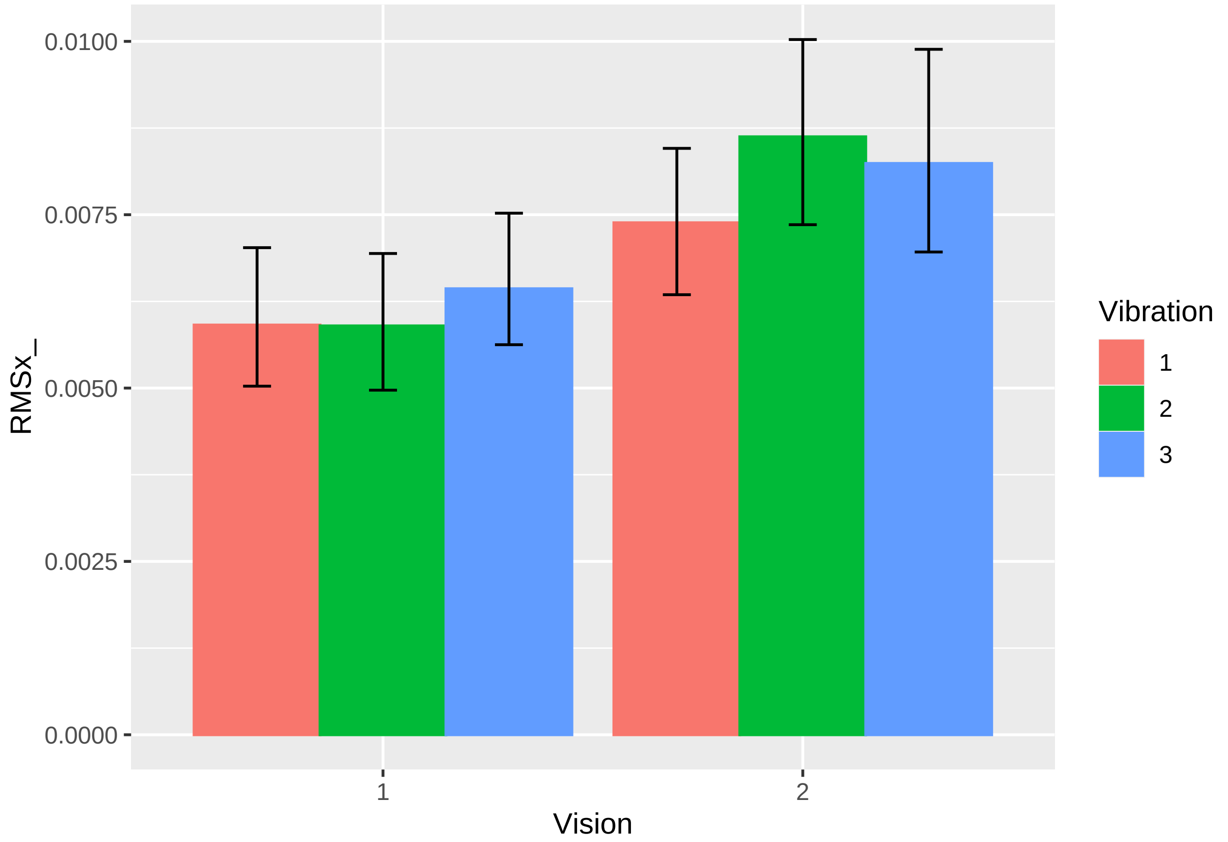


```
# RMSy_ -----------------------------------------------------------------

# model for AMMVx
m0 = lmerTest::lmer(AMMVx ~ 1 + (1|subj), data = copv)
m1 = lmerTest::lmer(AMMVx ~ 1 + Vibration + (1|subj), data = copv)
m2 = lmerTest::lmer(AMMVx ~ 1 + Vibration + Vision + (1|subj), data = copv)
# m3 = lmerTest::lmer(AMMVx ~ 1 + Vibration * Vision + (1|subj), data = copv)
# m3 = lmerTest::lmer(AMMVx ~ 1 + Cause + Vision + Vibration + (1|subj), data = copv)
# m3 = lmerTest::lmer(AMMVx ~ 1 + TCThresholdGroup + Vision + Vibration + (1|subj), data = copv)
# m3 = lmerTest::lmer(AMMVx ~ 1 + Vibration +Vision + Mass + (1|subj), data = copv)
# m5 = lmerTest::lmer(AMMVx ~ 1 + Vibration * Vision + Mass + Mass:Vibration + (1|subj), data = copv)
# m6 = lmerTest::lmer(AMMVx ~ 1 + Mass * Vision * Vibration + (1|subj), data = copv)

anova(m0, m1, m2)
```

```
## refitting model(s) with ML (instead of REML)

## Data: copv
## Models:
## m0: AMMVx ~ 1 + (1 | subj)
## m1: AMMVx ~ 1 + Vibration + (1 | subj)
## m2: AMMVx ~ 1 + Vibration + Vision + (1 | subj)
##    npar     AIC     BIC logLik deviance   Chisq Df Pr(>Chisq)
## m0    3 -429.88 -421.67 217.94  -435.88
## m1    5 -427.46 -413.78 218.73  -437.46  1.5791  2      0.454
## m2    6 -480.00 -463.59 246.00  -492.00 54.5449  1  1.519e-13 ***
## ---
## Signif. codes:  0 '***' 0.001 '**' 0.01 '*' 0.05 '.' 0.1 ' ' 1
```

```r
print(summary(m2))
```

```
## Linear mixed model fit by REML. t-tests use Satterthwaite's method [
## lmerModLmerTest]
## Formula: AMMVx ~ 1 + Vibration + Vision + (1 | subj)
##    Data: copv
##
## REML criterion at convergence: -457.6
##
## Scaled residuals:
##     Min      1Q  Median      3Q     Max
## -2.4913 -0.4912  0.0247  0.3403  4.1052
##
## Random effects:
##  Groups   Name        Variance  Std.Dev.
##  subj     (Intercept) 0.0016090 0.04011
##  Residual             0.0004889 0.02211
## Number of obs: 114, groups:  subj, 19
##
## Fixed effects:
##              Estimate Std. Error        df t value Pr(>|t|)
## (Intercept)  0.059827   0.010092 23.486034   5.928 4.43e-06 ***
## Vibration2   0.008277   0.005073 92.000000   1.632    0.106
## Vibration3   0.005345   0.005073 92.000000   1.054    0.295
## Vision2      0.034987   0.004142 92.000000   8.447 4.19e-13 ***
## ---
## Signif. codes:  0 '***' 0.001 '**' 0.01 '*' 0.05 '.' 0.1 ' ' 1
##
## Correlation of Fixed Effects:
##            (Intr) Vbrtn2 Vbrtn3
## Vibration2 -0.251
## Vibration3 -0.251  0.500
## Vision2    -0.205  0.000  0.000
```

```r
g8 = ggplot(copv, aes(Vision, AMMVx, colour = Vision, fill = Vision)) +
  stat_summary(fun.data = 'mean_cl_boot', position = position_dodge(),geom = 'bar') +
  stat_summary(fun.data = 'mean_cl_boot', position = position_dodge(0.9), geom = 'errorbar', colour = 'I
print(g8)
```

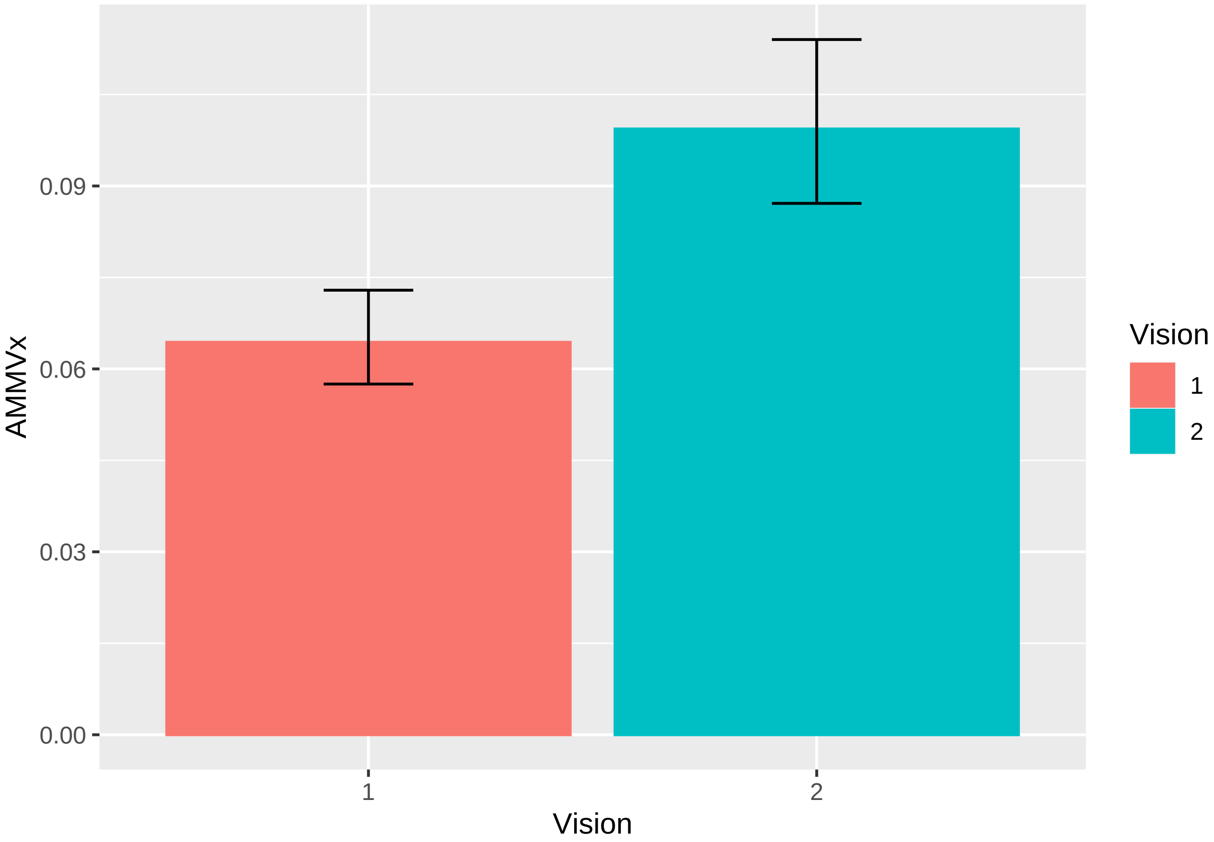


```
# mdoels for RMSx - already in other paper
m0 = lmerTest::lmer(RMSy_ ~ 1 + (1|subj), data = copv)
m1 = lmerTest::lmer(RMSy_ ~ 1 + Vibration + (1|subj), data = copv)
m2 = lmerTest::lmer(RMSy_ ~ 1 + Vibration + Vision + (1|subj), data = copv)
# m3 = lmerTest::lmer(RMSy_ ~ 1 + TCThresholdGroup + Vibration + Vision + (1|subj), data = copv)
# m4 = lmerTest::lmer(RMSy_ ~ 1 + Cause + Vibration + Vision + (1|subj), data = copv)
# m5 = lmerTest::lmer(RMSy_ ~ 1 + Mass + Vibration + Vision + (1|subj), data = copv)
# m5 = lmerTest::lmer(RMSy_ ~ 1 + TCThresholdGroup*Vibration + TCThresholdGroup*Vision + (1|subj), data

anova(m0, m1, m2)
```

```
## refitting model(s) with ML (instead of REML)
```

```
## Data: copv
## Models:
## m0: RMSy_ ~ 1 + (1 | subj)
## m1: RMSy_ ~ 1 + Vibration + (1 | subj)
## m2: RMSy_ ~ 1 + Vibration + Vision + (1 | subj)
##    npar     AIC     BIC logLik deviance   Chisq Df Pr(>Chisq)
## m0    3 -1065.8 -1057.6 535.91  -1071.8
## m1    5 -1063.0 -1049.3 536.49  -1073.0  1.1511  2     0.5624
## m2    6 -1113.1 -1096.7 562.54  -1125.1 52.1087  1  5.251e-13 ***
## ---
## Signif. codes:  0 '***' 0.001 '**' 0.01 '*' 0.05 '.' 0.1 ' ' 1
```

```
print(summary(m2))
```

```
## Linear mixed model fit by REML. t-tests use Satterthwaite's method [
## lmerModLmerTest]
```

```
## Formula: RMSy_ ~ 1 + Vibration + Vision + (1 | subj)
##    Data: copv
##
## REML criterion at convergence: -1068.7
##
## Scaled residuals:
##     Min      1Q  Median      3Q     Max
## -2.8756 -0.5726 -0.0724  0.5896  3.2708
##
## Random effects:
##  Groups   Name        Variance  Std.Dev.
##  subj     (Intercept) 1.201e-05 0.003466
##  Residual             1.670e-06 0.001292
## Number of obs: 114, groups:  subj, 19
##
## Fixed effects:
##              Estimate Std. Error        df t value Pr(>|t|)
## (Intercept) 5.246e-03  8.311e-04 2.051e+01   6.312 3.27e-06 ***
## Vibration2  3.791e-04  2.965e-04 9.200e+01   1.279    0.204
## Vibration3  4.741e-05  2.965e-04 9.200e+01   0.160    0.873
## Vision2     1.985e-03  2.421e-04 9.200e+01   8.199 1.39e-12 ***
## ---
## Signif. codes:  0 '***' 0.001 '**' 0.01 '*' 0.05 '.' 0.1 ' ' 1
##
## Correlation of Fixed Effects:
##            (Intr) Vbrtn2 Vbrtn3
## Vibration2 -0.178
## Vibration3 -0.178  0.500
## Vision2    -0.146  0.000  0.000
```

```
g9 = ggplot(copv, aes(Vision, RMSy_, colour = Vibration, fill = Vibration)) +
  stat_summary(fun.data = 'mean_cl_boot', position = position_dodge(),geom = 'bar') +
  stat_summary(fun.data = 'mean_cl_boot', position = position_dodge(0.9), geom = 'errorbar', colour = '
print(g9)
```

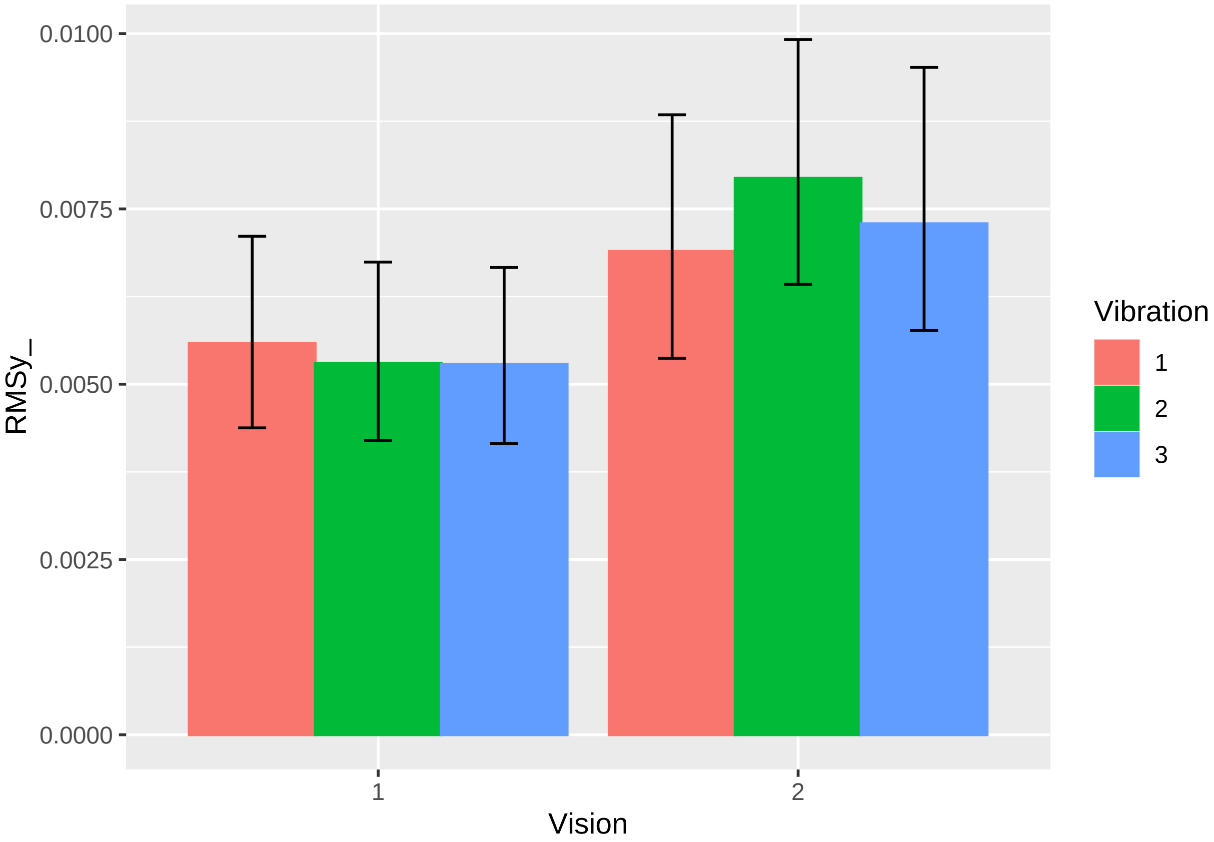


```r
# volecityx ------------------------------------------------------------------

# model for AMMVx
m0 = lmerTest::lmer(AMMVx ~ 1 + (1|subj), data = copv)
m1 = lmerTest::lmer(AMMVx ~ 1 + Vibration + (1|subj), data = copv)
m2 = lmerTest::lmer(AMMVx ~ 1 + Vibration + Vision + (1|subj), data = copv)
# m3 = lmerTest::lmer(AMMVx ~ 1 + Vibration * Vision + (1|subj), data = copv)
# m3 = lmerTest::lmer(AMMVx ~ 1 + Cause + Vision + Vibration + (1|subj), data = copv)
# m3 = lmerTest::lmer(AMMVx ~ 1 + TCThresholdGroup + Vision + Vibration + (1|subj), data = copv)
# m3 = lmerTest::lmer(AMMVx ~ 1 + Vibration +Vision + Mass + (1|subj), data = copv)
# m5 = lmerTest::lmer(AMMVx ~ 1 + Vibration * Vision + Mass + Mass:Vibration + (1|subj), data = copv)
#m6 = lmerTest::lmer(AMMVx ~ 1 + Mass * Vision * Vibration + (1|subj), data = copv)

anova(m0, m1, m2)
```

```
## refitting model(s) with ML (instead of REML)

## Data: copv
## Models:
## m0: AMMVx ~ 1 + (1 | subj)
## m1: AMMVx ~ 1 + Vibration + (1 | subj)
## m2: AMMVx ~ 1 + Vibration + Vision + (1 | subj)
##    npar     AIC     BIC logLik deviance   Chisq Df Pr(>Chisq)
## m0    3 -429.88 -421.67 217.94  -435.88
## m1    5 -427.46 -413.78 218.73  -437.46  1.5791  2      0.454
## m2    6 -480.00 -463.59 246.00  -492.00 54.5449  1  1.519e-13 ***
## ---
## Signif. codes:  0 '***' 0.001 '**' 0.01 '*' 0.05 '.' 0.1 ' ' 1
```

```r
print(summary(m2))
```

```
## Linear mixed model fit by REML. t-tests use Satterthwaite's method [
## lmerModLmerTest]
## Formula: AMMVx ~ 1 + Vibration + Vision + (1 | subj)
##    Data: copv
##
## REML criterion at convergence: -457.6
##
## Scaled residuals:
##     Min      1Q  Median      3Q     Max
## -2.4913 -0.4912  0.0247  0.3403  4.1052
##
## Random effects:
##  Groups   Name        Variance  Std.Dev.
##  subj     (Intercept) 0.0016090 0.04011
##  Residual             0.0004889 0.02211
## Number of obs: 114, groups:  subj, 19
##
## Fixed effects:
##              Estimate Std. Error        df t value Pr(>|t|)
## (Intercept)  0.059827   0.010092 23.486034   5.928 4.43e-06 ***
## Vibration2   0.008277   0.005073 92.000000   1.632    0.106
## Vibration3   0.005345   0.005073 92.000000   1.054    0.295
## Vision2      0.034987   0.004142 92.000000   8.447 4.19e-13 ***
## ---
## Signif. codes:  0 '***' 0.001 '**' 0.01 '*' 0.05 '.' 0.1 ' ' 1
##
## Correlation of Fixed Effects:
##            (Intr) Vbrtn2 Vbrtn3
## Vibration2 -0.251
## Vibration3 -0.251  0.500
## Vision2    -0.205  0.000  0.000
```

```r
g10 = ggplot(copv, aes(Vision, AMMVx, colour = Vision, fill = Vision)) +
  stat_summary(fun.data = 'mean_cl_boot', position = position_dodge(),geom = 'bar') +
  stat_summary(fun.data = 'mean_cl_boot', position = position_dodge(0.9), geom = 'errorbar', colour = 'l
print(g10)
```

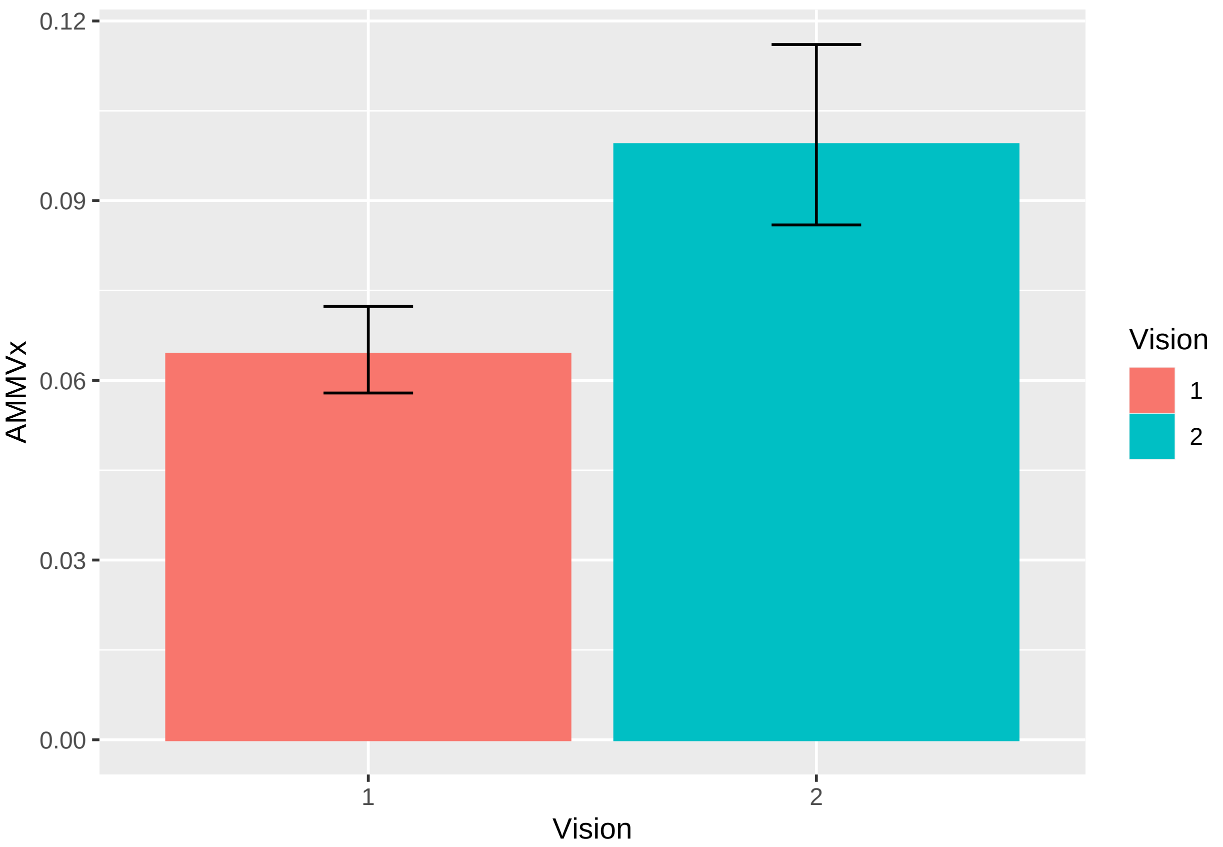


```
# mdoels for RMSx - already in other paper
m0 = lmerTest::lmer(Velocityx ~ 1 + (1|subj), data = copv)
m1 = lmerTest::lmer(Velocityx ~ 1 + Vibration + (1|subj), data = copv)
m2 = lmerTest::lmer(Velocityx ~ 1 + Vibration + Vision + (1|subj), data = copv)
# m3 = lmerTest::lmer(Velocityx ~ 1 + Vibration * Vision + (1|subj), data = copv)
# m3 = lmerTest::lmer(Velocityx ~ 1 + TCThresholdGroup + Vibration + Vision + (1|subj), data = copv)
m4 = lmerTest::lmer(Velocityx ~ 1 + Cause + Vibration + Vision + (1|subj), data = copv)
# m5 = lmerTest::lmer(Velocityx ~ 1 + Mass + Vibration + Vision + (1|subj), data = copv)
# m6 = lmerTest::lmer(Velocityx ~ 1 + TCThresholdGroup*Vibration + TCThresholdGroup*Vision + (1|subj),
m7 = lmerTest::lmer(Velocityx ~ 1 + Cause*Vibration + Cause*Vision + (1|subj), data = copv)
anova(m0, m1, m2, m3, m7)
```

```
## refitting model(s) with ML (instead of REML)

## Data: copv
## Models:
## m0: Velocityx ~ 1 + (1 | subj)
## m1: Velocityx ~ 1 + Vibration + (1 | subj)
## m2: Velocityx ~ 1 + Vibration + Vision + (1 | subj)
## m3: RMSx_ ~ 1 + TCThresholdGroup + Vibration + Vision + (1 | subj)
## m7: Velocityx ~ 1 + Cause * Vibration + Cause * Vision + (1 | subj)
##    npar      AIC      BIC logLik deviance   Chisq Df Pr(>Chisq)
## m0    3  -698.73  -690.52 352.36  -704.73
## m1    5  -695.96  -682.28 352.98  -705.96   1.237  2     0.5387
## m2    6  -744.93  -728.52 378.47  -756.93  50.970  1  9.381e-13 ***
## m3    7 -1124.27 -1105.11 569.13 -1138.27 381.334  1  < 2.2e-16 ***
## m7   14  -747.41  -709.10 387.70  -775.41   0.000  7     1.0000
## ---
```

```
## Signif. codes:  0 '***' 0.001 '**' 0.01 '*' 0.05 '.' 0.1 ' ' 1
```

```r
print(summary(m7))
```

```
## Linear mixed model fit by REML. t-tests use Satterthwaite's method [
## lmerModLmerTest]
## Formula: Velocityx ~ 1 + Cause * Vibration + Cause * Vision + (1 | subj)
##    Data: copv
##
## REML criterion at convergence: -655.9
##
## Scaled residuals:
##     Min      1Q  Median      3Q     Max
## -1.9860 -0.3464 -0.0919  0.2834  4.7935
##
## Random effects:
##  Groups   Name        Variance  Std.Dev.
##  subj     (Intercept) 1.319e-04 0.011483
##  Residual             4.466e-05 0.006683
## Number of obs: 114, groups:  subj, 19
##
## Fixed effects:
##                      Estimate Std. Error         df t value Pr(>|t|)
## (Intercept)         0.0130605  0.0048052 21.4389857   2.718 0.012738 *
## Cause2              0.0055047  0.0074442 21.4389857   0.739 0.467642
## Cause3              0.0059369  0.0067956 21.4389857   0.874 0.391997
## Vibration2         -0.0006858  0.0025260 86.0000001  -0.272 0.786655
## Vibration3         -0.0012578  0.0025260 86.0000001  -0.498 0.619804
## Vision2             0.0077448  0.0020625 86.0000001   3.755 0.000314 ***
## Cause2:Vibration2   0.0011889  0.0039133 86.0000001   0.304 0.762001
## Cause3:Vibration2   0.0071440  0.0035723 86.0000001   2.000 0.048673 *
## Cause2:Vibration3   0.0017580  0.0039133 86.0000001   0.449 0.654392
## Cause3:Vibration3   0.0060125  0.0035723 86.0000001   1.683 0.095983 .
## Cause2:Vision2      0.0001505  0.0031952 86.0000001   0.047 0.962536
## Cause3:Vision2      0.0073595  0.0029168 86.0000001   2.523 0.013470 *
## ---
## Signif. codes:  0 '***' 0.001 '**' 0.01 '*' 0.05 '.' 0.1 ' ' 1
##
## Correlation of Fixed Effects:
##             (Intr) Cause2 Cause3 Vbrtn2 Vbrtn3 Visin2 Cs2:Vb2 Cs3:Vb2 Cs2:V3
## Cause2      -0.645
## Cause3      -0.707  0.456
## Vibration2  -0.263  0.170  0.186
## Vibration3  -0.263  0.170  0.186  0.500
## Vision2     -0.215  0.139  0.152  0.000  0.000
## Cas2:Vbrtn2  0.170 -0.263 -0.120 -0.645 -0.323  0.000
## Cas3:Vbrtn2  0.186 -0.120 -0.263 -0.707 -0.354  0.000  0.456
## Cas2:Vbrtn3  0.170 -0.263 -0.120 -0.323 -0.645  0.000  0.500   0.228
## Cas3:Vbrtn3  0.186 -0.120 -0.263 -0.354 -0.707  0.000  0.228   0.500   0.456
## Cause2:Vsn2  0.139 -0.215 -0.098  0.000  0.000 -0.645  0.000   0.000   0.000
## Cause3:Vsn2  0.152 -0.098 -0.215  0.000  0.000 -0.707  0.000   0.000   0.000
##             Cs3:V3 Cs2:Vs2
## Cause2
## Cause3
## Vibration2
```

```
## Vibration3
## Vision2
## Cas2:Vbrtn2
## Cas3:Vbrtn2
## Cas2:Vbrtn3
## Cas3:Vbrtn3
## Cause2:Vsn2  0.000
## Cause3:Vsn2  0.000  0.456
```

```
g11 = ggplot(copv, aes(Vision, Velocityx, colour = Vibration, fill = Vibration)) +
  stat_summary(fun.data = 'mean_cl_boot', position = position_dodge(),geom = 'bar') +
  stat_summary(fun.data = 'mean_cl_boot', position = position_dodge(0.9), geom = 'errorbar', colour = 'l
  facet_wrap(~Cause)
print(g11)
```

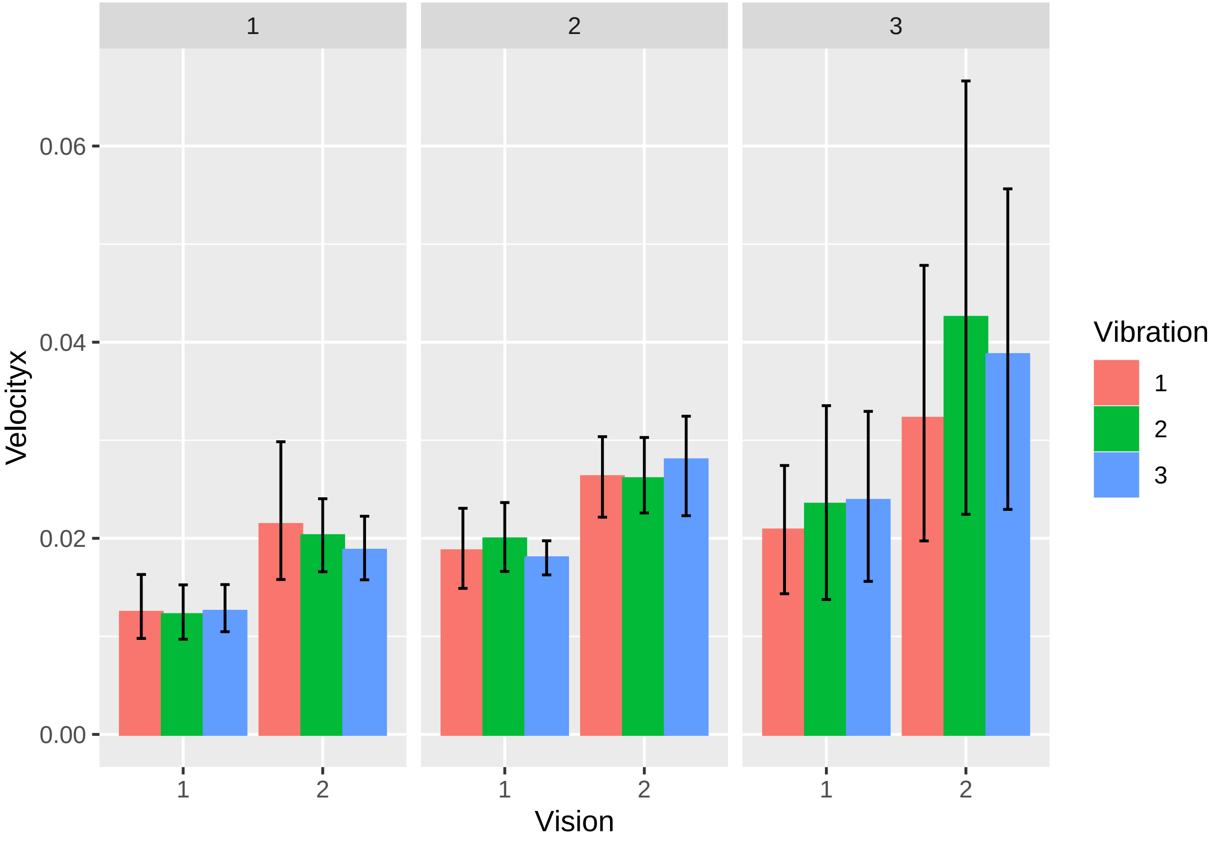


```
# x_Dxl_ ------------------------------------------------------------------

# m0 = lmerTest::lmer(x_Dxl_ ~ 1 + (1|subj), data = copv)
# m1 = lmerTest::lmer(x_Dxl_ ~ 1 + Vibration + (1|subj), data = copv)
# m2 = lmerTest::lmer(x_Dxl_ ~ 1 + Vibration + Vision + (1|subj), data = copv)
# # m3 = lmerTest::lmer(x_Dxl_ ~ 1 + Vibration * Vision + (1|subj), data = copv)
# # m4 = lmerTest::lmer(x_Dxl_ ~ 1 + TCThresholdGroup + Vibration + Vision + (1|subj), data = copv)
# m5 = lmerTest::lmer(x_Dxl_ ~ 1 + Cause + Vibration + Vision + (1|subj), data = copv)
# # m6 = lmerTest::lmer(x_Dxl_ ~ 1 + Mass + Vibration + Vision + (1|subj), data = copv)
# m8 = lmerTest::lmer(x_Dxl_ ~ 1 + Cause*Vibration + Cause*Vision + (1|subj), data = copv)
# anova(m0, m1, m2, m5, m8)
# print(summary(m8))
#
```

```r
# g12 = ggplot(copv, aes(Vision, x, colour = Vibration, fill = Vibration)) +
#   stat_summary(fun.data = 'mean_cl_boot', position = position_dodge(),geom = 'bar') +
#   stat_summary(fun.data = 'mean_cl_boot', position = position_dodge(0.9), geom = 'errorbar', colour =
#   facet_wrap(~Cause)
# print(g12)


# Drs ---------------------------------------------------------------------

m0 = lmerTest::lmer(Drs ~ 1 + (1|subj), data = copv)
m1 = lmerTest::lmer(Drs ~ 1 + Vibration + (1|subj), data = copv)
m2 = lmerTest::lmer(Drs ~ 1 + Vibration + Vision + (1|subj), data = copv)
# m3 = lmerTest::lmer(Drs ~ 1 + Vibration * Vision + (1|subj), data = copv)
# m3 = lmerTest::lmer(Drs ~ 1 + TCThresholdGroup + Vibration + Vision + (1|subj), data = copv)
# m4 = lmerTest::lmer(Drs ~ 1 + Cause + Vibration + Vision + (1|subj), data = copv)
# m5 = lmerTest::lmer(Drs ~ 1 + Mass + Vibration + Vision + (1|subj), data = copv)
# m6 = lmerTest::lmer(Drs ~ 1 + TCThresholdGroup*Vibration + TCThresholdGroup*Vision + (1|subj), data =
# m7 = lmerTest::lmer(Drs ~ 1 + Cause*Vibration + Cause*Vision + (1|subj), data = copv)
anova(m0, m1, m2)
```

```
## refitting model(s) with ML (instead of REML)

## Data: copv
## Models:
## m0: Drs ~ 1 + (1 | subj)
## m1: Drs ~ 1 + Vibration + (1 | subj)
## m2: Drs ~ 1 + Vibration + Vision + (1 | subj)
##    npar     AIC     BIC logLik deviance   Chisq Df Pr(>Chisq)
## m0    3 -1673.5 -1665.2 839.72  -1679.5
## m1    5 -1672.7 -1659.0 841.36  -1682.7  3.2667  2     0.1953
## m2    6 -1694.9 -1678.5 853.46  -1706.9 24.2047  1  8.662e-07 ***
## ---
## Signif. codes:  0 '***' 0.001 '**' 0.01 '*' 0.05 '.' 0.1 ' ' 1
```

```r
print(summary(m2))
```

```
## Linear mixed model fit by REML. t-tests use Satterthwaite's method [
## lmerModLmerTest]
## Formula: Drs ~ 1 + Vibration + Vision + (1 | subj)
##    Data: copv
##
## REML criterion at convergence: -1629.8
##
## Scaled residuals:
##     Min      1Q  Median      3Q     Max
## -3.2327 -0.3492 -0.0160  0.2602  6.1634
##
## Random effects:
##  Groups   Name        Variance  Std.Dev.
##  subj     (Intercept) 3.038e-08 0.0001743
##  Residual             1.199e-08 0.0001095
## Number of obs: 114, groups:  subj, 19
##
## Fixed effects:
##                Estimate Std. Error        df t value Pr(>|t|)
```

```
## (Intercept) 6.173e-05  4.494e-05 2.511e+01   1.374   0.1817
## Vibration2  4.645e-05  2.512e-05 9.200e+01   1.849   0.0677 .
## Vibration3  4.186e-05  2.512e-05 9.200e+01   1.666   0.0991 .
## Vision2     1.060e-04  2.051e-05 9.200e+01   5.167 1.37e-06 ***
## ---
## Signif. codes:  0 '***' 0.001 '**' 0.01 '*' 0.05 '.' 0.1 ' ' 1
##
## Correlation of Fixed Effects:
##             (Intr) Vbrtn2 Vbrtn3
## Vibration2 -0.279
## Vibration3 -0.279  0.500
## Vision2    -0.228  0.000  0.000
```

```
g13 = ggplot(copv, aes(Vision, Drs, colour = Vision, fill = Vision)) +
  stat_summary(fun.data = 'mean_cl_boot', position = position_dodge(),geom = 'bar') +
  stat_summary(fun.data = 'mean_cl_boot', position = position_dodge(0.9), geom = 'errorbar', colour = 'l
print(g13)
```

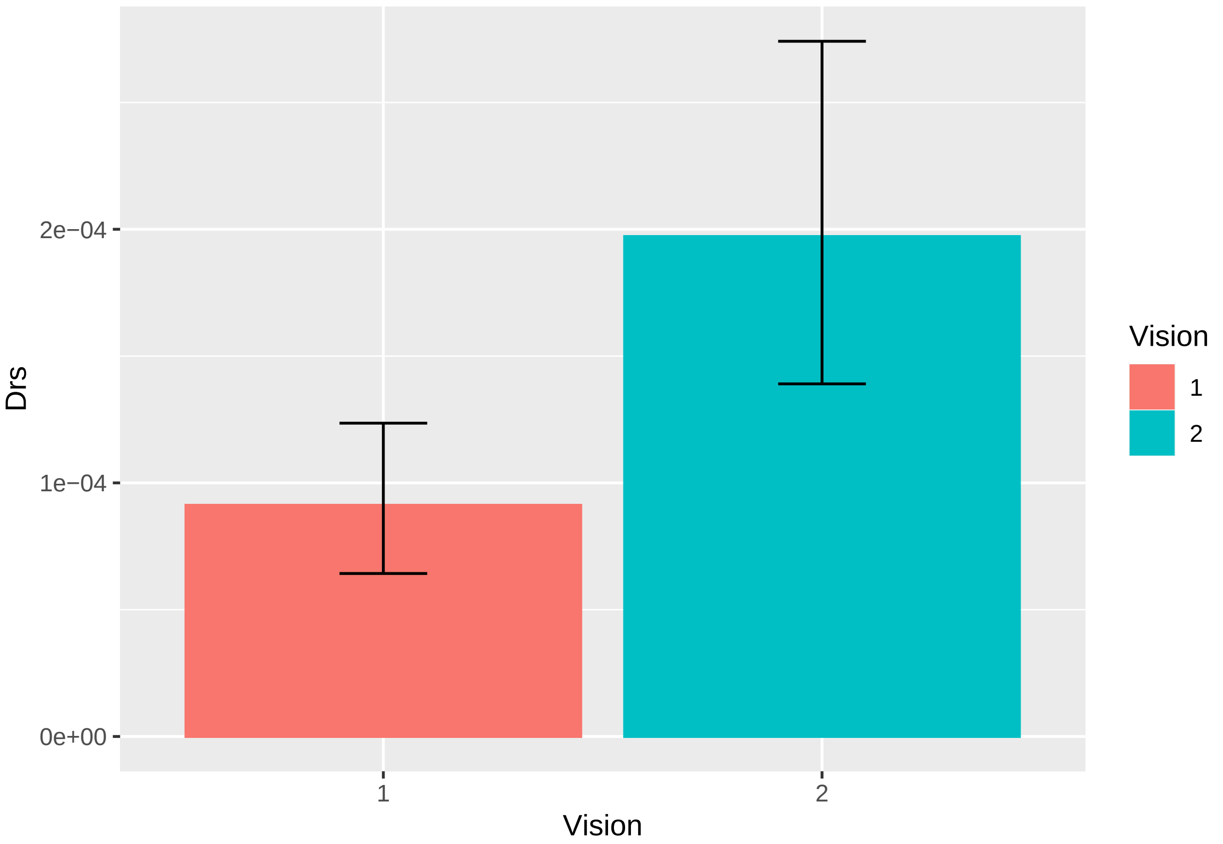


```
# x_Drl_ ------------------------------------------------------------------
m0 = lmerTest::lmer(x_Drl_ ~ 1 + (1|subj), data = copv)
m1 = lmerTest::lmer(x_Drl_ ~ 1 + Vibration + (1|subj), data = copv)
m2 = lmerTest::lmer(x_Drl_ ~ 1 + Vibration + Vision + (1|subj), data = copv)
# m3 = lmerTest::lmer(x_Drl_ ~ 1 + Vibration * Vision + (1|subj), data = copv)
m3 = lmerTest::lmer(x_Drl_ ~ 1 + TCThresholdGroup + Vibration + Vision + (1|subj), data = copv)
# m4 = lmerTest::lmer(x_Drl_ ~ 1 + Cause + Vibration + Vision + (1|subj), data = copv)
# m5 = lmerTest::lmer(x_Drl_ ~ 1 + Mass + Vibration + Vision + (1|subj), data = copv)
# m6 = lmerTest::lmer(x_Drl_ ~ 1 + TCThresholdGroup*Vibration + TCThresholdGroup*Vision + (1|subj), dat
# m7 = lmerTest::lmer(x_Drl_ ~ 1 + Cause*Vibration + Cause*Vision + (1|subj), data = copv)
```

```r
anova(m0, m1, m2, m3, m7)
```

```
## refitting model(s) with ML (instead of REML)

## Data: copv
## Models:
## m0: x_Drl_ ~ 1 + (1 | subj)
## m1: x_Drl_ ~ 1 + Vibration + (1 | subj)
## m2: x_Drl_ ~ 1 + Vibration + Vision + (1 | subj)
## m3: x_Drl_ ~ 1 + TCThresholdGroup + Vibration + Vision + (1 | subj)
## m7: Velocityx ~ 1 + Cause * Vibration + Cause * Vision + (1 | subj)
##    npar      AIC     BIC logLik deviance  Chisq Df Pr(>Chisq)
## m0    3 -2755.56 -2747.3 1380.8 -2761.56
## m1    5 -2751.57 -2737.9 1380.8 -2761.57 0.0109  2    0.99458
## m2    6 -2754.13 -2737.7 1383.1 -2766.13 4.5581  1    0.03276 *
## m3    7 -2756.94 -2737.8 1385.5 -2770.94 4.8141  1    0.02823 *
## m7   14  -747.41  -709.1  387.7  -775.41 0.0000  7    1.00000
## ---
## Signif. codes:  0 '***' 0.001 '**' 0.01 '*' 0.05 '.' 0.1 ' ' 1
```

```r
print(summary(m7))
```

```
## Linear mixed model fit by REML. t-tests use Satterthwaite's method [
## lmerModLmerTest]
## Formula: Velocityx ~ 1 + Cause * Vibration + Cause * Vision + (1 | subj)
##    Data: copv
##
## REML criterion at convergence: -655.9
##
## Scaled residuals:
##     Min      1Q  Median      3Q     Max
## -1.9860 -0.3464 -0.0919  0.2834  4.7935
##
## Random effects:
##  Groups   Name        Variance  Std.Dev.
##  subj     (Intercept) 1.319e-04 0.011483
##  Residual             4.466e-05 0.006683
## Number of obs: 114, groups:  subj, 19
##
## Fixed effects:
##                      Estimate Std. Error         df t value Pr(>|t|)
## (Intercept)         0.0130605  0.0048052 21.4389857   2.718 0.012738 *
## Cause2              0.0055047  0.0074442 21.4389857   0.739 0.467642
## Cause3              0.0059369  0.0067956 21.4389857   0.874 0.391997
## Vibration2         -0.0006858  0.0025260 86.0000001  -0.272 0.786655
## Vibration3         -0.0012578  0.0025260 86.0000001  -0.498 0.619804
## Vision2             0.0077448  0.0020625 86.0000001   3.755 0.000314 ***
## Cause2:Vibration2   0.0011889  0.0039133 86.0000001   0.304 0.762001
## Cause3:Vibration2   0.0071440  0.0035723 86.0000001   2.000 0.048673 *
## Cause2:Vibration3   0.0017580  0.0039133 86.0000001   0.449 0.654392
## Cause3:Vibration3   0.0060125  0.0035723 86.0000001   1.683 0.095983 .
## Cause2:Vision2      0.0001505  0.0031952 86.0000001   0.047 0.962536
## Cause3:Vision2      0.0073595  0.0029168 86.0000001   2.523 0.013470 *
## ---
## Signif. codes:  0 '***' 0.001 '**' 0.01 '*' 0.05 '.' 0.1 ' ' 1
```

```
##
## Correlation of Fixed Effects:
##              (Intr) Cause2 Cause3 Vbrtn2 Vbrtn3 Visin2 Cs2:Vb2 Cs3:Vb2 Cs2:V3
## Cause2       -0.645
## Cause3       -0.707  0.456
## Vibration2   -0.263  0.170  0.186
## Vibration3   -0.263  0.170  0.186  0.500
## Vision2      -0.215  0.139  0.152  0.000  0.000
## Cas2:Vbrtn2   0.170 -0.263 -0.120 -0.645 -0.323  0.000
## Cas3:Vbrtn2   0.186 -0.120 -0.263 -0.707 -0.354  0.000  0.456
## Cas2:Vbrtn3   0.170 -0.263 -0.120 -0.323 -0.645  0.000  0.500   0.228
## Cas3:Vbrtn3   0.186 -0.120 -0.263 -0.354 -0.707  0.000  0.228   0.500   0.456
## Cause2:Vsn2   0.139 -0.215 -0.098  0.000  0.000 -0.645  0.000   0.000   0.000
## Cause3:Vsn2   0.152 -0.098 -0.215  0.000  0.000 -0.707  0.000   0.000   0.000
##              Cs3:V3 Cs2:Vs2
## Cause2
## Cause3
## Vibration2
## Vibration3
## Vision2
## Cas2:Vbrtn2
## Cas3:Vbrtn2
## Cas2:Vbrtn3
## Cas3:Vbrtn3
## Cause2:Vsn2   0.000
## Cause3:Vsn2   0.000  0.456
```

```
g14 = ggplot(copv, aes(Vision, x_Drl_, colour = Vibration, fill = Vibration)) +
  stat_summary(fun.data = 'mean_cl_boot', position = position_dodge(),geom = 'bar') +
  stat_summary(fun.data = 'mean_cl_boot', position = position_dodge(0.9), geom = 'errorbar', colour = '
  facet_wrap(~TCThresholdGroup)
print(g14)
```

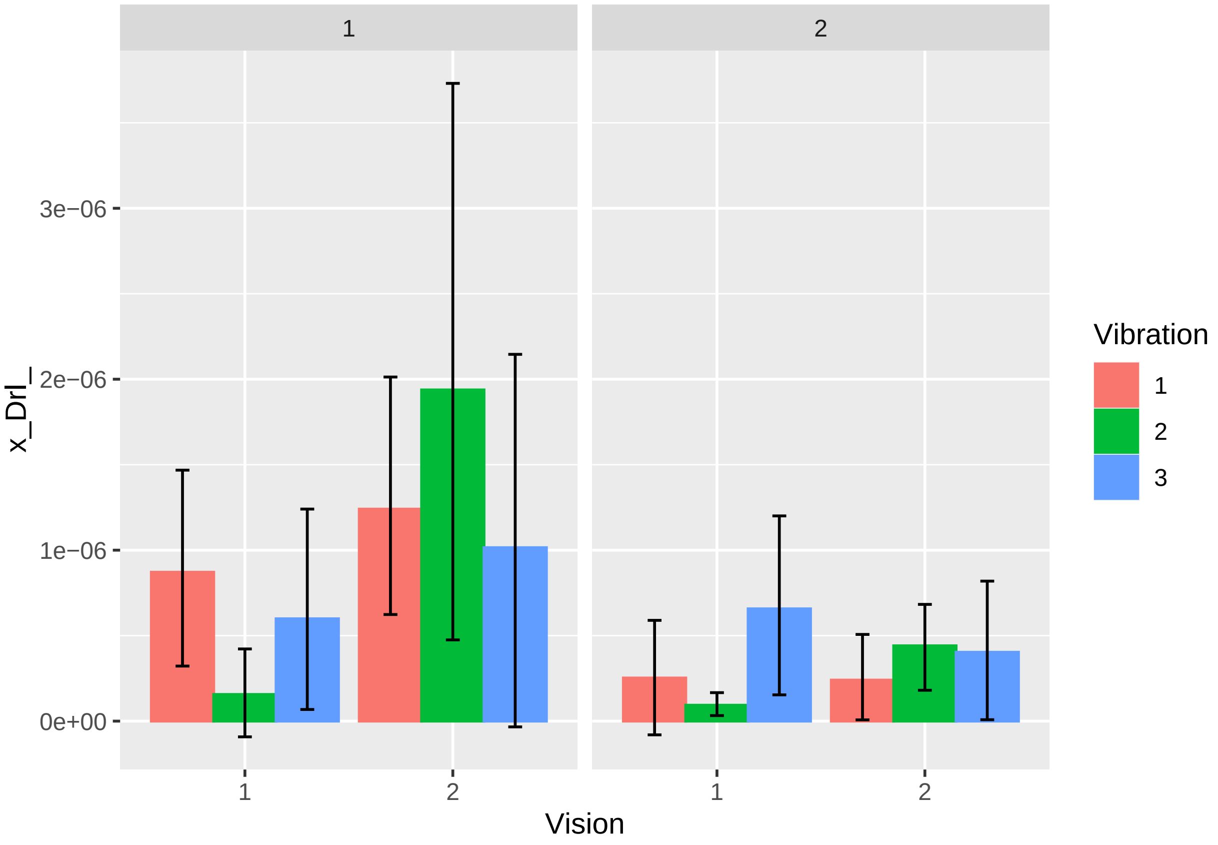


```r
# Hsx ---------------------------------------------------------------------
# mdoels for RMSx - already in other paper
# m0 = lmerTest::lmer(Hxs ~ 1 + (1|subj), data = copv)
# m1 = lmerTest::lmer(Hxs ~ 1 + Vibration + (1|subj), data = copv)
# m2 = lmerTest::lmer(Hxs ~ 1 + Vibration + Vision + (1|subj), data = copv)
# m3 = lmerTest::lmer(Hxs ~ 1 + Vibration * Vision + (1|subj), data = copv)


# x_Hxl -------------------------------------------------------------------
#
# m0 = lmerTest::lmer(x_Hxl_ ~ 1 + (1|subj), data = copv)
# m1 = lmerTest::lmer(x_Hxl_ ~ 1 + Vibration + (1|subj), data = copv)
# m2 = lmerTest::lmer(x_Hxl_ ~ 1 + Vibration + Vision + (1|subj), data = copv)
# m3 = lmerTest::lmer(x_Hxl_ ~ 1 + Vibration * Vision + (1|subj), data = copv)


# Hrs ---------------------------------------------------------------------
m0 = lmerTest::lmer(Hrs ~ 1 + (1|subj), data = copv)
m1 = lmerTest::lmer(Hrs ~ 1 + Vibration + (1|subj), data = copv)
m2 = lmerTest::lmer(Hrs ~ 1 + Vibration + Vision + (1|subj), data = copv)
# m3 = lmerTest::lmer(Hrs ~ 1 + Vibration * Vision + (1|subj), data = copv)
# m3 = lmerTest::lmer(Hrs ~ 1 + TCThresholdGroup + Vibration + Vision + (1|subj), data = copv)
# m4 = lmerTest::lmer(Hrs ~ 1 + Cause + Vibration + Vision + (1|subj), data = copv)
m5 = lmerTest::lmer(Hrs ~ 1 + zMass + Vibration + Vision + (1|subj), data = copv)
m6 = lmerTest::lmer(Hrs ~ 1 + zMass + zMass2 + Vibration + Vision + (1|subj), data = copv)
m7 = lmerTest::lmer(Hrs ~ 1 + Vibration*zMass2 + Vision*zMass2 + (1|subj), data = copv)
anova(m0, m1, m2, m5, m6)
```

```
## refitting model(s) with ML (instead of REML)

## Data: copv
## Models:
## m0: Hrs ~ 1 + (1 | subj)
## m1: Hrs ~ 1 + Vibration + (1 | subj)
## m2: Hrs ~ 1 + Vibration + Vision + (1 | subj)
## m5: Hrs ~ 1 + zMass + Vibration + Vision + (1 | subj)
## m6: Hrs ~ 1 + zMass + zMass2 + Vibration + Vision + (1 | subj)
##    npar     AIC     BIC logLik deviance   Chisq Df Pr(>Chisq)
## m0    3 -378.37 -370.16 192.19  -384.37
## m1    5 -375.19 -361.51 192.59  -385.19  0.8180  2   0.664300
## m2    6 -380.41 -363.99 196.21  -392.41  7.2203  1   0.007208 **
## m5    7 -388.51 -369.35 201.25  -402.51 10.0949  1   0.001487 **
## m6    8 -391.39 -369.50 203.70  -407.39  4.8869  1   0.027062 *
## ---
## Signif. codes:  0 '***' 0.001 '**' 0.01 '*' 0.05 '.' 0.1 ' ' 1
```

```
print(summary(m6))
```

```
## Linear mixed model fit by REML. t-tests use Satterthwaite's method [
## lmerModLmerTest]
## Formula: Hrs ~ 1 + zMass + zMass2 + Vibration + Vision + (1 | subj)
##    Data: copv
##
## REML criterion at convergence: -358.6
##
## Scaled residuals:
##     Min      1Q  Median      3Q     Max
## -3.3377 -0.6067  0.0661  0.5805  2.5200
##
## Random effects:
##  Groups   Name        Variance  Std.Dev.
##  subj     (Intercept) 0.0006068 0.02463
##  Residual             0.0014027 0.03745
## Number of obs: 114, groups:  subj, 19
##
## Fixed effects:
##               Estimate Std. Error         df t value Pr(>|t|)
## (Intercept)  0.7175269  0.0107420 33.3088489  66.796  < 2e-16 ***
## zMass        0.0199024  0.0071540 15.9999998   2.782  0.01333 *
## zMass2      -0.0127886  0.0059034 15.9999998  -2.166  0.04573 *
## Vibration2   0.0066163  0.0085921 92.0000001   0.770  0.44324
## Vibration3  -0.0005258  0.0085921 92.0000001  -0.061  0.95134
## Vision2      0.0189089  0.0070154 92.0000001   2.695  0.00836 **
## ---
## Signif. codes:  0 '***' 0.001 '**' 0.01 '*' 0.05 '.' 0.1 ' ' 1
##
## Correlation of Fixed Effects:
##            (Intr) zMass  zMass2 Vbrtn2 Vbrtn3
## zMass      -0.195
## zMass2     -0.545  0.358
## Vibration2 -0.400  0.000  0.000
## Vibration3 -0.400  0.000  0.000  0.500
## Vision2    -0.327  0.000  0.000  0.000  0.000
```

```r
g15 = ggplot(copv, aes(Vision, Hrs, colour = Vibration, fill = Vibration)) +
  stat_summary(fun.data = 'mean_cl_boot', position = position_dodge(),geom = 'bar') +
  stat_summary(fun.data = 'mean_cl_boot', position = position_dodge(0.9), geom = 'errorbar', colour = 'I
  facet_wrap(~Cause)
print(g15)
```

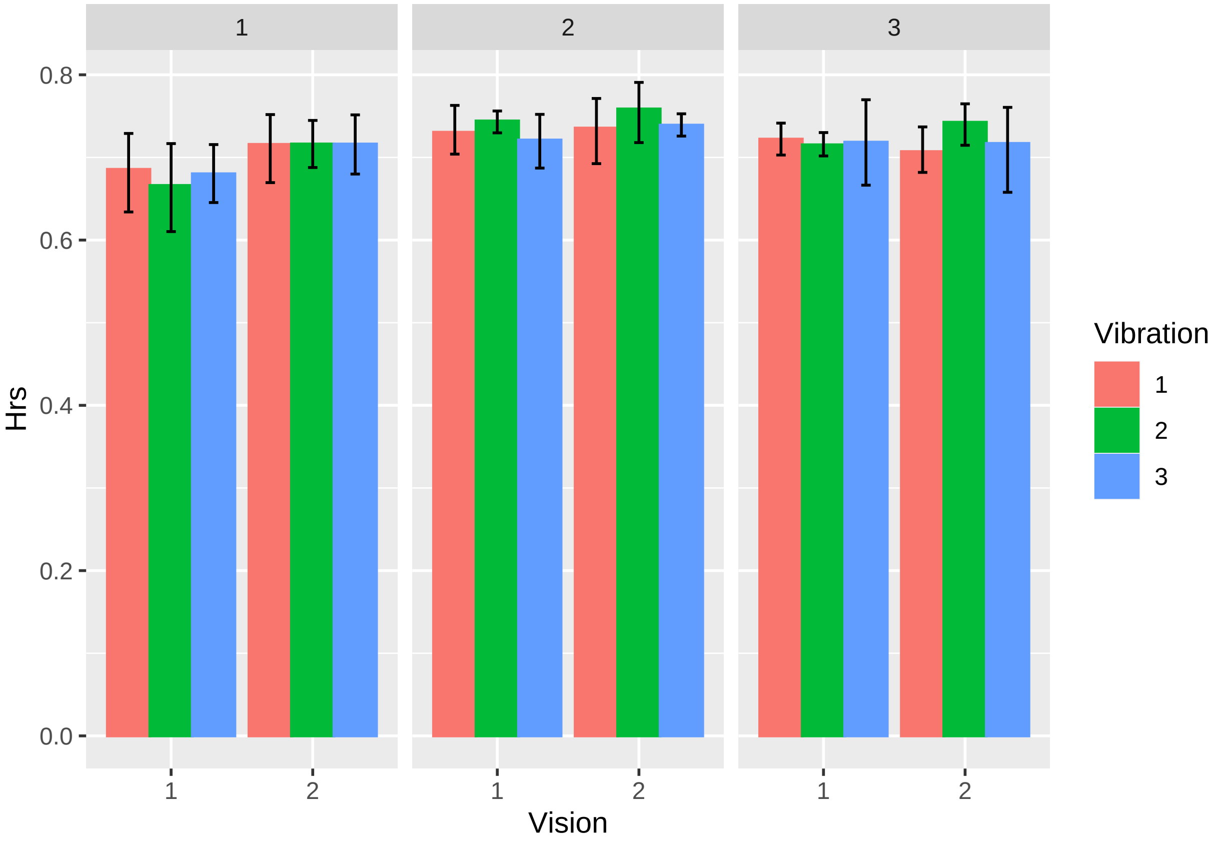


```r
# x_Hrl_ -----------------------------------------------------------------
# m0 = lmerTest::lmer(x_Hrl_ ~ 1 + (1|subj), data = copv)
# m1 = lmerTest::lmer(x_Hrl_ ~ 1 + Vibration + (1|subj), data = copv)
# m2 = lmerTest::lmer(x_Hrl_ ~ 1 + Vibration + Vision + (1|subj), data = copv)
# m3 = lmerTest::lmer(x_Hrl_ ~ 1 + Vibration * Vision + (1|subj), data = copv)


# critX ------------------------------------------------------------------

# m0 = lmerTest::lmer(critX ~ 1 + (1|subj), data = copv)
# m1 = lmerTest::lmer(critX ~ 1 + Vibration + (1|subj), data = copv)
# m2 = lmerTest::lmer(critX ~ 1 + Vibration + Vision + (1|subj), data = copv)
# m3 = lmerTest::lmer(critX ~ 1 + Vibration * Vision + (1|subj), data = copv)


# critR ------------------------------------------------------------------

# m0 = lmerTest::lmer(critR ~ 1 + (1|subj), data = copv)
# m1 = lmerTest::lmer(critR ~ 1 + Vibration + (1|subj), data = copv)
# m2 = lmerTest::lmer(critR ~ 1 + Vibration + Vision + (1|subj), data = copv)
# m3 = lmerTest::lmer(critR ~ 1 + Vibration * Vision + (1|subj), data = copv)
```

```r
# dx2 ----------------------------------------------------------------------

m0 = lmerTest::lmer(dx2 ~ 1 + (1|subj), data = copv)
m1 = lmerTest::lmer(dx2 ~ 1 + Vibration + (1|subj), data = copv)
m2 = lmerTest::lmer(dx2 ~ 1 + Vibration + Vision + (1|subj), data = copv)
# m3 = lmerTest::lmer(dx2 ~ 1 + Vibration * Vision + (1|subj), data = copv)
# m3 = lmerTest::lmer(dx2 ~ 1 + TCThresholdGroup + Vibration + Vision + (1|subj), data = copv)
# m4 = lmerTest::lmer(dx2 ~ 1 + Cause + Vibration + Vision + (1|subj), data = copv)
#m5 = lmerTest::lmer(dx2 ~ 1 + zMass + Vibration + Vision + (1|subj), data = copv)
#m6 = lmerTest::lmer(dx2 ~ 1 + zMass + zMass2 + Vibration + Vision + (1|subj), data = copv)
#m7 = lmerTest::lmer(dx2 ~ 1 + Vibration*zMass2 + Vision*zMass2 + (1|subj), data = copv)
anova(m0, m1, m2)
```

```
## refitting model(s) with ML (instead of REML)
```

```
## Data: copv
## Models:
## m0: dx2 ~ 1 + (1 | subj)
## m1: dx2 ~ 1 + Vibration + (1 | subj)
## m2: dx2 ~ 1 + Vibration + Vision + (1 | subj)
##    npar     AIC     BIC logLik deviance   Chisq Df Pr(>Chisq)
## m0    3 -1825.5 -1817.3 915.76  -1831.5
## m1    5 -1823.6 -1809.9 916.79  -1833.6  2.0613  2     0.3568
## m2    6 -1855.4 -1839.0 933.71  -1867.4 33.8342  1  6.002e-09 ***
## ---
## Signif. codes:  0 '***' 0.001 '**' 0.01 '*' 0.05 '.' 0.1 ' ' 1
```

```r
print(summary(m2))
```

```
## Linear mixed model fit by REML. t-tests use Satterthwaite's method [
## lmerModLmerTest]
## Formula: dx2 ~ 1 + Vibration + Vision + (1 | subj)
##    Data: copv
##
## REML criterion at convergence: -1784.6
##
## Scaled residuals:
##     Min      1Q  Median      3Q     Max
## -2.5222 -0.3973  0.0368  0.2910  7.0826
##
## Random effects:
##  Groups   Name        Variance  Std.Dev.
##  subj     (Intercept) 7.465e-09 8.640e-05
##  Residual             2.931e-09 5.414e-05
## Number of obs: 114, groups:  subj, 19
##
## Fixed effects:
##              Estimate Std. Error        df t value Pr(>|t|)
## (Intercept) 4.967e-05  2.227e-05 2.508e+01   2.231   0.0349 *
## Vibration2  9.204e-06  1.242e-05 9.200e+01   0.741   0.4605
## Vibration3  2.103e-05  1.242e-05 9.200e+01   1.693   0.0938 .
## Vision2     6.362e-05  1.014e-05 9.200e+01   6.274 1.14e-08 ***
```

```
## ---
## Signif. codes:  0 '***' 0.001 '**' 0.01 '*' 0.05 '.' 0.1 ' ' 1
##
## Correlation of Fixed Effects:
##             (Intr) Vbrtn2 Vbrtn3
## Vibration2 -0.279
## Vibration3 -0.279  0.500
## Vision2    -0.228  0.000  0.000
```

```
g17 = ggplot(copv, aes(Vision, dx2, colour = Vibration, fill = Vibration)) +
  stat_summary(fun.data = 'mean_cl_boot', position = position_dodge(),geom = 'bar') +
  stat_summary(fun.data = 'mean_cl_boot', position = position_dodge(0.9), geom = 'errorbar', colour = 'l
print(g17)
```

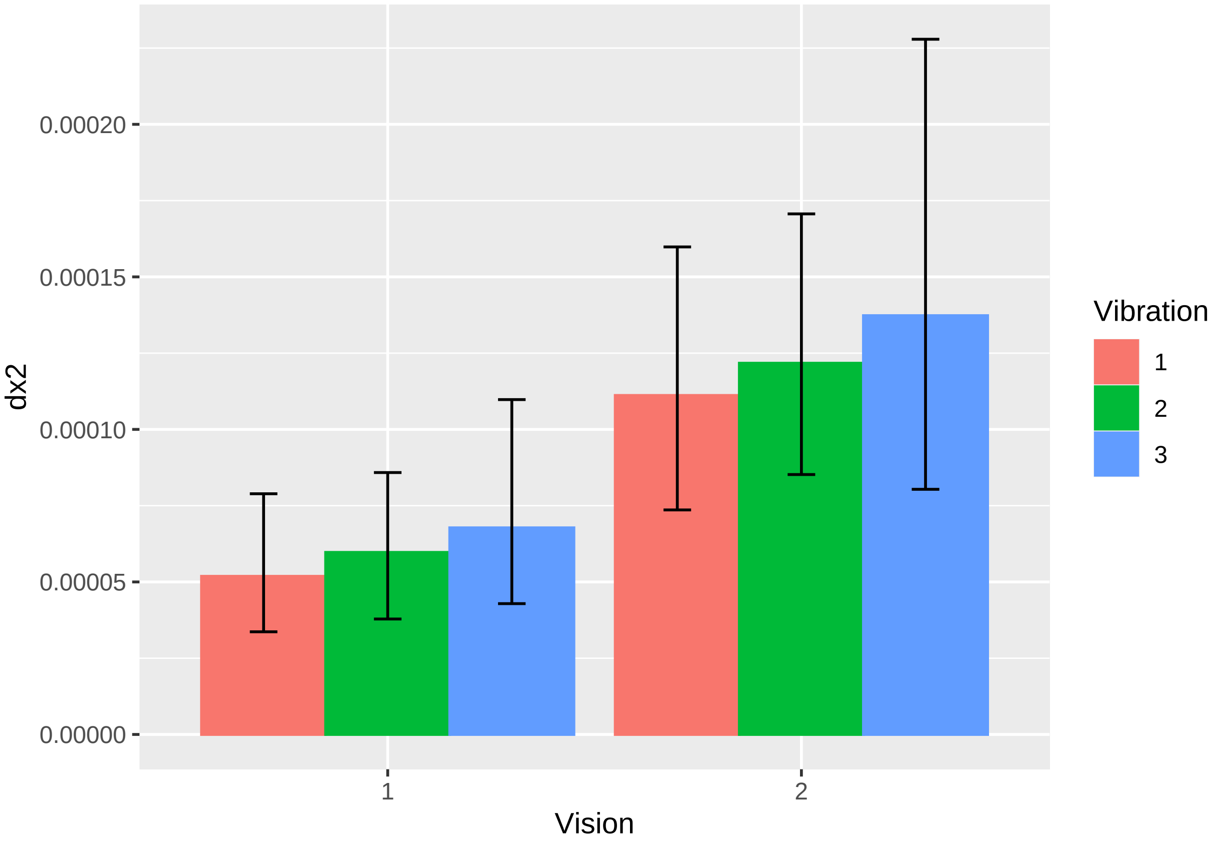


```
# dr2 ------------------------------------------------------------------

m0 = lmerTest::lmer(dr2 ~ 1 + (1|subj), data = copv)
m1 = lmerTest::lmer(dr2 ~ 1 + Vibration + (1|subj), data = copv)
m2 = lmerTest::lmer(dr2 ~ 1 + Vibration + Vision + (1|subj), data = copv)
# # m3 = lmerTest::lmer(dr2 ~ 1 + Vibration * Vision + (1|subj), data = copv)
# # m3 = lmerTest::lmer(dr2 ~ 1 + TCThresholdGroup + Vibration + Vision + (1|subj), data = copv)
# # m4 = lmerTest::lmer(dr2 ~ 1 + Cause + Vibration + Vision + (1|subj), data = copv)
# m5 = lmerTest::lmer(dr2 ~ 1 + zMass + Vibration + Vision + (1|subj), data = copv)
# m6 = lmerTest::lmer(dr2 ~ 1 + zMass + zMass2 + Vibration + Vision + (1|subj), data = copv)
# m7 = lmerTest::lmer(dr2 ~ 1 + Vibration*zMass2 + Vision*zMass2 + (1|subj), data = copv)
anova(m0, m1, m2)
```

```
## refitting model(s) with ML (instead of REML)
```

```
## Data: copv
## Models:
## m0: dr2 ~ 1 + (1 | subj)
## m1: dr2 ~ 1 + Vibration + (1 | subj)
## m2: dr2 ~ 1 + Vibration + Vision + (1 | subj)
##    npar     AIC     BIC logLik deviance   Chisq Df Pr(>Chisq)
## m0    3 -1606.2 -1598.0 806.11  -1612.2
## m1    5 -1604.1 -1590.4 807.03  -1614.1  1.8409  2     0.3983
## m2    6 -1620.5 -1604.1 816.25  -1632.5 18.4409  1  1.753e-05 ***
## ---
## Signif. codes:  0 '***' 0.001 '**' 0.01 '*' 0.05 '.' 0.1 ' ' 1
```

```r
print(summary(m2))
```

```
## Linear mixed model fit by REML. t-tests use Satterthwaite's method [
## lmerModLmerTest]
## Formula: dr2 ~ 1 + Vibration + Vision + (1 | subj)
##    Data: copv
##
## REML criterion at convergence: -1558
##
## Scaled residuals:
##     Min      1Q  Median      3Q     Max
## -3.2220 -0.3074 -0.0145  0.2060  6.8651
##
## Random effects:
##  Groups   Name        Variance  Std.Dev.
##  subj     (Intercept) 5.911e-08 0.0002431
##  Residual             2.297e-08 0.0001516
## Number of obs: 114, groups:  subj, 19
##
## Fixed effects:
##              Estimate Std. Error        df t value Pr(>|t|)
## (Intercept) 9.261e-05  6.259e-05 2.501e+01   1.480    0.151
## Vibration2  3.959e-05  3.477e-05 9.200e+01   1.138    0.258
## Vibration3  4.820e-05  3.477e-05 9.200e+01   1.386    0.169
## Vision2     1.261e-04  2.839e-05 9.200e+01   4.440  2.5e-05 ***
## ---
## Signif. codes:  0 '***' 0.001 '**' 0.01 '*' 0.05 '.' 0.1 ' ' 1
##
## Correlation of Fixed Effects:
##            (Intr) Vbrtn2 Vbrtn3
## Vibration2 -0.278
## Vibration3 -0.278  0.500
## Vision2    -0.227  0.000  0.000
```

```r
g18 = ggplot(copv, aes(Vision, dr2, colour = Vision, fill = Vibration)) +
  stat_summary(fun.data = 'mean_cl_boot', position = position_dodge(),geom = 'bar') +
  stat_summary(fun.data = 'mean_cl_boot', position = position_dodge(0.9), geom = 'errorbar', colour = 'l
print(g18)
```

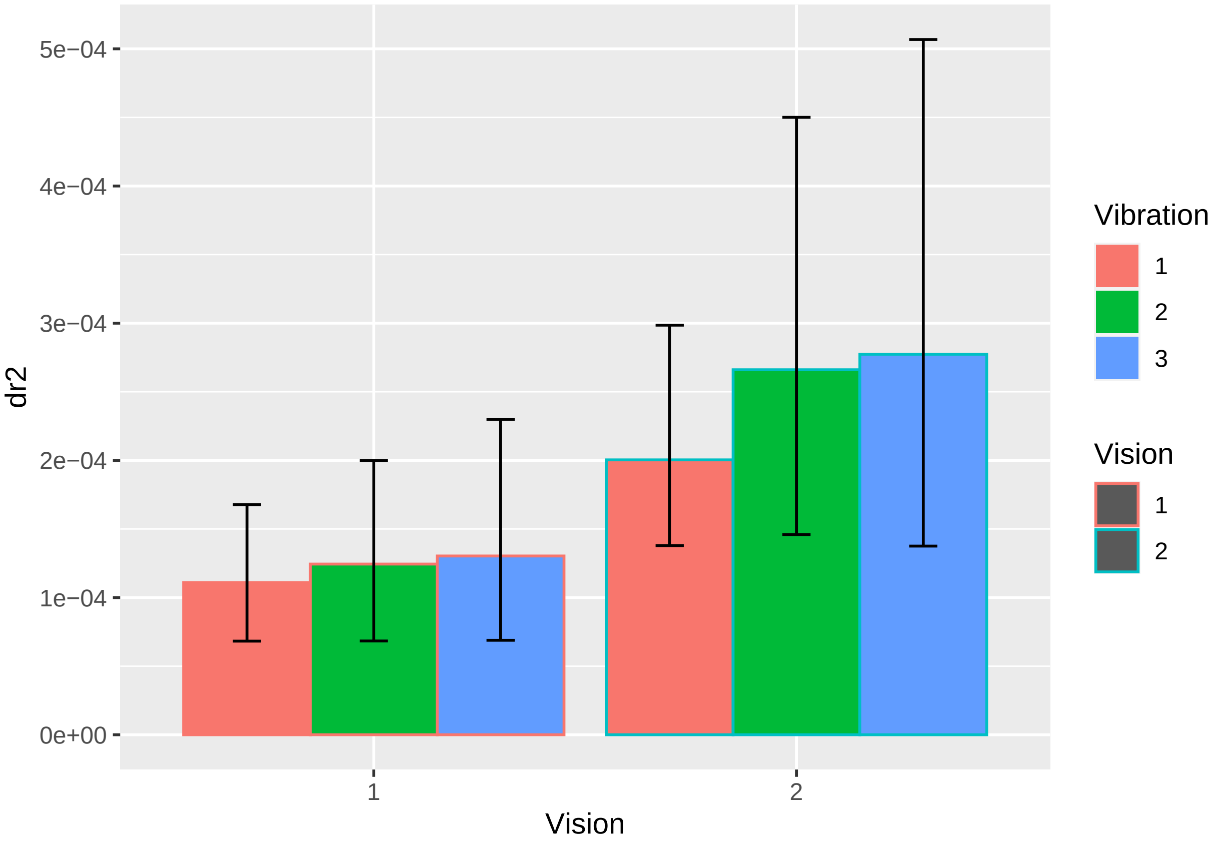
5e−04
4e−04
3e−04
2e−04
1e−04
0e+00
dr2
1
2
Vision
Vibration
1
2
3
Vision
1
2
