# Supplementary material for: Subthreshold Vibration Influences Standing Balance but Has Unclear Impact on Somatosensation in Persons With Transtibial Amputations
Source: Front Physiol. 2022 Feb 2;13:810079. doi: 10.3389/fphys.2022.810079 (PMC8847287; doi:10.3389/fphys.2022.810079)

basic_models_y_no_filter.R

alikens

2020-07-06

```
library(lmerTest)
```

```
## Loading required package: lme4

## Loading required package: Matrix

##
## Attaching package: 'lmerTest'

## The following object is masked from 'package:lme4':
##
##     lmer

## The following object is masked from 'package:stats':
##
##     step
```

```
library(ggplot2)
library(sjPlot)
```

```
## Learn more about sjPlot with 'browseVignettes("sjPlot")'.
```

```
# look at differences in threshold
# this may need to use different distributions
# predict clusters in perception variables from the type of amputaion, age, weight

# read in the data
sense = read.csv("./data/Sensation_Data_Reorganized.csv")
copv = read.delim("./data/Amputee_COP_VAC_100HZ_summary_noFilter.txt")
# copv = copv[,!grepl('H', names(copv))]
join_sense = c('subj', 'Vibration', 'CondNum')
join_copv = c('subj', 'Vibration', 'CondNum')
copv = merge(copv,sense, by.x = join_copv, by.y = join_sense)
copv$Vision = as.factor(copv$Vision)
copv$Vibration = as.factor(copv$Vibration)
copv = copv[copv$Vision != 0,]
copv$Mass2 = copv$Mass^2
zMass = as.numeric(scale(copv$Mass))
copv$zMass = zMass
copv$zMass2 = I(zMass^2)

# model for zeroTime
m0 = lmerTest::lmer(zeroTimeY ~ 1 + (1|subj), data = copv)
m1 = lmerTest::lmer(zeroTimeY ~ 1 + Vibration + (1|subj), data = copv)
m2 = lmerTest::lmer(zeroTimeY ~ 1 + Vibration + Vision + (1|subj), data = copv)
m3 = lmerTest::lmer(zeroTimeY ~ 1 + Vibration * Vision + (1|subj), data = copv)
```

```r
# m3 = lmerTest::lmer(zeroTimeY ~ 1 + Cause + Vision + Vibration + (1|subj), data = copv)
# m3 = lmerTest::lmer(zeroTimeY ~ 1 + TCThresholdGroup + Vision + Vibration + (1|subj), data = copv)
m4 = lmerTest::lmer(zeroTimeY ~ 1 + Vibration * Vision + Mass + (1|subj), data = copv)
m5 = lmerTest::lmer(zeroTimeY ~ 1 + Vibration + Vision + Mass + Mass:Vibration + (1|subj), data = copv)
# m6 = lmerTest::lmer(zeroTimeY ~ 1 + Mass * Vision * Vibration + (1|subj), data = copv)

# maybe we should also look at BMI
anova(m0, m1, m2, m3, m4, m5)
```

```
## refitting model(s) with ML (instead of REML)
```

```
## Data: copv
## Models:
## m0: zeroTimeY ~ 1 + (1 | subj)
## m1: zeroTimeY ~ 1 + Vibration + (1 | subj)
## m2: zeroTimeY ~ 1 + Vibration + Vision + (1 | subj)
## m3: zeroTimeY ~ 1 + Vibration * Vision + (1 | subj)
## m4: zeroTimeY ~ 1 + Vibration * Vision + Mass + (1 | subj)
## m5: zeroTimeY ~ 1 + Vibration + Vision + Mass + Mass:Vibration +
## m5:     (1 | subj)
##    npar     AIC     BIC  logLik deviance   Chisq Df Pr(>Chisq)
## m0    3 -169.68 -161.47  87.840  -175.68
## m1    5 -167.03 -153.35  88.516  -177.03  1.3518  2    0.50871
## m2    6 -180.74 -164.32  96.369  -192.74 15.7063  1  7.398e-05 ***
## m3    8 -182.90 -161.01  99.449  -198.90  6.1613  2    0.04593 *
## m4    9 -187.42 -162.80 102.711  -205.42  6.5227  1    0.01065 *
## m5    9 -187.97 -163.34 102.986  -205.97  0.5498  0  < 2.2e-16 ***
## ---
## Signif. codes:  0 '***' 0.001 '**' 0.01 '*' 0.05 '.' 0.1 ' ' 1
```

```r
print(summary(m5))
```

```
## Linear mixed model fit by REML. t-tests use Satterthwaite's method [
## lmerModLmerTest]
## Formula: zeroTimeY ~ 1 + Vibration + Vision + Mass + Mass:Vibration +
##     (1 | subj)
##    Data: copv
##
## REML criterion at convergence: -146.2
##
## Scaled residuals:
##     Min      1Q  Median      3Q     Max
## -2.0059 -0.6308  0.0679  0.5706  4.2627
##
## Random effects:
##  Groups   Name        Variance Std.Dev.
##  subj     (Intercept) 0.003068 0.05539
##  Residual             0.008448 0.09191
## Number of obs: 114, groups:  subj, 19
##
## Fixed effects:
##                  Estimate Std. Error        df t value Pr(>|t|)
## (Intercept)     -0.343041   0.136888 42.255352  -2.506 0.016146 *
## Vibration2       0.371976   0.147050 90.000001   2.530 0.013160 *
## Vibration3       0.277977   0.147050 90.000001   1.890 0.061929 .
```

```
## Vision2          0.071746   0.017217 90.000000   4.167 7.07e-05 ***
## Mass             0.004895   0.001347 41.982065   3.636 0.000751 ***
## Vibration2:Mass -0.003507   0.001449 90.000001  -2.420 0.017533 *
## Vibration3:Mass -0.002827   0.001449 90.000001  -1.951 0.054190 .
## ---
## Signif. codes:  0 '***' 0.001 '**' 0.01 '*' 0.05 '.' 0.1 ' ' 1
##
## Correlation of Fixed Effects:
##              (Intr) Vbrtn2 Vbrtn3 Visin2 Mass   Vbr2:M
## Vibration2  -0.537
## Vibration3  -0.537  0.500
## Vision2     -0.063  0.000  0.000
## Mass        -0.988  0.533  0.533  0.000
## Vibrtn2:Mss  0.532 -0.990 -0.495  0.000 -0.538
## Vibrtn3:Mss  0.532 -0.495 -0.990  0.000 -0.538  0.500
```

```
sjPlot::plot_model(model = m5, type = 'int')
```

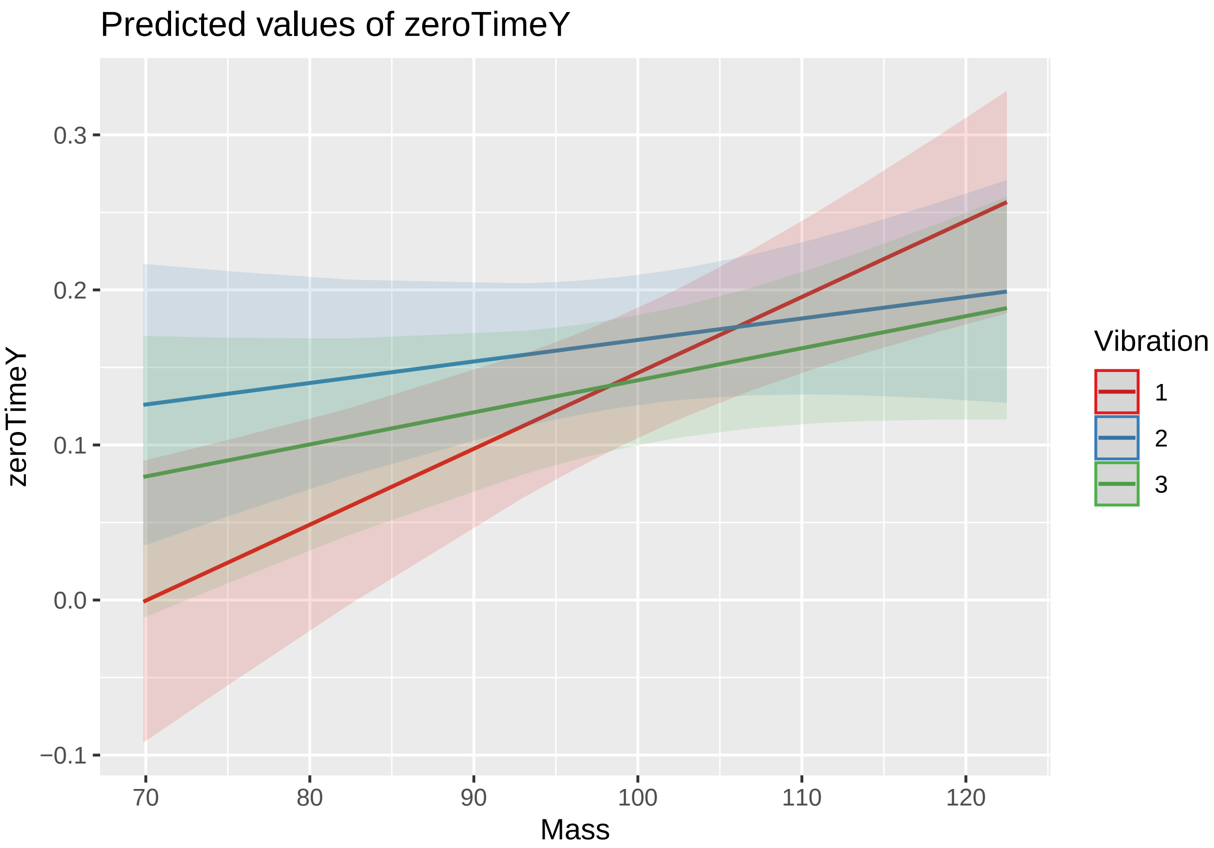


```
# reghelper::simple_slopes(m5)

# visualize Vision by Vibration Interaction
g1 = ggplot(copv, aes(Vibration, zeroTimeY, colour = Vision, fill = Vision)) +
  stat_summary(fun.data = 'mean_cl_boot', position = position_dodge(),geom = 'bar') +
  stat_summary(fun.data = 'mean_cl_boot', position = position_dodge(0.9), geom = 'errorbar', colour = 'l
print(g1)
```

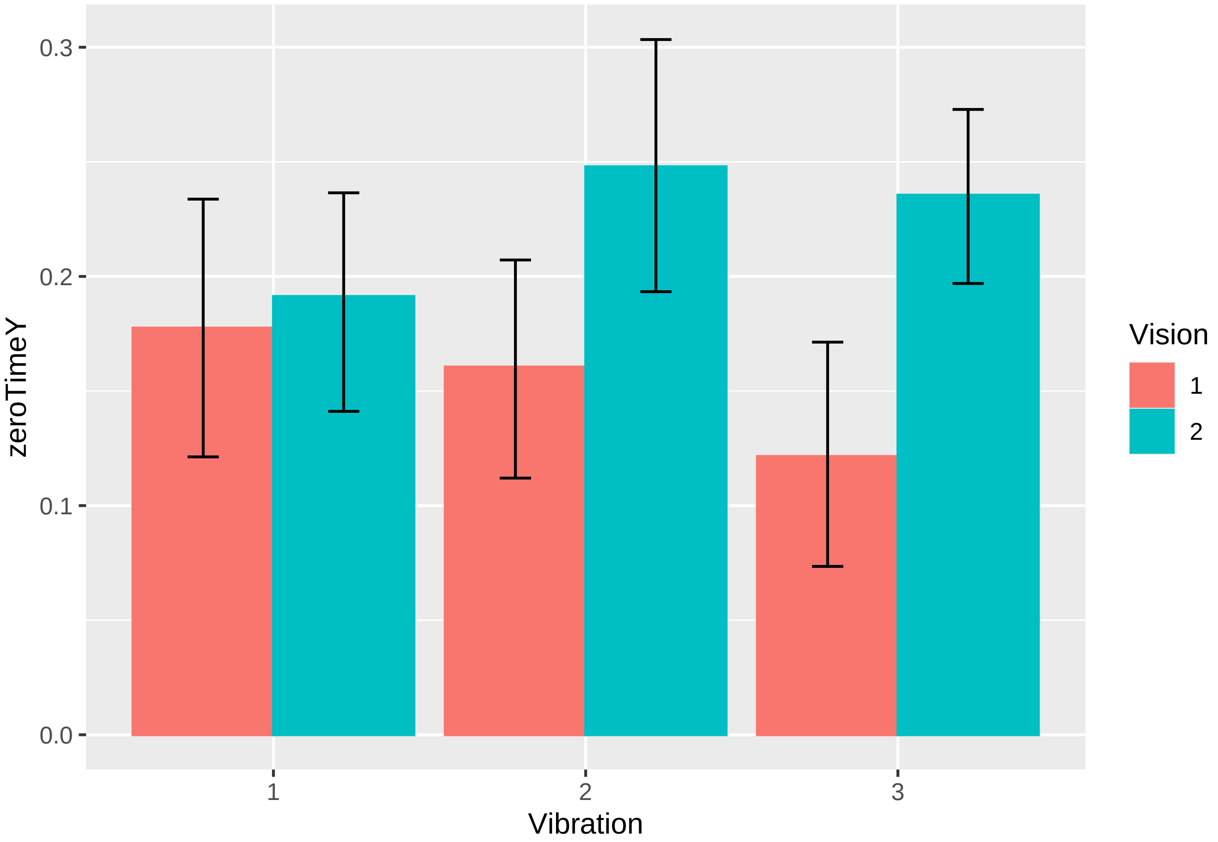


```
# visualize Mass by Vibration Interaction


# model for minTime
m0 = lmerTest::lmer(minTimeY ~ 1 + ( 1|subj ), data = copv)
m1 = lmerTest::lmer(minTimeY ~ 1 + Vibration + (1|subj), data = copv)
m2 = lmerTest::lmer(minTimeY ~ 1 + Vibration + Vision + (1|subj), data = copv)
m3 = lmerTest::lmer(minTimeY ~ 1 + Vibration * Vision + (1|subj), data = copv)
# m4 = lmerTest::lmer(minTimeY ~ 1 + Cause + Vision * Vibration + (1|subj), data = copv)
# m4 = lmerTest::lmer(minTimeY ~ 1 + TCThresholdGroup + Vision * Vibration + (1|subj), data = copv)
m4 = lmerTest::lmer(minTimeY ~ 1 + Vibration * Vision + Mass + (1|subj), data = copv)
# m5 = lmerTest::lmer(minTimeY ~ 1 + Vibration * Vision + Mass + TCThresholdGroup + (1|subj), data = co
# m5 = lmerTest::lmer(minTimeY ~ 1 + Vibration * Vision + Mass + Mass:Vibration + (1|subj), data = copv
# m5 = lmerTest::lmer(minTimeY ~ 1 + Vibration * Vision + Mass + Mass:Vision + Mass:Vision +(1|subj), d
# m6 = lmerTest::lmer(minTimeY ~ 1 + Mass * Vision * Vibration + (1|subj), data = copv)

anova(m0, m1, m2, m3, m4, m5)
```

```
## refitting model(s) with ML (instead of REML)
```

```
## Data: copv
## Models:
## m0: minTimeY ~ 1 + (1 | subj)
## m1: minTimeY ~ 1 + Vibration + (1 | subj)
## m2: minTimeY ~ 1 + Vibration + Vision + (1 | subj)
## m3: minTimeY ~ 1 + Vibration * Vision + (1 | subj)
## m4: minTimeY ~ 1 + Vibration * Vision + Mass + (1 | subj)
## m5: zeroTimeY ~ 1 + Vibration + Vision + Mass + Mass:Vibration +
```

```
## m5:     (1 | subj)
##    npar      AIC      BIC  logLik deviance    Chisq Df Pr(>Chisq)
## m0    3    7.137   15.346  -0.569    1.137
## m1    5    9.177   22.858   0.412   -0.823   1.9608  2  0.3751596
## m2    6   -1.918   14.499   6.959  -13.918  13.0944  1  0.0002962 ***
## m3    8   -4.160   17.730  10.080  -20.160   6.2418  2  0.0441165 *
## m4    9   -9.284   15.342  13.642  -27.284   7.1239  1  0.0076063 **
## m5    9 -187.971 -163.345 102.986 -205.971 178.6877  0  < 2.2e-16 ***
## ---
## Signif. codes:  0 '***' 0.001 '**' 0.01 '*' 0.05 '.' 0.1 ' ' 1
```

```
print(summary(m4))
```

```
## Linear mixed model fit by REML. t-tests use Satterthwaite's method [
## lmerModLmerTest]
## Formula: minTimeY ~ 1 + Vibration * Vision + Mass + (1 | subj)
##    Data: copv
##
## REML criterion at convergence: 8.2
##
## Scaled residuals:
##     Min      1Q  Median      3Q     Max
## -1.9674 -0.6079 -0.0527  0.3859  5.2765
##
## Random effects:
##  Groups   Name        Variance Std.Dev.
##  subj     (Intercept) 0.01014  0.1007
##  Residual             0.04234  0.2058
## Number of obs: 114, groups:  subj, 19
##
## Fixed effects:
##                      Estimate Std. Error        df t value Pr(>|t|)
## (Intercept)         -0.238400   0.214160 18.458759  -1.113   0.2799
## Vibration2          -0.018947   0.066759 90.000000  -0.284   0.7772
## Vibration3          -0.135789   0.066759 90.000000  -2.034   0.0449 *
## Vision2              0.022105   0.066759 90.000000   0.331   0.7413
## Mass                 0.005750   0.002068 17.000000   2.781   0.0128 *
## Vibration2:Vision2   0.137368   0.094411 90.000000   1.455   0.1491
## Vibration3:Vision2   0.232105   0.094411 90.000000   2.458   0.0159 *
## ---
## Signif. codes:  0 '***' 0.001 '**' 0.01 '*' 0.05 '.' 0.1 ' ' 1
##
## Correlation of Fixed Effects:
##             (Intr) Vbrtn2 Vbrtn3 Visin2 Mass   Vb2:V2
## Vibration2  -0.156
## Vibration3  -0.156  0.500
## Vision2     -0.156  0.500  0.500
## Mass        -0.969  0.000  0.000  0.000
## Vbrtn2:Vsn2  0.110 -0.707 -0.354 -0.707  0.000
## Vbrtn3:Vsn2  0.110 -0.354 -0.707 -0.707  0.000  0.500
```

```
sjPlot::plot_model(m4, type = 'int')
```

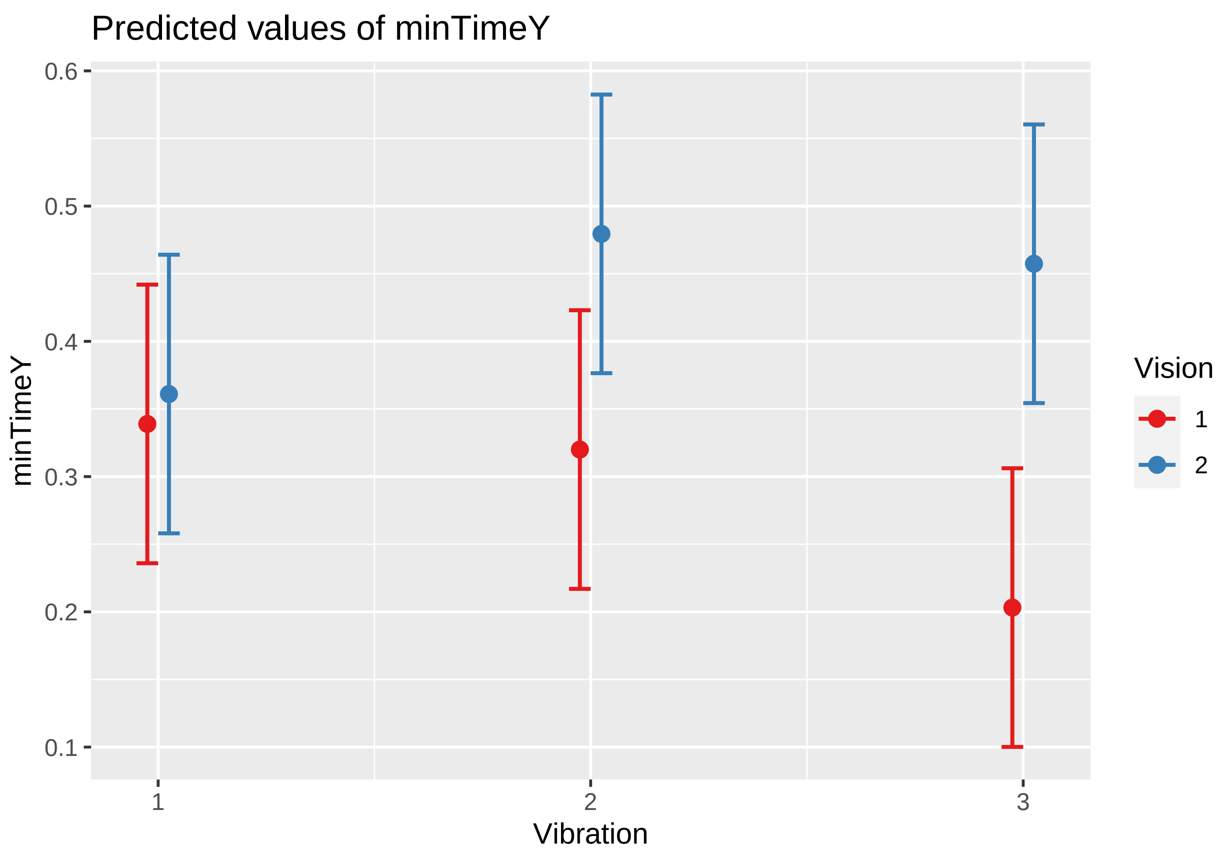


```
g2 = ggplot(copv, aes(Vibration, minTimeY, colour = Vision, fill = Vision)) +
  stat_summary(fun.data = 'mean_cl_boot', position = position_dodge(),geom = 'bar') +
  stat_summary(fun.data = 'mean_cl_boot', position = position_dodge(0.9), geom = 'errorbar', colour = 'I
print(g2)
```

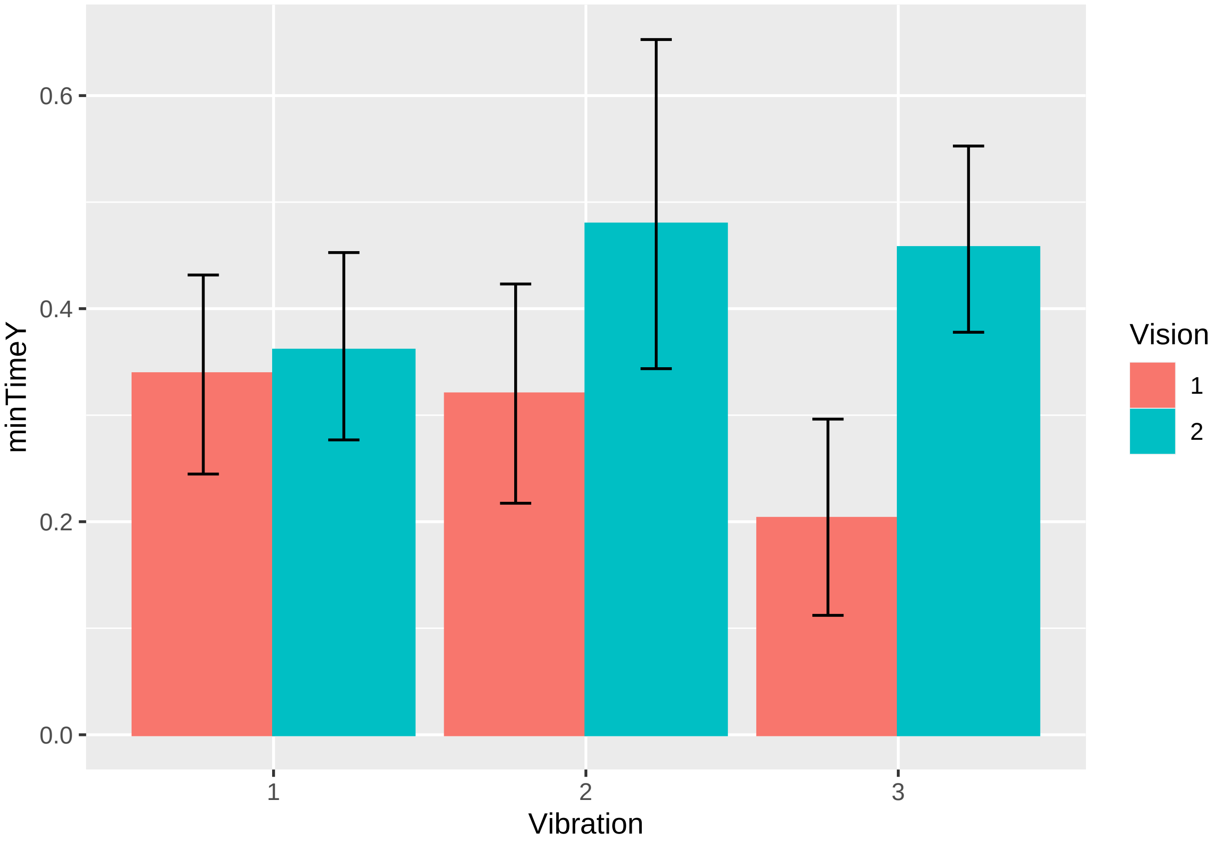


```
# model for minTValY
m0 = lmerTest::lmer(minValY ~ 1 + (1|subj), data = copv)
m1 = lmerTest::lmer(minValY ~ 1 + Vibration + (1|subj), data = copv)
m2 = lmerTest::lmer(minValY ~ 1 + Vibration + Vision + (1|subj), data = copv)
# m3 = lmerTest::lmer(minValY ~ 1 + Vibration * Vision + (1|subj), data = copv)
# m3 = lmerTest::lmer(minValY ~ 1 + Cause + Vision + Vibration + (1|subj), data = copv)
# m4 = lmerTest::lmer(minValY ~ 1 + TCThresholdGroup + Vision + Vibration + (1|subj), data = copv)
# m4 = lmerTest::lmer(minValY ~ 1 + Vibration + Vision + Mass + (1|subj), data = copv)
# m5 = lmerTest::lmer(minValY ~ 1 + Vibration * Vision + Mass + Mass:Vibration + (1|subj), data = copv)
# m6 = lmerTest::lmer(minValY ~ 1 + Vibration * Vision + Mass + Mass:Vibration + Mass:Vision +(1|subj),
# m6 = lmerTest::lmer(minValY ~ 1 + Mass * Vision * Vibration + (1|subj), data = copv)

anova(m0, m1, m2)
```

```
## refitting model(s) with ML (instead of REML)
```

```
## Data: copv
## Models:
## m0: minValY ~ 1 + (1 | subj)
## m1: minValY ~ 1 + Vibration + (1 | subj)
## m2: minValY ~ 1 + Vibration + Vision + (1 | subj)
##    npar     AIC     BIC logLik deviance  Chisq Df Pr(>Chisq)
## m0    3 -1607.7 -1599.5 806.86  -1613.7
## m1    5 -1604.9 -1591.2 807.43  -1614.9  1.134  2  0.5672255
## m2    6 -1616.9 -1600.5 814.45  -1628.9 14.038  1  0.0001792 ***
## ---
## Signif. codes:  0 '***' 0.001 '**' 0.01 '*' 0.05 '.' 0.1 ' ' 1
```

```r
print(summary(m2))
```

```
## Linear mixed model fit by REML. t-tests use Satterthwaite's method [
## lmerModLmerTest]
## Formula: minValY ~ 1 + Vibration + Vision + (1 | subj)
##    Data: copv
##
## REML criterion at convergence: -1554.3
##
## Scaled residuals:
##     Min      1Q  Median      3Q     Max
## -5.5432 -0.2775  0.1096  0.4547  2.4996
##
## Random effects:
##  Groups   Name        Variance  Std.Dev.
##  subj     (Intercept) 3.589e-08 0.0001895
##  Residual             2.610e-08 0.0001615
## Number of obs: 114, groups:  subj, 19
##
## Fixed effects:
##               Estimate Std. Error         df t value Pr(>|t|)
## (Intercept) -1.137e-04  5.296e-05  3.093e+01  -2.148 0.039681 *
## Vibration2  -4.149e-05  3.706e-05  9.200e+01  -1.120 0.265797
## Vibration3  -1.546e-05  3.706e-05  9.200e+01  -0.417 0.677619
## Vision2     -1.158e-04  3.026e-05  9.200e+01  -3.828 0.000236 ***
## ---
## Signif. codes:  0 '***' 0.001 '**' 0.01 '*' 0.05 '.' 0.1 ' ' 1
##
## Correlation of Fixed Effects:
##            (Intr) Vbrtn2 Vbrtn3
## Vibration2 -0.350
## Vibration3 -0.350  0.500
## Vision2    -0.286  0.000  0.000
```

```r
g3 = ggplot(copv, aes(Vision, minValY, colour = Vision, fill = Vision)) +
  stat_summary(fun.data = 'mean_cl_boot', position = position_dodge(),geom = 'bar') +
  stat_summary(fun.data = 'mean_cl_boot', position = position_dodge(0.9), geom = 'errorbar', colour = '
print(g3)
```

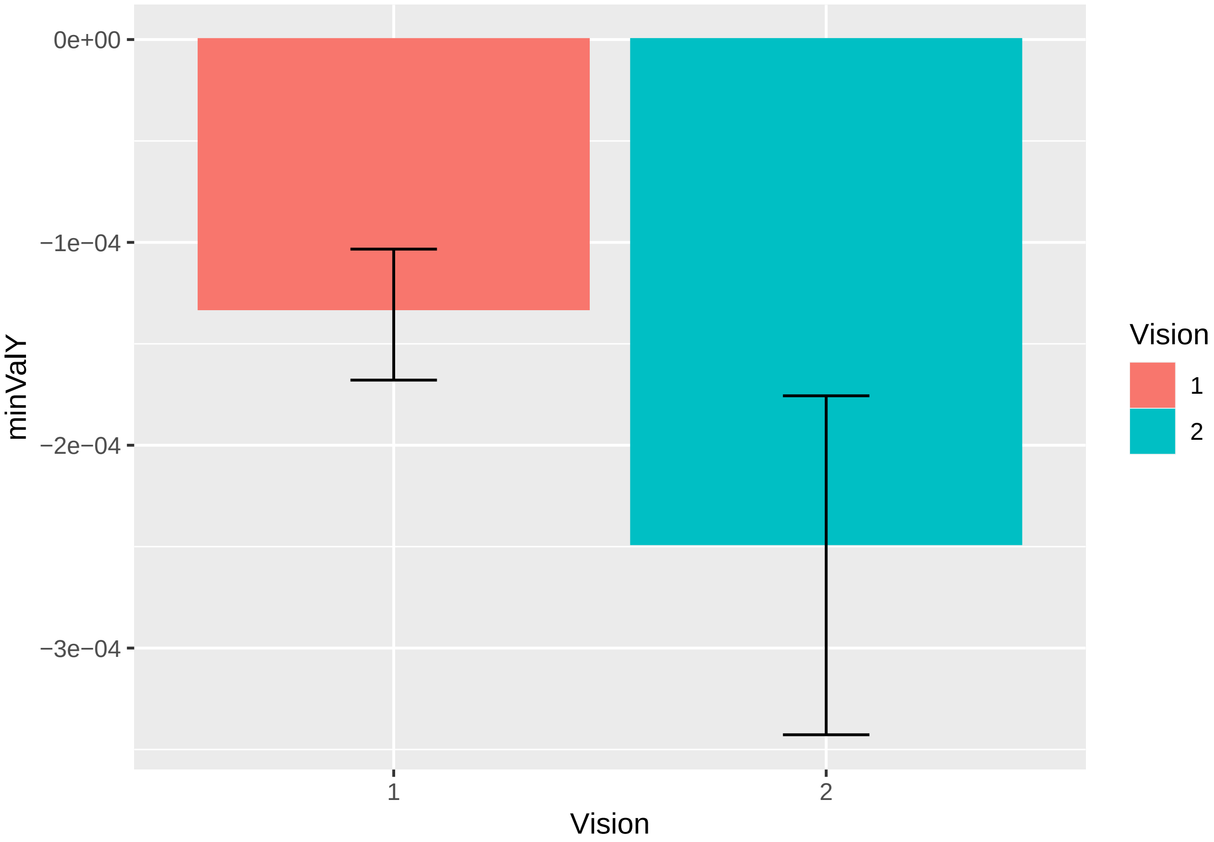


```
# model for CvOY
m0 = lmerTest::lmer(CvOY ~ 1 + ( 1|subj ), data = copv)
m1 = lmerTest::lmer(CvOY ~ 1 + Vibration + (1|subj), data = copv)
m2 = lmerTest::lmer(CvOY ~ 1 + Vibration + Vision + (1|subj), data = copv)
# m3 = lmerTest::lmer(CvOY ~ 1 + Vibration * Vision + (1|subj), data = copv)
# m3 = lmerTest::lmer(CvOY ~ 1 + Cause + Vision + Vibration + (1|subj), data = copv)
# m3 = lmerTest::lmer(CvOY ~ 1 + TCThresholdGroup + Vision + Vibration + (1|subj), data = copv)
# m3 = lmerTest::lmer(CvOY ~ 1 + Vibration + Vision + Mass + (1|subj), data = copv)
# m5 = lmerTest::lmer(CvOY ~ 1 + Vibration * Vision + Mass + TCThresholdGroup + (1|subj), data = copv)
# m5 = lmerTest::lmer(CvOY ~ 1 + Vibration * Vision + Mass + Mass:Vibration + (1|subj), data = copv)
# m6 = lmerTest::lmer(CvOY ~ 1 + Vibration * Vision + Mass + Mass:Vibration + Mass:Vision +(1|subj), da
# m6 = lmerTest::lmer(CvOY ~ 1 + Mass * Vision * Vibration + (1|subj), data = copv)

anova(m0, m1, m2)
```

```
## refitting model(s) with ML (instead of REML)
```

```
## Data: copv
## Models:
## m0: CvOY ~ 1 + (1 | subj)
## m1: CvOY ~ 1 + Vibration + (1 | subj)
## m2: CvOY ~ 1 + Vibration + Vision + (1 | subj)
##    npar     AIC     BIC logLik deviance   Chisq Df Pr(>Chisq)
## m0    3 -1298.5 -1290.2 652.23  -1304.5
## m1    5 -1296.8 -1283.1 653.39  -1306.8  2.3134  2     0.3145
## m2    6 -1319.8 -1303.3 665.88  -1331.8 24.9849  1  5.778e-07 ***
## ---
## Signif. codes:  0 '***' 0.001 '**' 0.01 '*' 0.05 '.' 0.1 ' ' 1
```

```r
print(summary(m2))
```

```
## Linear mixed model fit by REML. t-tests use Satterthwaite's method [
## lmerModLmerTest]
## Formula: CvOY ~ 1 + Vibration + Vision + (1 | subj)
##    Data: copv
##
## REML criterion at convergence: -1267.8
##
## Scaled residuals:
##     Min      1Q  Median      3Q     Max
## -3.5049 -0.3979 -0.0011  0.2159  4.7341
##
## Random effects:
##  Groups   Name        Variance  Std.Dev.
##  subj     (Intercept) 8.907e-07 0.0009437
##  Residual             3.169e-07 0.0005629
## Number of obs: 114, groups:  subj, 19
##
## Fixed effects:
##              Estimate Std. Error        df t value Pr(>|t|)
## (Intercept) 6.185e-04  2.408e-04 2.442e+01   2.568   0.0167 *
## Vibration2  2.211e-04  1.291e-04 9.200e+01   1.712   0.0903 .
## Vibration3  1.262e-04  1.291e-04 9.200e+01   0.977   0.3312
## Vision2     5.547e-04  1.054e-04 9.200e+01   5.261  9.3e-07 ***
## ---
## Signif. codes:  0 '***' 0.001 '**' 0.01 '*' 0.05 '.' 0.1 ' ' 1
##
## Correlation of Fixed Effects:
##            (Intr) Vbrtn2 Vbrtn3
## Vibration2 -0.268
## Vibration3 -0.268  0.500
## Vision2    -0.219  0.000  0.000
```

```r
g4 = ggplot(copv, aes(Vision, CvOY, colour = Vision, fill = Vision)) +
  stat_summary(fun.data = 'mean_cl_boot', position = position_dodge(),geom = 'bar') +
  stat_summary(fun.data = 'mean_cl_boot', position = position_dodge(0.9), geom = 'errorbar', colour = '[illegible]
print(g4)
```

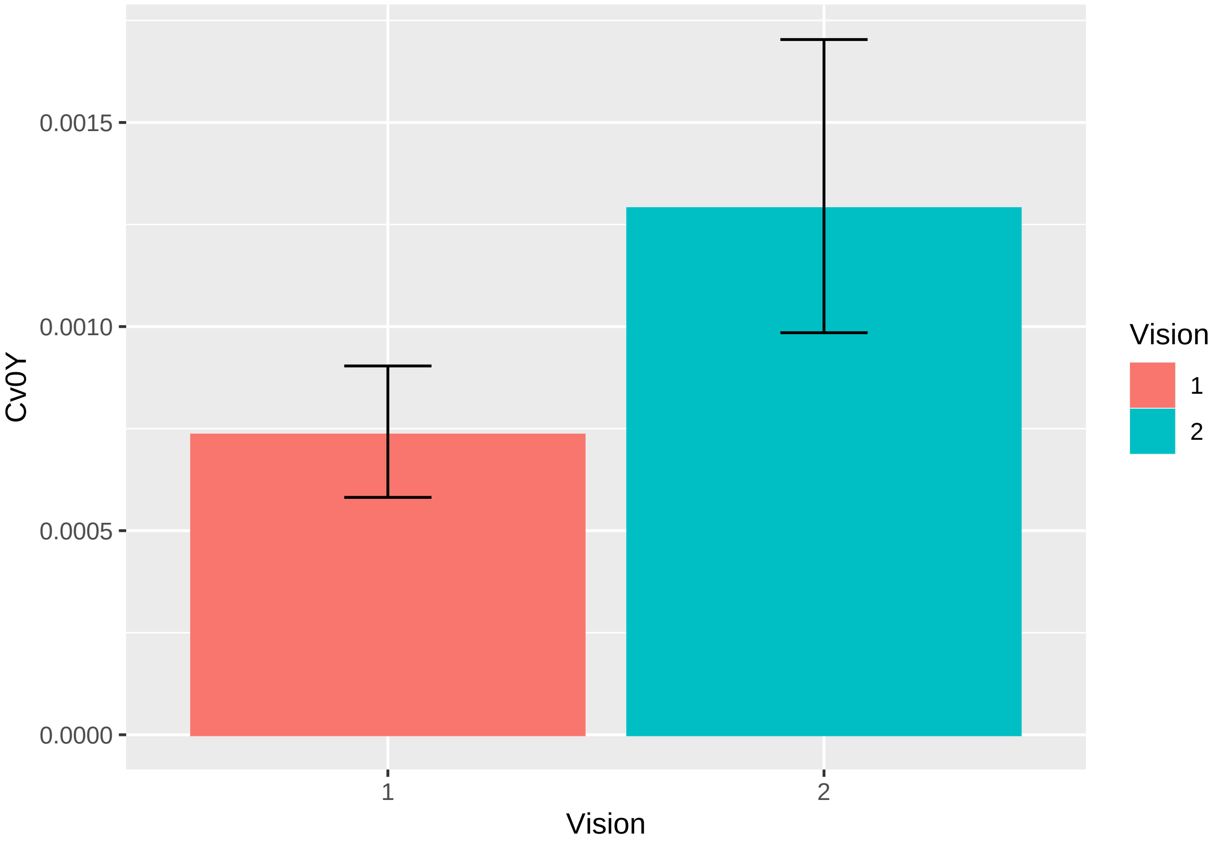


```
# model for diffusion coefficient = singular fit for all models
# m0 = lmerTest::lmer(DiffusionCoefY ~ 1 + ( 1|subj ), data = copv)
# m1 = lmerTest::lmer(DiffusionCoefY ~ 1 + Vibration + (1|subj), data = copv)
# m2 = lmerTest::lmer(DiffusionCoefY ~ 1 + Vibration + Vision + (1|subj), data = copv)
# m3 = lmerTest::lmer(DiffusionCoefY ~ 1 + Vibration * Vision + (1|subj), data = copv)
# # m4 = lmerTest::lmer(DiffusionCoefY ~ 1 + Cause + Vision * Vibration + (1|subj), data = copv)
# # m4 = lmerTest::lmer(DiffusionCoefY ~ 1 + TCThresholdGroup + Vision * Vibration + (1|subj), data = c
# m4 = lmerTest::lmer(DiffusionCoefY ~ 1 + Vibration * Vision + Mass + (1|subj), data = copv)
# # m5 = lmerTest::lmer(DiffusionCoefY ~ 1 + Vibration * Vision + Mass + TCThresholdGroup + (1|subj), d
# # m5 = lmerTest::lmer(DiffusionCoefY ~ 1 + Vibration * Vision + Mass + Mass:Vibration + (1|subj), dat
# # m6 = lmerTest::lmer(DiffusionCoefY ~ 1 + Vibration * Vision + Mass + Mass:Vibration + Mass:Vision +
# # m6 = lmerTest::lmer(DiffusionCoefY ~ 1 + Mass * Vision * Vibration + (1|subj), data = copv)
#
# anova(m0, m1, m2, m3, m4)
# print(summary(m4))

# g5 = ggplot(copv, aes(Vibration, DiffusionCoefY, colour = Vision, fill = Vision)) +
#   stat_summary(fun.data = 'mean_cl_boot', position = position_dodge(),geom = 'bar') +
#   stat_summary(fun.data = 'mean_cl_boot', position = position_dodge(0.9), geom = 'errorbar', colour =
# print(g5)

# model for velocity
m0 = lmerTest::lmer(AMMVy ~ 1 + ( 1|subj ), data = copv)
m1 = lmerTest::lmer(AMMVy ~ 1 + Vibration + (1|subj), data = copv)
m2 = lmerTest::lmer(AMMVy ~ 1 + Vibration + Vision + (1|subj), data = copv)
# m3 = lmerTest::lmer(AMMVy ~ 1 + Vibration * Vision + (1|subj), data = copv)
# m4 = lmerTest::lmer(AMMVy ~ 1 + Cause + Vision * Vibration + (1|subj), data = copv)
```

```r
# m4 = lmerTest::lmer(AMMVy ~ 1 + TCThresholdGroup + Vision * Vibration + (1|subj), data = copv)
# m4 = lmerTest::lmer(AMMVy ~ 1 + Vibration * Vision + Mass + (1|subj), data = copv)
# m5 = lmerTest::lmer(AMMVy ~ 1 + Vibration * Vision + Mass + TCThresholdGroup + (1|subj), data = copv)
# m5 = lmerTest::lmer(AMMVy ~ 1 + Vibration * Vision + Mass + Mass:Vibration + (1|subj), data = copv)
# m6 = lmerTest::lmer(AMMVy ~ 1 + Vibration * Vision + Mass + Mass:Vibration + Mass:Vision +(1|subj), d
# m6 = lmerTest::lmer(AMMVy ~ 1 + Mass * Vision * Vibration + (1|subj), data = copv)

anova(m0, m1, m2)
```

```
## refitting model(s) with ML (instead of REML)

## Data: copv
## Models:
## m0: AMMVy ~ 1 + (1 | subj)
## m1: AMMVy ~ 1 + Vibration + (1 | subj)
## m2: AMMVy ~ 1 + Vibration + Vision + (1 | subj)
##    npar     AIC     BIC logLik deviance   Chisq Df Pr(>Chisq)
## m0    3 -555.11 -546.91 280.56  -561.11
## m1    5 -553.36 -539.67 281.68  -563.36  2.2408  2     0.3261
## m2    6 -602.28 -585.86 307.14  -614.28 50.9221  1  9.611e-13 ***
## ---
## Signif. codes:  0 '***' 0.001 '**' 0.01 '*' 0.05 '.' 0.1 ' ' 1
```

```r
print(summary(m2))
```

```
## Linear mixed model fit by REML. t-tests use Satterthwaite's method [
## lmerModLmerTest]
## Formula: AMMVy ~ 1 + Vibration + Vision + (1 | subj)
##    Data: copv
##
## REML criterion at convergence: -575.6
##
## Scaled residuals:
##      Min       1Q   Median       3Q      Max
## -2.29829 -0.64096 -0.06509  0.46867  3.15371
##
## Random effects:
##  Groups   Name        Variance  Std.Dev.
##  subj     (Intercept) 0.0006236 0.02497
##  Residual             0.0001634 0.01278
## Number of obs: 114, groups:  subj, 19
##
## Fixed effects:
##              Estimate Std. Error        df t value Pr(>|t|)
## (Intercept)  0.071324   0.006209 22.731739  11.487 6.09e-11 ***
## Vibration2   0.005676   0.002932 92.000000   1.936    0.056 .
## Vibration3   0.002649   0.002932 92.000000   0.903    0.369
## Vision2      0.019339   0.002394 92.000000   8.077 2.48e-12 ***
## ---
## Signif. codes:  0 '***' 0.001 '**' 0.01 '*' 0.05 '.' 0.1 ' ' 1
##
## Correlation of Fixed Effects:
##            (Intr) Vbrtn2 Vbrtn3
## Vibration2 -0.236
## Vibration3 -0.236  0.500
```

```
## Vision2    -0.193  0.000  0.000
```

```
g6 = ggplot(copv, aes(Vision, AMMVy, colour = Vision, fill = Vision)) +
  stat_summary(fun.data = 'mean_cl_boot', position = position_dodge(),geom = 'bar') +
  stat_summary(fun.data = 'mean_cl_boot', position = position_dodge(0.9), geom = 'errorbar', colour = 'l
print(g6)
```

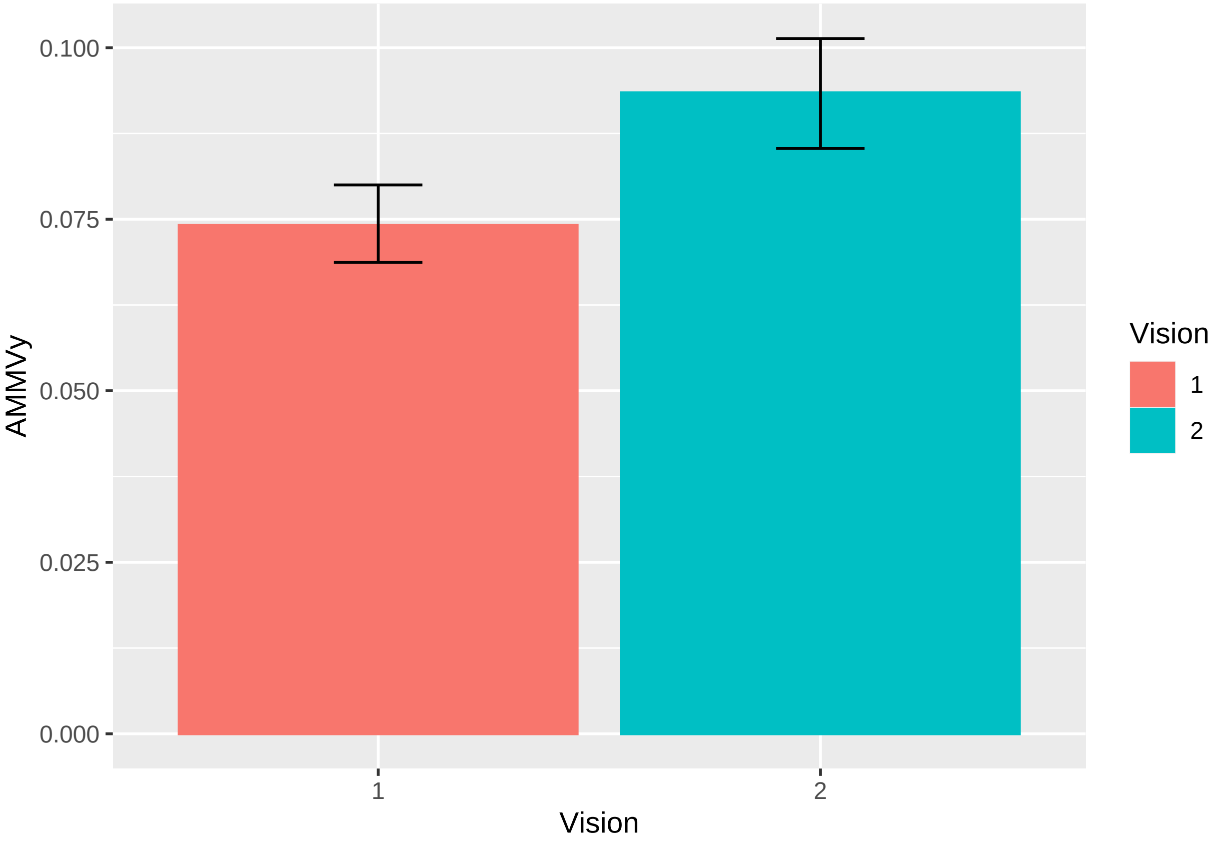


```
# velocityY -----------------------------------------------------------------
m0 = lmerTest::lmer(VelocityY ~ 1 + ( 1|subj ), data = copv)
m1 = lmerTest::lmer(VelocityY ~ 1 + Vibration + (1|subj), data = copv)
m2 = lmerTest::lmer(VelocityY ~ 1 + Vibration + Vision + (1|subj), data = copv)
# m3 = lmerTest::lmer(VelocityY ~ 1 + Vibration * Vision + (1|subj), data = copv)
# m4 = lmerTest::lmer(VelocityY ~ 1 + Cause + Vision + Vibration + (1|subj), data = copv)
# m4 = lmerTest::lmer(VelocityY ~ 1 + TCThresholdGroup + Vision + Vibration + (1|subj), data = copv)
# m4 = lmerTest::lmer(VelocityY ~ 1 + Vibration + Vision + Mass + (1|subj), data = copv)

anova(m0, m1, m2)
```

```
## refitting model(s) with ML (instead of REML)
```

```
## Data: copv
## Models:
## m0: VelocityY ~ 1 + (1 | subj)
## m1: VelocityY ~ 1 + Vibration + (1 | subj)
## m2: VelocityY ~ 1 + Vibration + Vision + (1 | subj)
##    npar     AIC     BIC logLik deviance   Chisq Df Pr(>Chisq)
## m0    3 -829.82 -821.61 417.91  -835.82
## m1    5 -828.37 -814.69 419.18  -838.37  2.5511  2     0.2793
## m2    6 -873.16 -856.75 442.58  -885.16 46.7943  1  7.884e-12 ***
```

```
## ---
## Signif. codes:  0 '***' 0.001 '**' 0.01 '*' 0.05 '.' 0.1 ' ' 1
```

```r
print(summary(m2))
```

```
## Linear mixed model fit by REML. t-tests use Satterthwaite's method [
## lmerModLmerTest]
## Formula: VelocityY ~ 1 + Vibration + Vision + (1 | subj)
##    Data: copv
##
## REML criterion at convergence: -837
##
## Scaled residuals:
##     Min      1Q  Median      3Q     Max
## -2.4323 -0.5841 -0.0482  0.3784  3.6156
##
## Random effects:
##  Groups   Name        Variance  Std.Dev.
##  subj     (Intercept) 6.049e-05 0.007778
##  Residual             1.505e-05 0.003880
## Number of obs: 114, groups:  subj, 19
##
## Fixed effects:
##              Estimate Std. Error        df t value Pr(>|t|)
## (Intercept) 1.927e-02  1.927e-03 2.249e+01  10.001 9.59e-10 ***
## Vibration2  1.801e-03  8.901e-04 9.200e+01   2.024   0.0459 *
## Vibration3  9.299e-04  8.901e-04 9.200e+01   1.045   0.2989
## Vision2     5.561e-03  7.268e-04 9.200e+01   7.652 1.89e-11 ***
## ---
## Signif. codes:  0 '***' 0.001 '**' 0.01 '*' 0.05 '.' 0.1 ' ' 1
##
## Correlation of Fixed Effects:
##            (Intr) Vbrtn2 Vbrtn3
## Vibration2 -0.231
## Vibration3 -0.231  0.500
## Vision2    -0.189  0.000  0.000
```

```r
g7 = ggplot(copv, aes(Vision, VelocityY, colour = Vision, fill = Vision)) +
  stat_summary(fun.data = 'mean_cl_boot', position = position_dodge(),geom = 'bar') +
  stat_summary(fun.data = 'mean_cl_boot', position = position_dodge(0.9), geom = 'errorbar', colour = '[illegible]
print(g7)
```

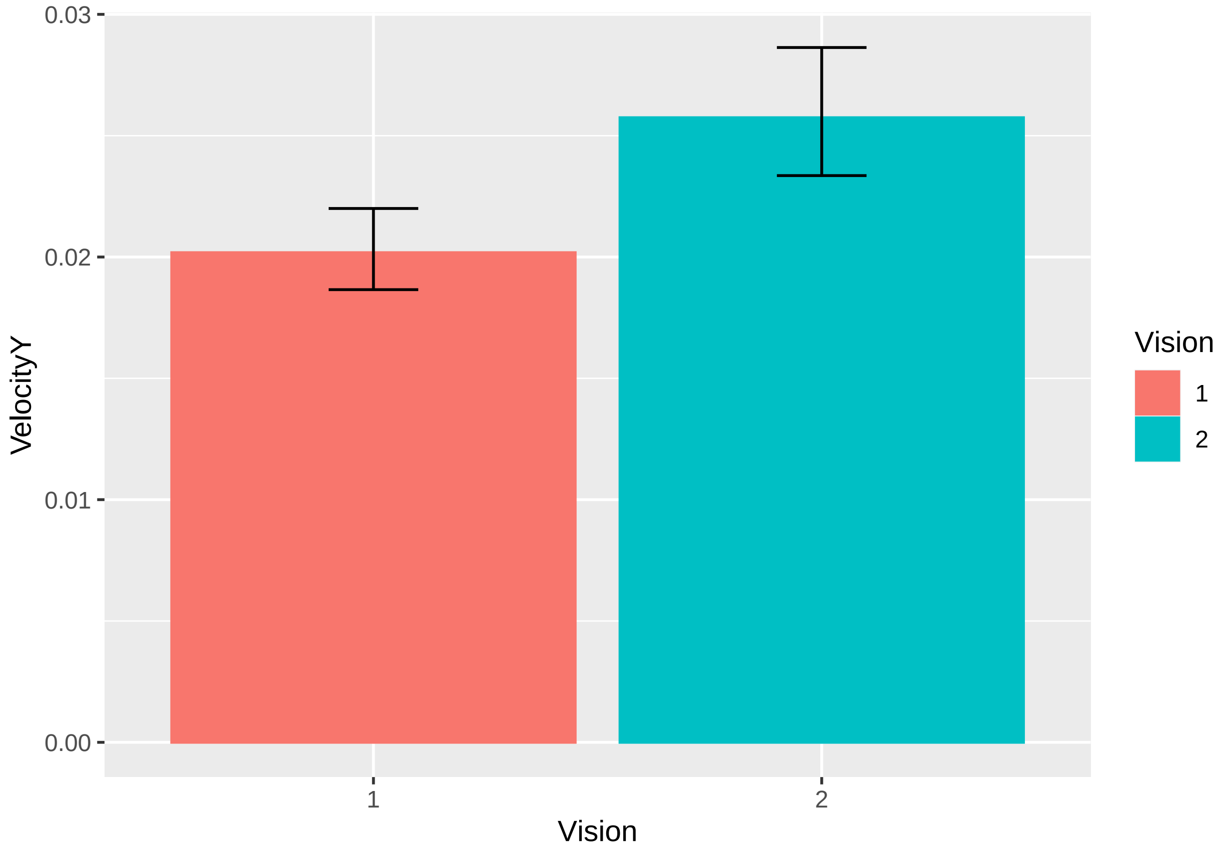


```
# Dys --------------------------------------------------------------------------
m0 = lmerTest::lmer(Dys ~ 1 + ( 1|subj ), data = copv)
m1 = lmerTest::lmer(Dys ~ 1 + Vibration + (1|subj), data = copv)
m2 = lmerTest::lmer(Dys ~ 1 + Vibration + Vision + (1|subj), data = copv)
# m3 = lmerTest::lmer(Dys ~ 1 + Vibration * Vision + (1|subj), data = copv)
# m4 = lmerTest::lmer(Dys ~ 1 + Cause + Vision + Vibration + (1|subj), data = copv)
# m4 = lmerTest::lmer(Dys ~ 1 + TCThresholdGroup + Vision + Vibration + (1|subj), data = copv)
# m4 = lmerTest::lmer(Dys ~ 1 + Vibration + Vision + Mass + (1|subj), data = copv)

anova(m0, m1, m2)
```

```
## refitting model(s) with ML (instead of REML)
```

```
## Data: copv
## Models:
## m0: Dys ~ 1 + (1 | subj)
## m1: Dys ~ 1 + Vibration + (1 | subj)
## m2: Dys ~ 1 + Vibration + Vision + (1 | subj)
##    npar     AIC     BIC logLik deviance  Chisq Df Pr(>Chisq)
## m0    3 -1808.5 -1800.3 907.28  -1814.5
## m1    5 -1807.7 -1794.0 908.84  -1817.7  3.127  2     0.2094
## m2    6 -1821.7 -1805.3 916.87  -1833.7 16.059  1   6.14e-05 ***
## ---
## Signif. codes:  0 '***' 0.001 '**' 0.01 '*' 0.05 '.' 0.1 ' ' 1
```

```
print(summary(m2))
```

```
## Linear mixed model fit by REML. t-tests use Satterthwaite's method [
```

```
## lmerModLmerTest]
## Formula: Dys ~ 1 + Vibration + Vision + (1 | subj)
##    Data: copv
##
## REML criterion at convergence: -1752.2
##
## Scaled residuals:
##     Min      1Q  Median      3Q     Max
## -3.3656 -0.2939 -0.0792  0.2681  6.0585
##
## Random effects:
##  Groups   Name        Variance  Std.Dev.
##  subj     (Intercept) 1.056e-08 1.028e-04
##  Residual             3.901e-09 6.245e-05
## Number of obs: 114, groups:  subj, 19
##
## Fixed effects:
##              Estimate Std. Error        df t value Pr(>|t|)
## (Intercept) 2.883e-05  2.632e-05 2.466e+01   1.095   0.2839
## Vibration2  2.699e-05  1.433e-05 9.200e+01   1.884   0.0627 .
## Vibration3  1.734e-05  1.433e-05 9.200e+01   1.210   0.2292
## Vision2     4.815e-05  1.170e-05 9.200e+01   4.116 8.39e-05 ***
## ---
## Signif. codes:  0 '***' 0.001 '**' 0.01 '*' 0.05 '.' 0.1 ' ' 1
##
## Correlation of Fixed Effects:
##            (Intr) Vbrtn2 Vbrtn3
## Vibration2 -0.272
## Vibration3 -0.272  0.500
## Vision2    -0.222  0.000  0.000
```

```
g8 = ggplot(copv, aes(Vision, Dys, colour = Vision, fill = Vision)) +
  stat_summary(fun.data = 'mean_cl_boot', position = position_dodge(),geom = 'bar') +
  stat_summary(fun.data = 'mean_cl_boot', position = position_dodge(0.9), geom = 'errorbar', colour = '[illegible]
print(g8)
```

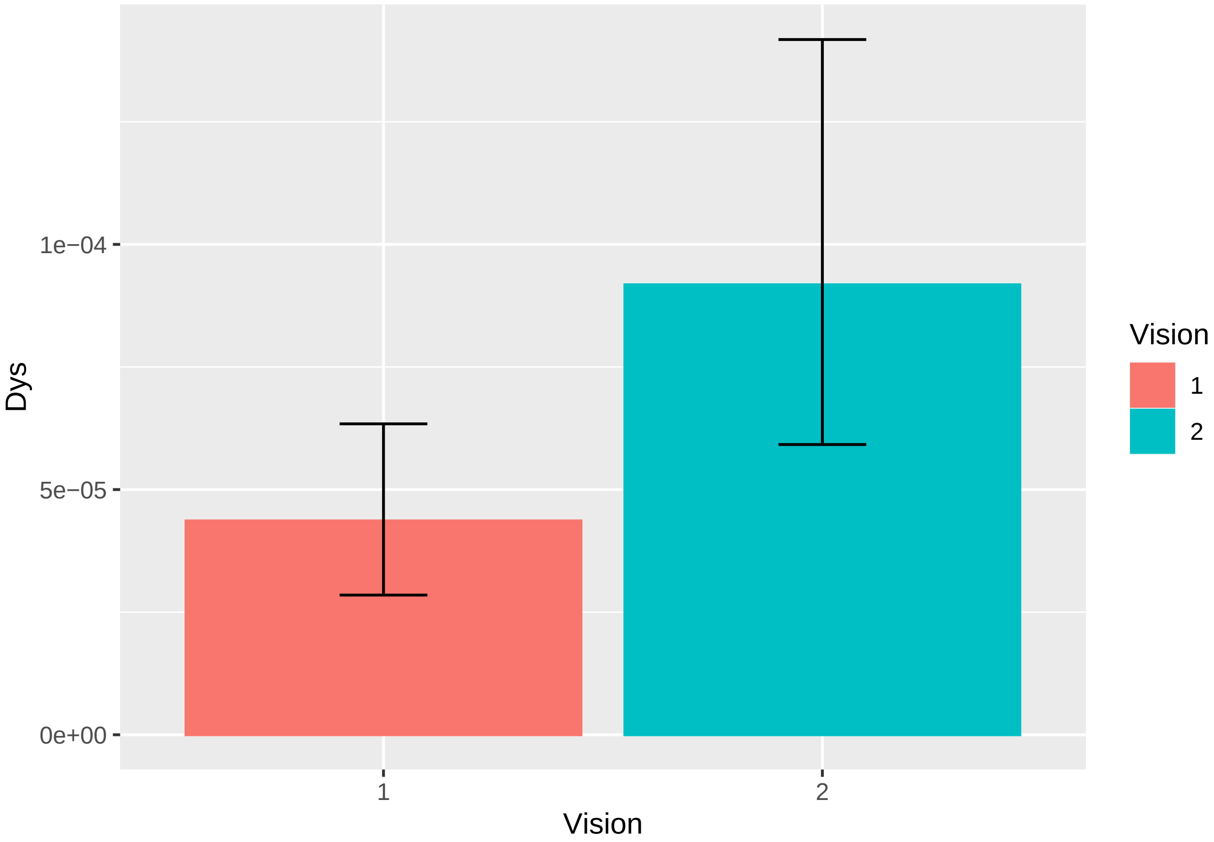


```
# Dyl ---------------------------------------------------------------------

m0 = lmerTest::lmer(Dyl ~ 1 + ( 1|subj ), data = copv)
m1 = lmerTest::lmer(Dyl ~ 1 + Vibration + (1|subj), data = copv)
m2 = lmerTest::lmer(Dyl ~ 1 + Vibration + Vision + (1|subj), data = copv)
# m3 = lmerTest::lmer(Dyl ~ 1 + Vibration * Vision + (1|subj), data = copv)
# m4 = lmerTest::lmer(Dyl ~ 1 + Cause + Vision + Vibration + (1|subj), data = copv)
# m4 = lmerTest::lmer(Dyl ~ 1 + TCThresholdGroup + Vision + Vibration + (1|subj), data = copv)
# m4 = lmerTest::lmer(Dyl ~ 1 + Vibration + Vision + Mass + (1|subj), data = copv)

anova(m0, m1, m2)
```

```
## refitting model(s) with ML (instead of REML)
```

```
## Data: copv
## Models:
## m0: Dyl ~ 1 + (1 | subj)
## m1: Dyl ~ 1 + Vibration + (1 | subj)
## m2: Dyl ~ 1 + Vibration + Vision + (1 | subj)
##    npar     AIC     BIC logLik deviance  Chisq Df Pr(>Chisq)
## m0    3 -2872.5 -2864.3 1439.2  -2878.5
## m1    5 -2870.5 -2856.8 1440.2  -2880.5 2.0184  2   0.364506
## m2    6 -2875.2 -2858.8 1443.6  -2887.2 6.7361  1   0.009448 **
## ---
## Signif. codes:  0 '***' 0.001 '**' 0.01 '*' 0.05 '.' 0.1 ' ' 1
```

```
print(summary(m2))
```

```
## Linear mixed model fit by REML. t-tests use Satterthwaite's method [
```

```
## lmerModLmerTest]
## Formula: Dyl ~ 1 + Vibration + Vision + (1 | subj)
##    Data: copv
##
## REML criterion at convergence: -2768
##
## Scaled residuals:
##     Min      1Q  Median      3Q     Max
## -1.4857 -0.4995 -0.1346  0.1793  5.5442
##
## Random effects:
##  Groups   Name        Variance  Std.Dev.
##  subj     (Intercept) 8.886e-14 2.981e-07
##  Residual             5.423e-13 7.364e-07
## Number of obs: 114, groups:  subj, 19
##
## Fixed effects:
##               Estimate Std. Error         df t value Pr(>|t|)
## (Intercept)  3.307e-07  1.540e-07  1.139e+00   2.148    0.253
## Vibration2  -1.843e-07  1.690e-07  1.151e+00  -1.091    0.453
## Vibration3  -2.333e-07  1.690e-07  1.151e+00  -1.381    0.376
## Vision2      3.587e-07  1.379e-07  1.151e+00   2.600    0.207
##
## Correlation of Fixed Effects:
##            (Intr) Vbrtn2 Vbrtn3
## Vibration2 -0.549
## Vibration3 -0.549  0.500
## Vision2    -0.448  0.000  0.000
```

```
g9 = ggplot(copv, aes(Vision, Dyl, colour = Vision, fill = Vision)) +
  stat_summary(fun.data = 'mean_cl_boot', position = position_dodge(),geom = 'bar') +
  stat_summary(fun.data = 'mean_cl_boot', position = position_dodge(0.9), geom = 'errorbar', colour = 'l
print(g9)
```

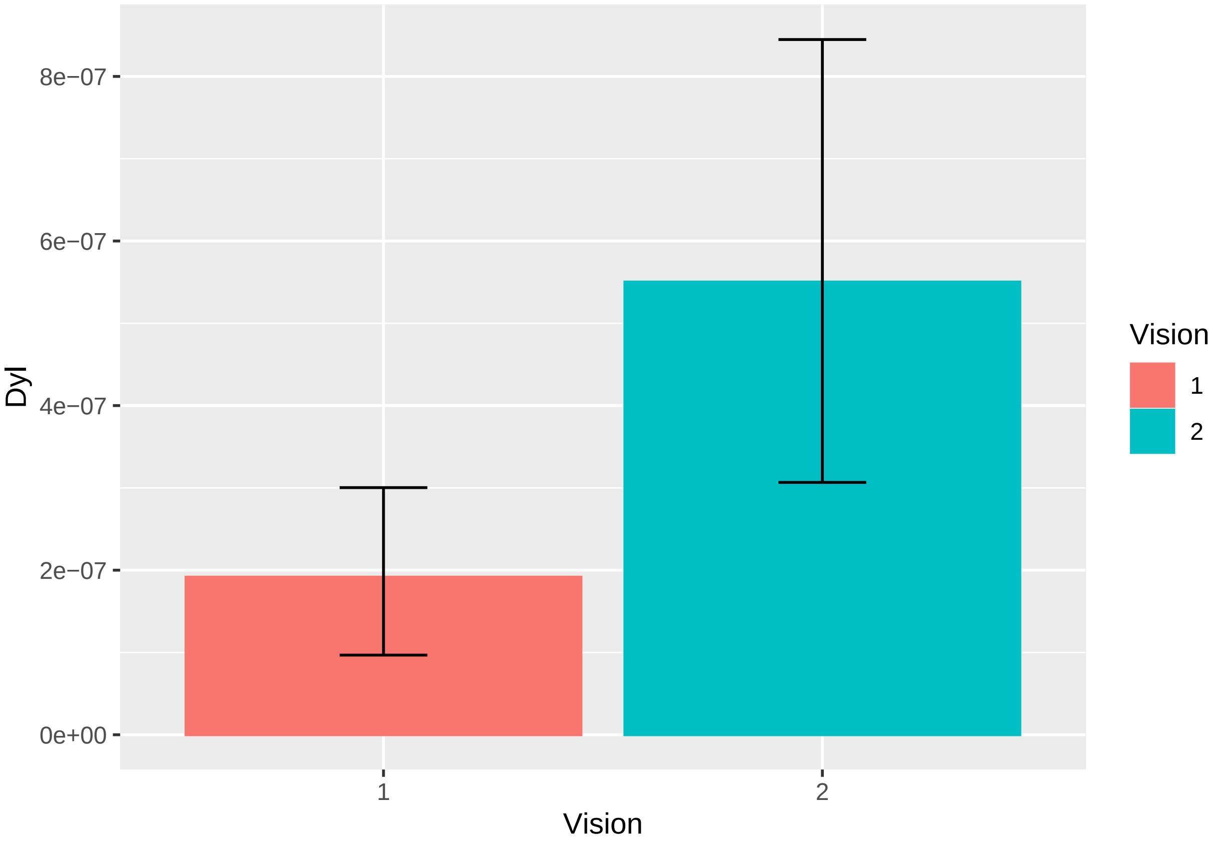


```r
# Hys ----------------------------------------------------------------------

m0 = lmerTest::lmer(Hys ~ 1 + ( 1|subj ), data = copv)
m1 = lmerTest::lmer(Hys ~ 1 + Vibration + (1|subj), data = copv)
m2 = lmerTest::lmer(Hys ~ 1 + Vibration + Vision + (1|subj), data = copv)
# m3 = lmerTest::lmer(Hys ~ 1 + Vibration * Vision + (1|subj), data = copv)
# m4 = lmerTest::lmer(Hys ~ 1 + Cause + Vision + Vibration + (1|subj), data = copv)
# m4 = lmerTest::lmer(Hys ~ 1 + TCThresholdGroup + Vision + Vibration + (1|subj), data = copv)
m4 = lmerTest::lmer(Hys ~ 1 + Vibration + Vision + Mass + (1|subj), data = copv)
# m5 = lmerTest::lmer(Hys ~ 1 + Vibration + Vision + zMass + zMass2 + (1|subj), data = copv)
#m6 = lmerTest::lmer(Hys ~ 1 + Vibration*Mass + Vision*Mass + (1|subj), data = copv)

anova(m0, m1, m2, m4)
```

```
## refitting model(s) with ML (instead of REML)

## Data: copv
## Models:
## m0: Hys ~ 1 + (1 | subj)
## m1: Hys ~ 1 + Vibration + (1 | subj)
## m2: Hys ~ 1 + Vibration + Vision + (1 | subj)
## m4: Hys ~ 1 + Vibration + Vision + Mass + (1 | subj)
##    npar     AIC     BIC logLik deviance   Chisq Df Pr(>Chisq)
## m0    3 -305.22 -297.02 155.61  -311.22
## m1    5 -303.52 -289.84 156.76  -313.52  2.2991  2   0.316784
## m2    6 -328.29 -311.87 170.14  -340.29 26.7632  1    2.3e-07 ***
## m4    7 -334.20 -315.04 174.10  -348.20  7.9082  1   0.004921 **
## ---
```

```
## Signif. codes:  0 '***' 0.001 '**' 0.01 '*' 0.05 '.' 0.1 ' ' 1
```

```r
print(summary(m2))
```

```
## Linear mixed model fit by REML. t-tests use Satterthwaite's method [
## lmerModLmerTest]
## Formula: Hys ~ 1 + Vibration + Vision + (1 | subj)
##    Data: copv
##
## REML criterion at convergence: -310.9
##
## Scaled residuals:
##     Min      1Q  Median      3Q     Max
## -2.7310 -0.4777  0.1166  0.6411  1.8989
##
## Random effects:
##  Groups   Name        Variance Std.Dev.
##  subj     (Intercept) 0.002948 0.05430
##  Residual             0.002112 0.04596
## Number of obs: 114, groups:  subj, 19
##
## Fixed effects:
##              Estimate Std. Error        df t value Pr(>|t|)
## (Intercept)  0.665633   0.015142 30.745090  43.959  < 2e-16 ***
## Vibration2   0.014587   0.010543 92.000000   1.384    0.170
## Vibration3  -0.002163   0.010543 92.000000  -0.205    0.838
## Vision2      0.047101   0.008608 92.000000   5.471 3.82e-07 ***
## ---
## Signif. codes:  0 '***' 0.001 '**' 0.01 '*' 0.05 '.' 0.1 ' ' 1
##
## Correlation of Fixed Effects:
##            (Intr) Vbrtn2 Vbrtn3
## Vibration2 -0.348
## Vibration3 -0.348  0.500
## Vision2    -0.284  0.000  0.000
```

```r
g10 = ggplot(copv, aes(Vision, Hys, colour = Vibration, fill = Vibration)) +
  stat_summary(fun.data = 'mean_cl_boot', position = position_dodge(),geom = 'bar') +
  stat_summary(fun.data = 'mean_cl_boot', position = position_dodge(0.9), geom = 'errorbar', colour = 'l
print(g10)
```

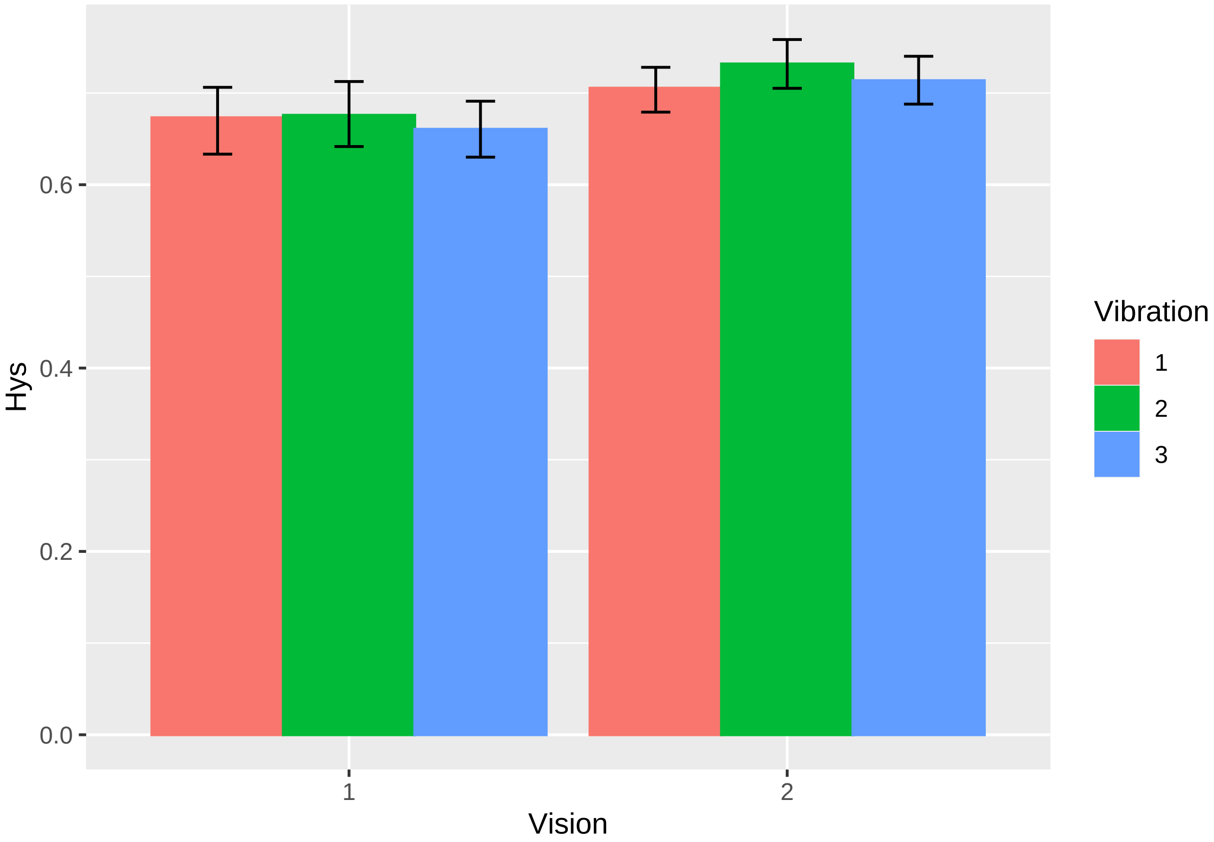


```
g11 = ggplot(copv, aes(Mass, Hys)) +
  geom_point() +
  geom_smooth()
print(g11)
```

```
## `geom_smooth()` using method = 'loess' and formula 'y ~ x'
```

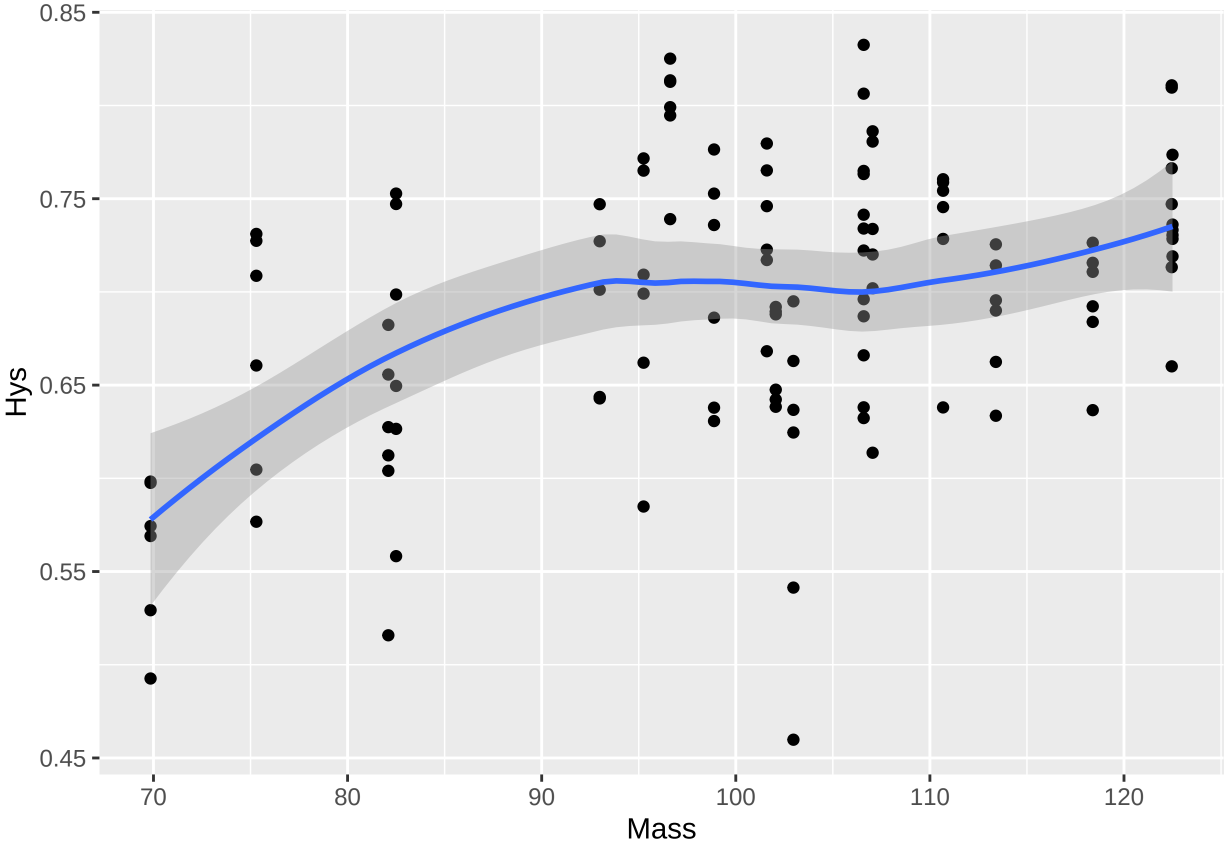


```
# Hyl ------------------------------------------------------------------

# m0 = lmerTest::lmer(Hyl ~ 1 + ( 1|subj ), data = copv)
# m1 = lmerTest::lmer(Hyl ~ 1 + Vibration + (1|subj), data = copv)
# m2 = lmerTest::lmer(Hyl ~ 1 + Vibration + Vision + (1|subj), data = copv)
#
# print(anova(m0, m1, m2))
# print(summary(m2))
#
#
# g12 = ggplot(copv, aes(Vision, Hyl, colour = Vibration, fill = Vibration)) +
#   stat_summary(fun.data = 'mean_cl_boot', position = position_dodge(),geom = 'bar') +
#   stat_summary(fun.data = 'mean_cl_boot', position = position_dodge(0.9), geom = 'errorbar', colour =
# print(g12)


# critY ----------------------------------------------------------------
# m0 = lmerTest::lmer(critY ~ 1 + ( 1|subj ), data = copv)
# m1 = lmerTest::lmer(critY ~ 1 + Vibration + (1|subj), data = copv)
# m2 = lmerTest::lmer(critY ~ 1 + Vibration + Vision + (1|subj), data = copv)
# m3 = lmerTest::lmer(critY ~ 1 + Vibration * Vision + (1|subj), data = copv)


# dy2 ------------------------------------------------------------------

m0 = lmerTest::lmer(dy2 ~ 1 + ( 1|subj ), data = copv)
m1 = lmerTest::lmer(dy2 ~ 1 + Vibration + (1|subj), data = copv)
```

```r
m2 = lmerTest::lmer(dy2 ~ 1 + Vibration + Vision + (1|subj), data = copv)
# m3 = lmerTest::lmer(dy2 ~ 1 + Vibration * Vision + (1|subj), data = copv)
# m4 = lmerTest::lmer(dy2 ~ 1 + Cause + Vision + Vibration + (1|subj), data = copv)
#m4 = lmerTest::lmer(dy2 ~ 1 + TCThresholdGroup + Vision + Vibration + (1|subj), data = copv)
#m4 = lmerTest::lmer(dy2 ~ 1 + Vibration + Vision + Mass + (1|subj), data = copv)

anova(m0, m1, m2)
```

```
## refitting model(s) with ML (instead of REML)

## Data: copv
## Models:
## m0: dy2 ~ 1 + (1 | subj)
## m1: dy2 ~ 1 + Vibration + (1 | subj)
## m2: dy2 ~ 1 + Vibration + Vision + (1 | subj)
##    npar     AIC     BIC logLik deviance  Chisq Df Pr(>Chisq)
## m0    3 -1689.1 -1680.9 847.54  -1695.1
## m1    5 -1687.2 -1673.5 848.60  -1697.2 2.1280  2   0.345072
## m2    6 -1694.6 -1678.2 853.32  -1706.6 9.4243  1   0.002141 **
## ---
## Signif. codes:  0 '***' 0.001 '**' 0.01 '*' 0.05 '.' 0.1 ' ' 1
```

```r
print(summary(m2))
```

```
## Linear mixed model fit by REML. t-tests use Satterthwaite's method [
## lmerModLmerTest]
## Formula: dy2 ~ 1 + Vibration + Vision + (1 | subj)
##    Data: copv
##
## REML criterion at convergence: -1629.4
##
## Scaled residuals:
##     Min      1Q  Median      3Q     Max
## -3.0807 -0.2402 -0.0207  0.1497  6.4771
##
## Random effects:
##  Groups   Name        Variance  Std.Dev.
##  subj     (Intercept) 2.389e-08 0.0001545
##  Residual             1.257e-08 0.0001121
## Number of obs: 114, groups:  subj, 19
##
## Fixed effects:
##              Estimate Std. Error        df t value Pr(>|t|)
## (Intercept) 3.984e-05  4.121e-05 2.745e+01   0.967  0.34209
## Vibration2  3.529e-05  2.572e-05 9.200e+01   1.372  0.17338
## Vibration3  3.206e-05  2.572e-05 9.200e+01   1.247  0.21574
## Vision2     6.504e-05  2.100e-05 9.200e+01   3.098  0.00259 **
## ---
## Signif. codes:  0 '***' 0.001 '**' 0.01 '*' 0.05 '.' 0.1 ' ' 1
##
## Correlation of Fixed Effects:
##            (Intr) Vbrtn2 Vbrtn3
## Vibration2 -0.312
## Vibration3 -0.312  0.500
## Vision2    -0.255  0.000  0.000
```

```
g10 = ggplot(copv, aes(Vision, dy2, colour = Vibration, fill = Vibration)) +
  stat_summary(fun.data = 'mean_cl_boot', position = position_dodge(),geom = 'bar') +
  stat_summary(fun.data = 'mean_cl_boot', position = position_dodge(0.9), geom = 'errorbar', colour = '[illegible]
print(g10)
```

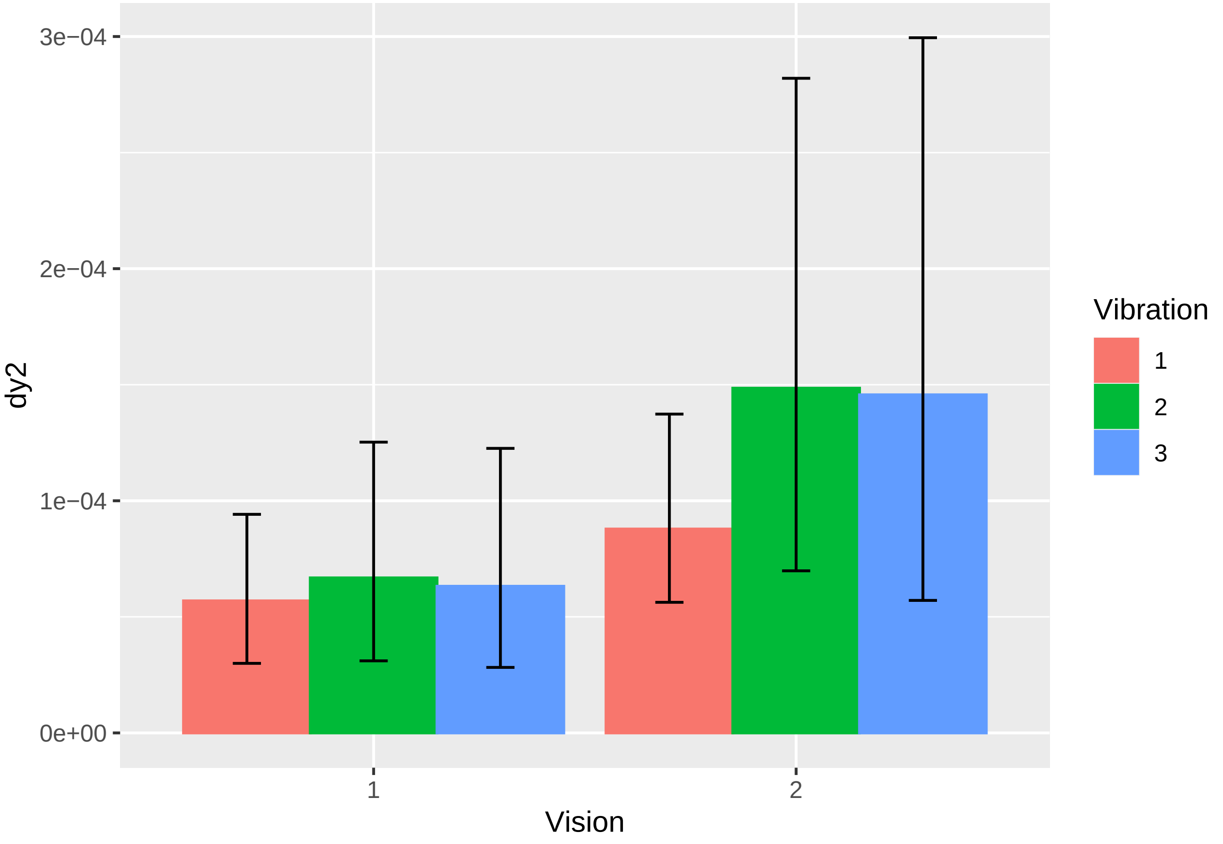

Supplement: Supplementary file 3 [file Data_Sheet_2.PDF]
