# Supplementary figures and images for: Subthreshold Vibration Influences Standing Balance but Has Unclear Impact on Somatosensation in Persons With Transtibial Amputations
Source: Front Physiol. 2022 Feb 2;13:810079. doi: 10.3389/fphys.2022.810079 (PMC8847287; doi:10.3389/fphys.2022.810079)

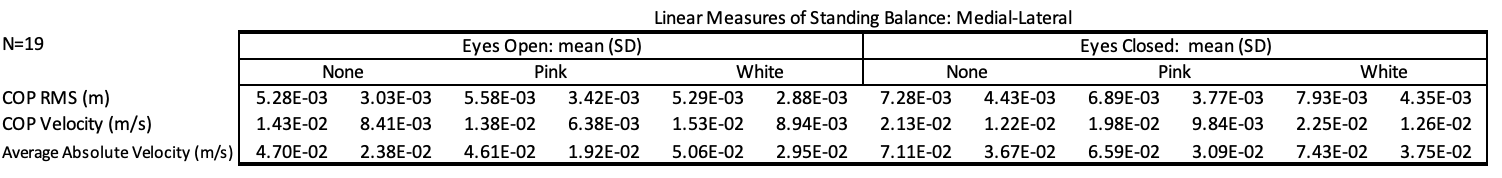

Supplement: Supplementary file 4 [file Image_1.PNG]

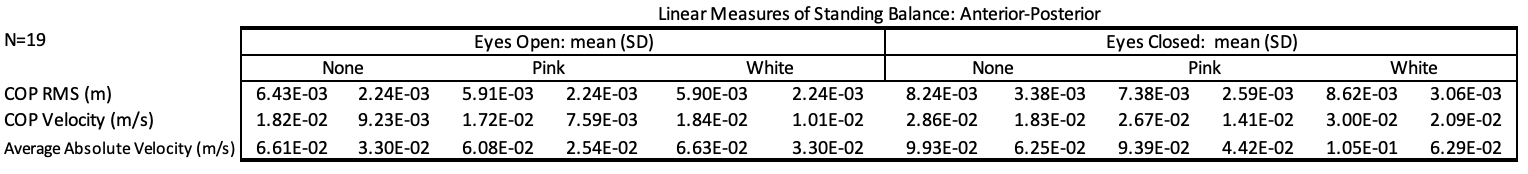

Supplement: Supplementary file 5 [file Image_2.PNG]

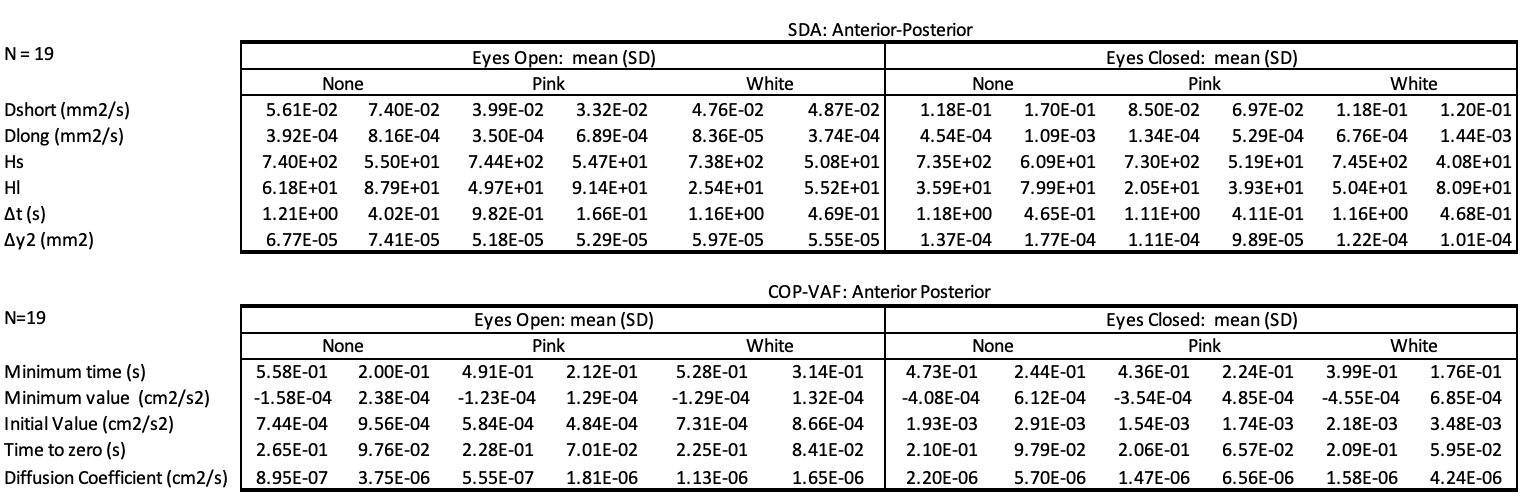

Supplement: Supplementary file 6 [file Image_3.PNG]

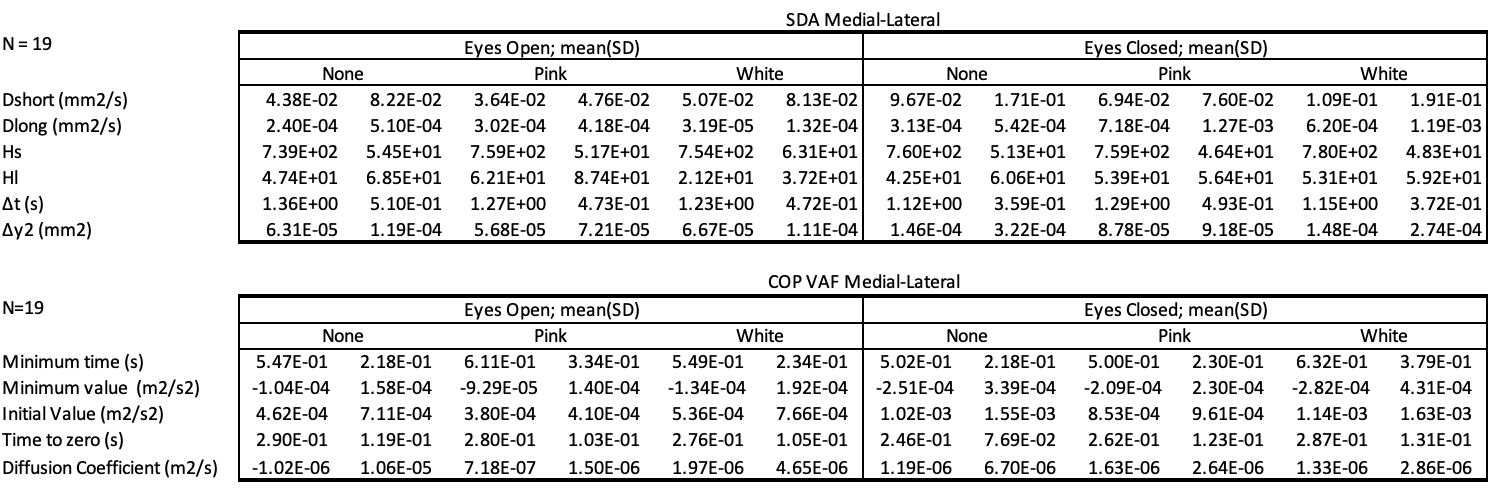

Supplement: Supplementary file 7 [file Image_4.PNG]
